# Human-Specific Histone Methylation Signatures at Transcription Start Sites in Prefrontal Neurons

**DOI:** 10.1371/journal.pbio.1001427

**Published:** 2012-11-20

**Authors:** Hennady P. Shulha, Jessica L. Crisci, Denis Reshetov, Jogender S. Tushir, Iris Cheung, Rahul Bharadwaj, Hsin-Jung Chou, Isaac B. Houston, Cyril J. Peter, Amanda C. Mitchell, Wei-Dong Yao, Richard H. Myers, Jiang-fan Chen, Todd M. Preuss, Evgeny I. Rogaev, Jeffrey D. Jensen, Zhiping Weng, Schahram Akbarian

**Affiliations:** 1 Program in Bioinformatics and Integrative Biology, University of Massachusetts Medical School, Worcester, Massachusetts, United States of America; 2 Department of Human Genetics and Genomics, Vavilov Institute of General Genetics, Moscow, Russian Federation; 3 Brudnick Neuropsychiatric Research Institute, University of Massachusetts Medical School, Worcester, Massachusetts, United States of America; 4 New England Primate Center, Southboro, Massachusetts, United States of America; 5 Department of Neurology, Boston University, Boston, Massachusetts, United States of America; 6 Yerkes National Primate Research Center/Emory University, Atlanta, Georgia, United States of America; 7 Research Center of Mental Health, Russian Academy of Medical Sciences, Moscow, Russian Federation; 8 School of Life Sciences, Ecole Polytechnique Fédérale de Lausanne, Lausanne, Switzerland; 9 Faculty of Bioengineering and Bioinformatics, Lomonosov Moscow State University, Russian Federation; 10 Departments of Psychiatry and Neuroscience, Friedman Brain Institute, Mount Sinai School of Medicine, New York, New York, United States of America; Massey University, New Zealand

## Abstract

Cognitive abilities and disorders unique to humans are thought to result from adaptively driven changes in brain transcriptomes, but little is known about the role of cis-regulatory changes affecting transcription start sites (TSS). Here, we mapped in human, chimpanzee, and macaque prefrontal cortex the genome-wide distribution of histone H3 trimethylated at lysine 4 (H3K4me3), an epigenetic mark sharply regulated at TSS, and identified 471 sequences with human-specific enrichment or depletion. Among these were 33 loci selectively methylated in neuronal but not non-neuronal chromatin from children and adults, including TSS at *DPP10* (2q14.1), *CNTN4* and *CHL1* (3p26.3), and other neuropsychiatric susceptibility genes. Regulatory sequences at *DPP10* and additional loci carried a strong footprint of hominid adaptation, including elevated nucleotide substitution rates and regulatory motifs absent in other primates (including archaic hominins), with evidence for selective pressures during more recent evolution and adaptive fixations in modern populations. Chromosome conformation capture at two neurodevelopmental disease loci, 2q14.1 and 16p11.2, revealed higher order chromatin structures resulting in physical contact of multiple human-specific H3K4me3 peaks spaced 0.5–1 Mb apart, in conjunction with a novel *cis*-bound antisense RNA linked to Polycomb repressor proteins and downregulated *DPP10* expression. Therefore, coordinated epigenetic regulation via newly derived TSS chromatin could play an important role in the emergence of human-specific gene expression networks in brain that contribute to cognitive functions and neurological disease susceptibility in modern day humans.

## Abstract 2

Mapping histone methylation landscapes in neurons from human, chimpanzee, and macaque brains reveals coordinated, human-specific epigenetic regulation at hundreds of regulatory sequences.

## Author Summary

Primate and human genomes comprise billions of base pairs, but we are unlikely to gain a deeper understanding of brain functions unique to human (including cognitive abilities and psychiatric diseases) merely by comparing linear DNA sequences. Such determinants of species-specific function might instead be found in the so-called “epigenetic” characteristics of genomic regions; differences in the protein-packaged chromatin state in which genomic DNA exists in the cell. Here, we examine neurons from the prefrontal cortex, a brain region closely associated with the evolution of the primate brain, and identify hundreds of short DNA sequences defined by human-specific changes in chromatin structure and function when compared to non-human primates. These changes included species-specific regulation of methylation marks on the histone proteins around which genomic DNA is wrapped. Sequences subject to human-specific epigenetic regulation showed significant spatial clustering, and despite being separated by hundreds of thousands of base pairs on the linear genome, were in direct physical contact with each other through chromosomal looping and other higher order chromatin features. This observation raises the intriguing possibility that coordinated epigenetic regulation via newly derived chromatin features at gene transcription start sites could play an important role in the emergence of human-specific gene expression networks in the brain. Finally, we identified a strong genetic footprint of hominid evolution in a small subset of transcription start sites defined by human-specific gains in histone methylation, with particularly strong enrichment in prefrontal cortex neurons. For example, the base pair sequence of *DPP10* (a gene critically important for normal human brain development) not only showed distinct human-specific changes, but also evidence for more recent selective pressures within the human population.

## Introduction

Cognitive abilities and psychiatric diseases unique to modern humans could be based on genomic features distinguishing our brain cells, including neurons, from those of other primates. Because protein coding sequences for synaptic and other neuron-specific genes are highly conserved across the primate tree [1],[2], a significant portion of hominid evolution could be due to DNA sequence changes involving regulatory and non-coding regions at the 5′ end of genes [3],[4]. Quantifying these differences, however, is ultimately a daunting task, considering that, for example, the chimpanzee–human genome comparison alone reveals close to 35×10^6^ single bp and 5×10^6^ multi-bp substitutions and insertion/deletion events [3]. While a large majority of these are likely to reflect genetic drift and are deemed “non-consequential” with respect to fitness, the challenge is to identify the small subset of regulatory sequence alterations impacting brain function and behavior.

Here, we combine comparative genomics and population genetics with genome-scale comparisons for histone H3-trimethyl-lysine 4 (H3K4me3), an epigenetic mark sharply regulated at transcription start sites (TSS) and the 5′ end of transcriptional units in brain and other tissues [5]–[8] that is stably maintained in brain specimens collected postmortem [7],[9]. Our rationale to focus on TSS chromatin was also guided by the observation that the human brain, and in particular the cerebral cortex, shows distinct changes in gene expression, in comparison to other primates [10]. While there is emerging evidence for an important role of small RNAs shaping human-specific brain transcriptomes via posttranscriptional mechanisms [11] and increased recruitment of recently evolved genes during early brain development [12], the role of TSS and other cis-regulatory mechanisms remains unclear. Here, we report that cell type-specific epigenome mapping in prefrontal cortex (PFC, a type of higher order cortex closely associated with the evolution of the primate brain) revealed hundreds of sequences with human-specific H3K4me3 enrichment in neuronal chromatin, as compared to two other anthropoid primates, the chimpanzee and the macaque. These included multiple sites carrying a strong footprint of hominid evolution, including accelerated nucleotide substitution rates specifically in the human branch of the primate tree, regulatory motifs absent in non-human primates and archaic hominins including *Homo neanderthalensis* and *H. denisova*, and evidence for adaptive fixations in modern day humans. The findings presented here provide the first insights into human-specific modifications of the neuronal epigenome, including evidence for coordinated epigenetic regulation of sites separated by megabases of interspersed sequence, which points to a significant intersect between evolutionary changes in TSS function, species-specific chromatin landscapes, and epigenetic inheritance.

## Results

### H3K4me3 Landscapes across Cell Types and Species

The present study focused on the rostral dorsolateral PFC, including cytoarchitectonic Brodmann Area BA10 and the immediately surrounding areas. These brain regions represent a higher association cortex subject to disproportionate morphological expansion during primate evolution [13], and are involved in cognitive operations important for informed choice and creativity [14],[15], among other executive functions. Given that histone methylation in neuronal and non-neuronal chromatin is differentially regulated at thousands of sites genome-wide [7], we avoided chromatin studies in tissue homogenates because glia-to-neuron ratios are 1.4- to 2-fold higher in mature human PFC as compared to chimpanzee and macaque [16]. Instead, we performed cell type-specific epigenome profiling for each of the three primate species, based on NeuN (“neuron nucleus”) antigen-based immunotagging and fluorescence-activated sorting, followed by deep sequencing of H3K4me3-tagged neuronal nucleosomes.

**Figure 1:**
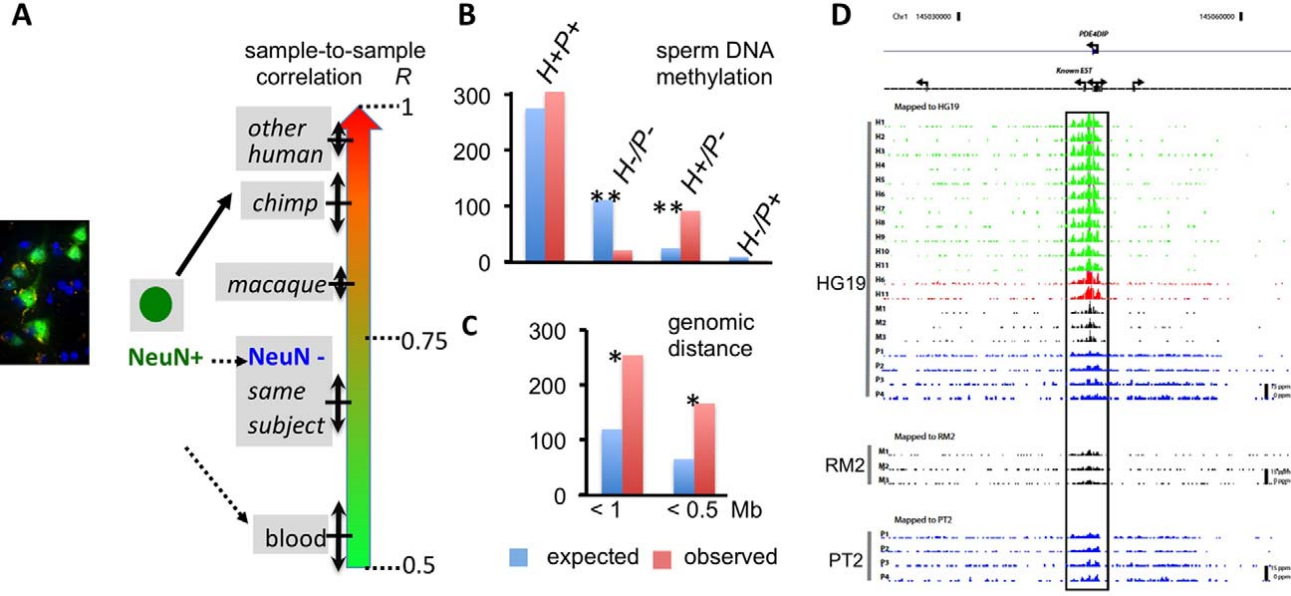
Human-specific signatures of the neuronal epigenome in PFC. (A) Pearson correlation coefficients (*R*, mean ± standard deviation [SD]) for sample-to-sample comparison of H3K4me3 ChIP-seq normalized tag counts within Refseq promoters, revealing cell type- and species-specific signatures. (B) Expected (blue)/observed (red) counts of human-specific H3K4me3 peaks (*n* = 410) overlapping with DNA hypomethylated regions in human (H)/chimpanzee (P) sperm. Notice 4-fold enrichment for loci with human-only (H+,P−) DNA hypomethylation in dataset [19]. (C) The actual co-localization of human-specific H3K4me3 peaks (*n* = 410) within 1- or 0.5-Mb genomic distance is 2–3-fold higher than expected (based on average distribution of entire set of 34,639 H3K4me3 peaks *^(^**^)^, *p*<10^−3(−4)^. (D) Representative example of a TSS (*PDE4DIP/Myelomegalin* (“*regulator of brain size*”) with species- and cell type-specific H3K4me3 profile. Genome browser tracks showing ChIP-seq H3K4me3 signal at *PDE4DIP* (chromosome 1) locus, annotated to HG19/PT2/RM2 genomes as indicated. Green/blue/black tracks from PFC neuronal (NeuN+) nuclei of 11 humans/four chimpanzees/three macaques as indicated. Red tracks, non-neuronal (NeuN−) human PFC nuclei. Notice much stronger PDE4DIP peaks in human neurons.

Prefrontal H3K4me3 epigenomes from NeuN+ nuclei of 11 humans, including seven children and four adults [7], were compared to four chimpanzees and three macaques of mature age (Table S1). Sample-to-sample comparison, based on a subset of highly conserved Refseq TSS with one mismatch maximum/36bp, consistently revealed the highest correlations between neuronal epigenomes from the same species (Table S2). Strikingly, however, the H3K4me3 landscape in human neurons was much more similar to chimpanzee and macaque neurons, when compared to non-neuronal (NeuN−) cells [7] from the same specimen/donor or to blood (Figure 1A). Therefore, PFC neuronal epigenomes, including their histone methylation landscapes at TSS, carry a species-specific signature, but show an even larger difference when compared to their surrounding glial and other NeuN− cells.

### Several Hundred Loci Show Human-Specific Gain, or Loss, of Histone Methylation in PFC Neurons

To identify loci with human-specific H3K4me3 enrichment in PFC neurons, we screened 34,639 H3K4me3 peaks that were at least 500 bp long and showed a consistent >2-fold H3K4me3 increase for the 11 humans as compared to the average of the seven chimps and macaques and (ii) minimum length of 500 bp. We identified 410 peaks in the human genome (HG19) with significant enrichment compared to the two non-human primate species (with reads also mapped to HG19) after correcting for false discovery (FDR), and we call these peaks “HP” hereafter for “human-specific peaks” (Figure 1D; Table S3). We had previously reported that infant and child PFC neurons tend to have stronger peaks at numerous loci, compared to the adult [7]. To better age-match the human and non-human primate cohorts, we therefore repeated the analysis with our entire, recently published cohort of nine adult humans without known neurological or psychiatric disease [7],[8]. Using the same set of filter criteria (>2-fold increase in humans compared to chimpanzees and macaques), we identified 425 peaks and 296 of them overlapped with the original 410 HP (Table S3). Furthermore, 345 of the 410 peaks overlapped with the overlapped with the peaks with >1.5-fold increase for nine adult humans (compared to non-human primates; with correction for FDR) (Table S4), indicating that HPs can be detected reliably.

To obtain human depleted peaks we used a reciprocal approach where initial peaks were detected in chimpanzee and macaque. For the original cohort of 11 children and adult humans, this resulted in 61 peaks with a significant, at least 2-fold depletion in human PFC neurons (Table S5). 50 peaks defined by human-specific depletion in the mixed cohort of 11 children and adults were part of the total of 177 peaks with >1.5-fold decrease in the cohort of nine adults (compared to each of the two non-human primate species; Table S6). From this, we conclude that at least 471 loci in the genome of PFC neurons show robust human-specific changes (gain, 410; loss, 61) in histone methylation across a very wide postnatal age range.

We further explored chimpanzee-specific changes in the H3K4me3 landscape of PFC neurons by comparing human and chimpanzee peaks within the chimpanzee genome. To this end, we constructed a mono-nucleosomal DNA library from chimpanzee PFC to control for input, and mapped the neuronal H3K4me3 datasets from four chimpanzee PFC specimens, and their 11 human counterparts, to the chimpanzee genome (PT2). We identified 551 peaks in the PT2 genome that were subject to >2-fold gain and 337 peaks subject to >2-fold depletion, compared to human regardless of the H3K4me3 level in macaque (Tables S7 and S8). A substantial portion of these PT2-annotated peaks (133 and 40 peaks, respectively) with gain or loss in chimpanzee PFC neurons matched loci with the corresponding, reciprocal changes specific to human PFC neurons in HG19 (410 and 61 peaks as described above). Genetic differences among these genomes and additional, locus-specific differences in nucleosomal organization (leading to differences in background signal in the input libraries) are potential factors that would lead to only partial matching of peaks when species-specific H3K4me3 signals are mapped within the human, or chimpanzee genome, respectively. These findings, taken together, confirm that genome sequence differences in *cis* are one important factor for the species-specific histone methylation landscapes in PFC neurons.

### Human-Specific H3K4me3 Peaks in PFC Neurons Overlap with DNA Methylation Signatures in the Male Germline

Both catalytic and non-catalytic subunits of H3K4 methyltransferase complex are associated with transgenerational epigenetic inheritance in the worm, *Caenorhabditis elegans*, and other simple model organisms [17], and furthermore, H3K4me3 and other epigenetic markings such as DNA cytosine methylation are readily detectable in non-somatic (“germline”-related) cells such as sperm, potentially passing on heritable information to human offspring [18]. Therefore, we wanted to explore whether a subset of the 410 loci with at least 2-fold H3K4me3 enrichment in human neurons are subject to species-specific epigenetic regulation in germ tissue. To this end, we screened a human and chimpanzee sperm database on DNA methylation [19], in order to find out which, if any of the 410 sequences with human-specific H3K4me3 gain in brain overlap with a set of >70,000 sequences defined by very low, or non-detectable DNA methylation in human and chimpanzee sperm (termed (DNA) “hypomethylated regions” in [19]). Of note, the genome-wide distribution of H3K4me3 and DNA cytosine methylation is mutually exclusive in germ and embryonic stem cells, and gains in DNA methylation generally are associated with loss of H3K4me3 in differentiated tissues [20],[21]. Unsurprisingly therefore, 300/410 HP peaks in brain matched a DNA hypomethylated sequence in sperm of both species. Strikingly, however, 90/410, or approximately 22% of HP were selectively (DNA) hypomethylated in human but not in chimpanzee sperm (Table S3), a ratio that is approximately 4-fold higher than the expected 5.7% based on 10,000 simulations (*p*<0.00001; see also Text S1) (Figure 1B). Conversely, the portion of HP lacking DNA hypomethylation in male germ cells of either species altogether (18/410 or 4%), or with selective hypomethylation in chimpanzee sperm (2/410 or 0.5%), showed a significant, 5-fold underrepresentation in our dataset (Figure 1B). Thus, approximately one-quarter of the 410 loci with human-specific gain in histone methylation in PFC neurons also carry species-specific DNA methylation signatures in sperm, with extremely strong bias towards human (DNA) hypomethylated regions (22%) compared to chimpanzee-specific (DNA) hypomethylated regions (0.5%). In striking contrast, fewer than ten of the 61 loci with human-specific H3K4me3 depletion in PFC neurons showed species-specific differences in sperm DNA methylation between species (six human- and three chimpanzee-specific DNA hypomethylated regions; Table S5).

### H3K4 Methylation Sites with Human-Specific Gain Physically Interact in Megabase-Scale Higher Order Chromatin Structures and Provide an Additional Layer for Transcriptional Regulation

We noticed that, at numerous chromosomal loci, HP tended to group in pairs or clusters (Table S3). There were more than 245 (163) from the total of 410 HP spaced less than 1 (or 0.5) Mb apart, which is a highly significant, 2- (or 3-) fold enrichment compared to random distribution within the total pool of 34,639 peaks (Figure 1C; Text S1). Therefore, sequences with human-specific gain in H3K4me3 in PFC neurons appear to be co-regulated with neighboring sequences on the same chromosome that are decorated with the same type of histone modification. Likewise, the actual number of human-depleted peaks within one 1 Mb (*n* = 6) was higher than what is expected from random distribution (*n* = 2.6), (*p* = 0.051), albeit no firm conclusions can be drawn due to the smaller sample size (*n* = 61).

This type of non-random distribution due to pairing or clustering of the majority of human-enriched sequences broadly resonates with the recently introduced concept of Mb-sized topological domains as a pervasive feature of genome organization, including increased physical interactions of sequences carrying the same set of epigenetic decorations within a domain [22]. Of note, H3K4 trimethylation of nucleosomes is linked to the RNA polymerase II transcriptional initiation complex, and sharply increased around TSS and broadly correlated with “open chromatin” and gene expression activity [5],[6]. Therefore, we reasoned that a subset of human-enriched “paired” H3K4me3 peaks could engage in chromatin loopings associated with transcriptional regulation. This is a very plausible hypothesis given that promoters and other regulatory sequences involved in transcriptional regulation are often tethered together in loopings and other higher order chromatin [23],[24].

**Figure 2:**
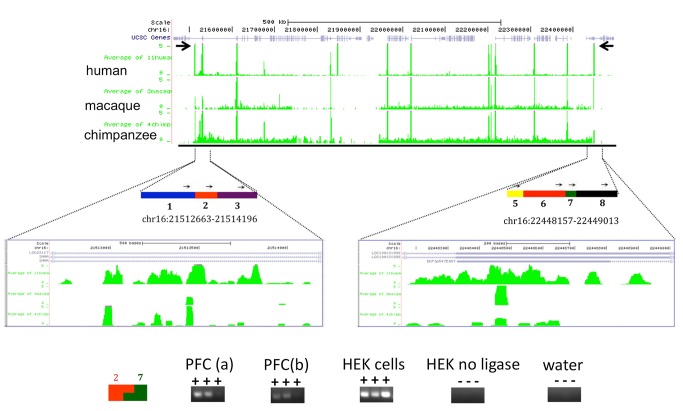
H3K4me3 landscapes and higher order chromatin at the psychiatric susceptibility locus, 16p11.2. (Top) UCSC genome browser window track for approximately 1 Mb of human chr16: 21,462,663–22,499,013, with H3K4me3 ChIP-seq tracks from neuronal chromatin (PFC) of three primate species, as indicated. Notice human-enriched H3K4me3 peaks at chr16:21,512,663–21,514,196 and chr16:22,448,157–22,449,013 (marked by arrows) flanking numerous peaks common to all 3 species. (Bottom) Rectangles and thin arrows mark 3C HindIII restriction fragments and primers from 3C assays. Notice positive interaction of sequences captured by primers 2 and 7, agarose gels shows representative 196-bp PCR product for 3C from two PFC specimens (a,b), HEK cells, and no ligase and water controls.

To explore this, we screened a database obtained on chromatin interaction analysis by paired-end tag sequencing (ChIA-PET) for RNA polymerase II, a technique designed to detect chromosomal loopings bound by the Pol II complex [25]. Indeed, we identified at least three interactions that matched to our H3K4me3 peaks with human-specific gain in PFC neurons (Table S9), including a loop interspersed by approximately 2.5 Mb of sequence in chromosome 16p11.2–12.2. This is a risk locus for microdeletions that are linked to a wide spectrum of neurodevelopmental disease including autism spectrum disorder (ASD), intellectual disability (ID), attention deficit hyperactivity disorder (ADHD), seizures, and schizophrenia [26]–[31]. We were able to validate this interaction by chromosome conformation capture (3C), a technique for mapping long range physical interactions between chromatin segments [32], in 2/2 human PFC specimens and also in a human embryonic kidney (HEK) cell line (Figure 2). We conclude that human-specific H3K4me3 peaks spaced as far apart as 1 Mb are potentially co-regulated and physically interact via chromatin loopings and other higher order chromatin structures.

### Neuronal Antisense RNA LOC389023 Originating from a DPP10 (Chromosome 2q14) Higher Order Chromatin Structure Forms a Stem-Loop and Interacts with Transcriptional Repressors

**Figure 3:**
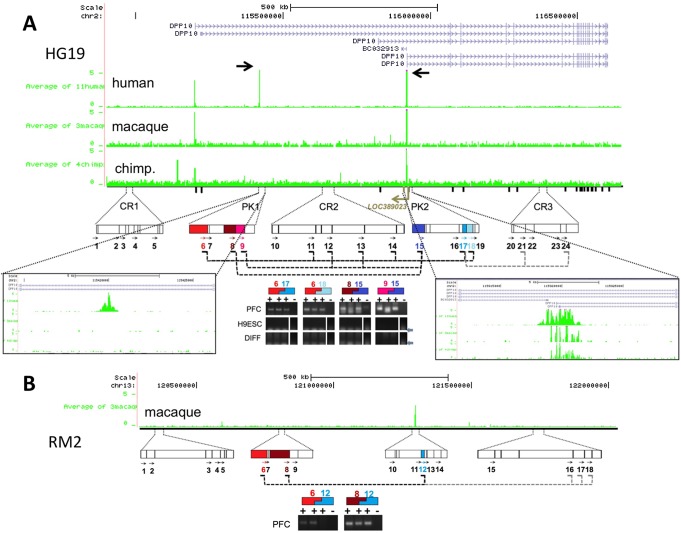
H3K4me3 landscapes and higher order chromatin at *DPP10* (2q14.1). (A) (Top) Genome browser tracks showing ChIP-seq H3K4me3 signal at *DPP10* locus annotated to HG19 and RM2 genomes. Data expressed as normalized tag densities, averaged for 11 humans, four chimpanzees, and three macaques as indicated (see also Figure S1 for comparative annotation for each of the 18 specimens in HG19 at *DPP10*/2q14.1, and for the non-human primates also for the homologous loci in their respective genomes, PT2 and RM2). Human-specific peak *DPP10*-1 (1,455 bp) and *DPP10*-2 (3,808 bp) marked by arrows and shown at higher resolution in boxes, as indicated. (Bottom) Rectangles and arrows mark Hind III restriction fragments and primers from *DPP10*-1/2 (PK1, 2) and control regions (CR1-3) for 3C assays (human). Dotted lines connect primer pairs with sequence-verified product, indicating physical interaction of the corresponding fragments. Agarose gels for representative PCR products from 3C with (+) or without (−) DNA ligase (human primers 6,17: 282 bp; 6,18: 423 bp; 8,15: 160 bp; 9,15: 130 bp). (B) Rectangles and arrows mark Hind III restriction fragments and primers for corresponding DPP10 sequences in RM2, for macaque brain 3C. Macaque primers 6,12:298 bp, 8,12:154 bp. Notice positive interaction of PK1 with PK2 and neighboring CR2, but with not CR1 or CR3. Notice no signal in PFC 3C assays without DNA ligase and no signal in all 3C assays from H9 pluripotent (H9ESC) and differentiated (DIFF) cell cultures.

Next, we wanted to explore whether sequences with human-specific gain in histone methylation, including those that show evidence for pairing and physical interactions, could affect the regulation of gene expression specifically in PFC neurons. To this end, we first identified which portion from the total of 410 human-specific peaks showed much higher H3K4me3 levels selectively in PFC neurons, when compared to their surrounding non-neuronal cells in the PFC. Thus, in addition to the aforementioned filter criteria (2-fold increase in human PFC neurons compared to non-human primate PFC neurons), we searched for peaks with differential regulation among PFC neurons and non-neurons (see Text S1). We found 33 HP with selective enrichment in neuronal PFC chromatin (termed *^neu^*HP in the following) (Figure S1; Table S10). Among these were two HP spaced less than 0.5 Mb apart within the same gene, *DPP10* (chr2q14.1), encoding a dipeptidyl peptidase-related protein regulating potassium channels and neuronal excitability (Figure 3A–3B) [33]. Interestingly, rare structural variants of DPP10 confer strong genetic susceptibility to autism, while some of the gene's more common variants contribute to a significant risk for bipolar disorder, schizophrenia, and asthma [34]–[36]. Histone methylation at *DPP10* was highly regulated in species- and cell type-specific manner, with both *DPP10*-1 and *DPP10*-2 peaks defined by a very strong H3K4me3 signal in human PFC neurons (Figure 3A), but only weak or non-detectable peaks in their surrounding NeuN− (non-neuronal) nuclei (Figure S1; Table S10) or blood-derived epigenomes [7].

We then employed 3C assays across 1.5 Mb of the *DPP10* (chr2q14.1) in PFC of four humans. To increase the specificity in each 3C PCR assay, we positioned both the forward and reverse primer in the same orientation on the sense strand, and samples processed for 3C while omitting the critical DNA ligation step from the protocol served as negative control (Figure 3A–3B). Indeed, 3C assays on four of four human PFC specimens demonstrated direct contacts between the *DPP10*-1 and -2 peaks (Figure 3A). As expected for neighboring fragments [32], *DPP10*-1 also interacted with portions of the interspersed sequence (CR2 in Figure 3A). These interactions were specific, because several other chromatin segments within the same portion of chr2q14.1 did not show longer range interactions with *DPP10*-1 (CR1, CR3 in Figure 3A). We further verified one of the *DPP10-1/2* physical interactions (the sequences captured by primers 6 and 17 in Figure 3A) in four of five brains using 3C-qPCR with a TaqMan probe positioned in fragment 6. Furthermore, *DPP10*-2 interacted with a region (“CR3” in Figure 3A) 400 kb further downstream positioned in close proximity to a blood-specific H3K4me3 peak. No interactions at the *DPP10* locus were observed in cultured cells derived from the H9 embryonic stem cell line (H9ESC in Figure 3A), suggesting that these chromatin architectures are specific for differentiated brain tissue. Of note, similar types of *DPP10* physical interactions were found in 3C assays conducted on PFC tissue of three of three macaques (Figure 3B). Because macaque PFC, in comparison to human, shows much weaker H3K4 methylation at these *DPP10* sequences, we conclude that the corresponding chromatin tetherings are not critically dependent on human-specific H3K4me3 dosage.

**Figure 4:**
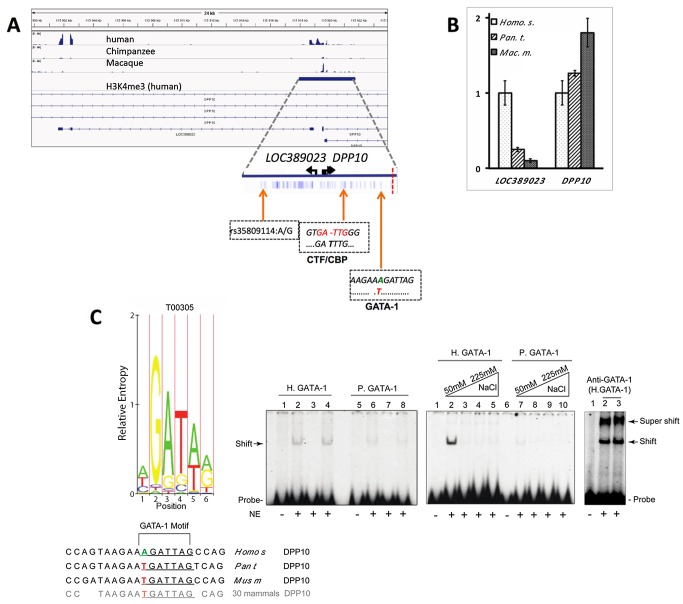
Novel transcripts and regulatory motifs at the DPP10 *locus*. (A) (top) *DPP10* and *LOC389023*, extracted from published RNA-seq datasets from human/chimpanzee/macaque PFC [40]. (Bottom) shows 3.8-kb *DPP10*-2 bidirectional promoter, blue tick marks for human-specific sequence divergence from five other anthropoid primates (Table S12), including (from left to right) SNP rs35809114, and fixed polymorphism with novel CTF/CBP motif not found in archaic hominins (*H. denisova*, *H. neanderthalensis*) and novel GATA-1 motif within highly conserved sequence across many mammalian lineages (Table S17). The vertical dotted red line marks the potential center of an adaptive fixation in modern humans (see text). (B) Bar graphs summarize qRT-PCR on PFC RNA showing much higher *LOC389023* in human, and lower expression of *DPP10* exons downstream of *DPP10*-2 peak (Figure 4A). *^(^**^)^ *p*<0.05 (0.01). (C) (Left) GATA-1 consensus motifs/binding affinities (http://snpper.chip.org/mapper). (Right) HeLa nuclear extract (NE) gel shifts with ^32^P-labeled 21 bp duplex probes for human (H) and chimpanzee (P) sequences encompassing GATA-1 motif as indicated. (Left gel) lanes (1,2,5,6) labeled probe, (3,7) cold competitor, (4,8) unrelated duplex, or increasing salt concentrations as indicated. Anti-GATA supershift assay confirms GATA-1 protein binding to probe sequence.

Next, we wanted to explore whether human-specific H3K4 methylation at the *DPP10* locus is associated with a corresponding change in gene expression at that locus. Notably, H3K4me3 is on a genome-wide scale broadly correlated with transcriptional activity, including negative regulation of RNA expression by generating very short (∼50–200 nt) promoter-associated RNAs. These short transcripts originate at sites of H4K4me3-tagged nucleosomes and act as cis-repressors in conjunction with polycomb and other chromatin remodeling complexes [37],[38]. Therefore, transcriptional activities due to the emergence of novel H3K4me3 markings in human PFC is likely to be complex, with unique functional implications specific to each genomic locus. To explore the transcriptome at the *DPP10* locus in an unbiased manner, we performed RNA-seq on a separate cohort of three adult human PFC (not part of the aforementioned ChIP-seq studies) and compared their transcriptional landscapes to similar datasets from chimpanzee and macaque [39],[40]. Indeed, we found an antisense RNA, *LOC389023*, emerging from the second DPP10 peak, *DPP10*-2 (chr2q14.1) (Figures 3A and 4A). In an additional independent analyses (using a set of human postmortem brains different from the ones used for RNAseq) quantitative reverse transcriptase (RT)-PCR assays further validated the much higher expression of *DPP10* antisense transcript in human (Figure 4B), which occurred in conjunction with decreased expression of *DPP10* exons downstream of the DPP10-2 promoter (compared to chimp/macaque) (Figure 4A).

**Figure 5:**
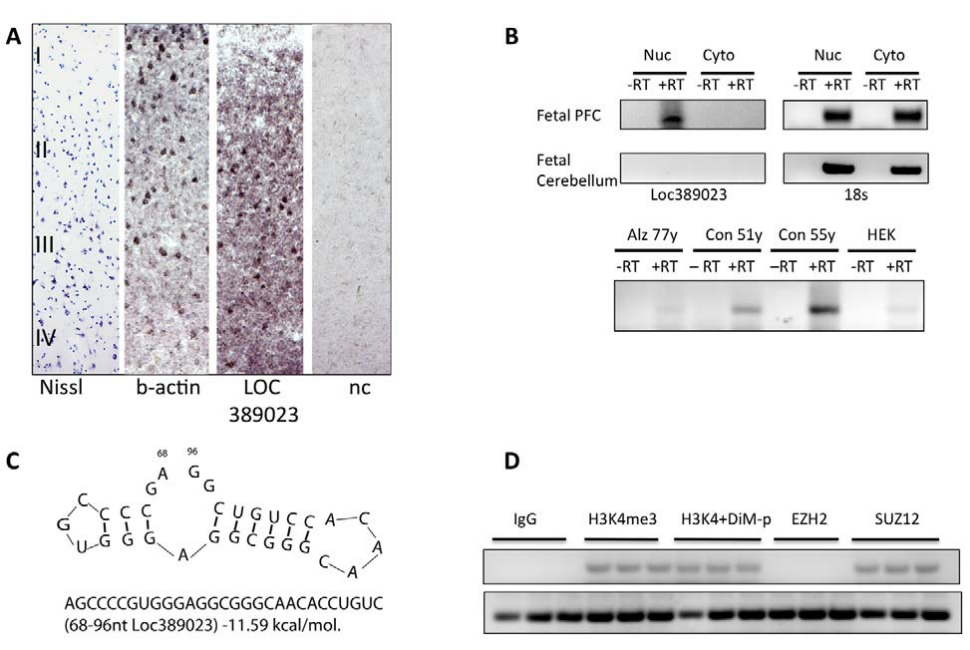
Cellular distribution and molecular affinities of human-specific RNA, *LOC389023*. (A) Digitized images of sections from adult human PFC, stained with (left to right) Nissl, b-actin, LOC389023, and negative control (nc). Notice numerous LOC389023-expressing cells in cortical layers II–IV but not in neuron-poor layer I. (B) (Top) LOC389023, and for loading control, 18S rRNA PCR from nuclear (Nuc) and cytosolic (Cyto) RNA extracts, showing robust LOC389023 expression in nuclear fraction but not cytosolic of a prenatal (around 35 wk of gestation) PFC specimen. No LOC389023 expression was found in fetal cerebellum. (Bottom) PCR from nuclear RNA isolates of adult PFC specimens and of HEK cell line. Notice weak signal in neurodegenerative Alzheimer PFC specimen, no signal in peripheral (HEK) cells, and strong signal in PFC nuclei from normal adult controls. (C) GC rich stem loop of LOC389023 (see text). (D) RT-PCR for LOC389023 from (top) pulldowns of transfected neuroblastoma cells, (left to right) IgG, H3K4-trimethylated nucleosomal preparation co-incubated with or without dimethyl-H3K4-blocking peptide, anti-EZH2, anti-SUZ12, and (bottom) input loading control. Notice specific affinity of LOC389023 for H3K4me3 and SUZ12.

**Figure 6:**
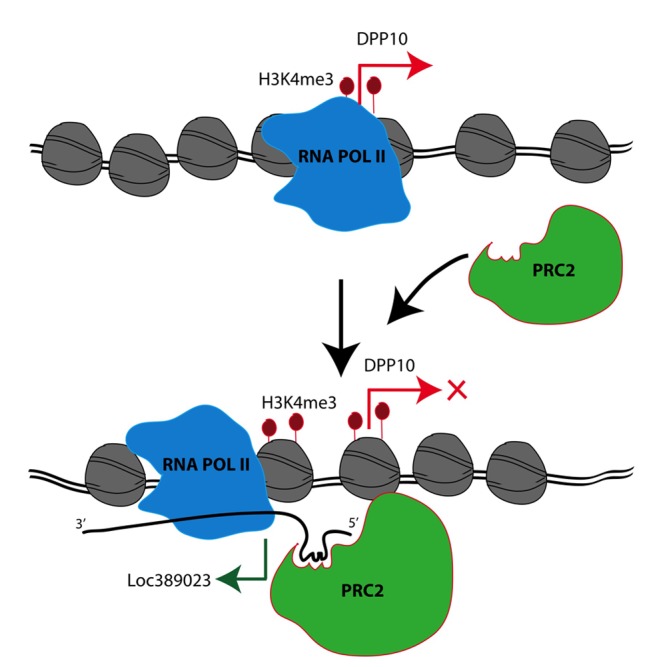
Hypothetical mechanism of action of novel human-specific RNA, LOC389023. (Top) In non-human primate, *DPP10* transcripts are expressed by the RNA polymerase II complex from the *DPP10-2* promoter (see text) that is tagged with H3K4me3. (Bottom) In human, there is specific gain of H3K4me3 signal particularly in the 5′ portion of the DPP10-2 promoter (see text), which is associated with a novel antisense RNA, LOC389023. This RNA recruits Polycomb 2(PRC2) and other transcriptional repressors in *cis*, thereby inhibiting expression of the sense transcript, *DPP10*.

Consistent with the H3K4me3 enrichment specifically in neuronal chromatin, the cellular expression of *LOC389023* in adult PFC was confined to a subset of the neuronal layers (II–IV), but absent in neuron-poor compartments such as layer I and subcortical white matter (Figure 5A and unpublished data). Furthermore, the transcript was expressed in fetal and adult PFC but not in cerebellar cortex (Figure 5B). We noticed that *LOC389023* harbored a GC-rich stem loop motif that is known to associate with cis-regulatory mechanisms involved in transcriptional repression, including binding to TSS chromatin and components of *Polycomb 2* (PRC2) complex (Figure 5C) [37],[41]. Consistent with a possible function inside the nucleus, *LOC389023* was highly enriched in nuclear RNA fractions from extracted prenatal and normal (non-degenerative) adult human PFC, but not cerebellar cortex (Figure 5B). Indeed, in transiently transfected (human) SK-N-MC neuroblastoma cells, *LOC389023* showed a specific association with H3K4-trimethylated nucleosomes and SUZ12 (Figure 5D), a zinc finger protein and core component of PRC2 previously shown to interact with stem loop motifs similar to the one shown in Figure 5C [37]. In contrast, EZH2, a (H3K27) methyltransferase and catalytic component of PRC-2, did not interact with LOC389023 (Figure 5D), consistent with previous reports on other RNA species carrying a similar stem loop motif [37]. These observations, taken together, are entirely consistent with the aforementioned findings that levels of *DPP10* transcript, including exons positioned downstream of the *DPP10*-2 peak from which *LOC389023* originates, are significantly decreased in human PFC as compared to macaque and chimpanzee. Conversely, these two primates show non-detectable (RNAseq) or much lower quantitative RT-PCR (qRT-PCR) *LOC389023* levels in the PFC, as compared to human (Figure 4A–4B). Taken together then, these findings strongly suggest that *LOC389023* emerged de novo in human PFC neurons and interacts with localized chromatin templates to mediate transcriptional repression at the *DPP10* locus (Figure 6).

### Association of Human-Specific H3K4-Methylation Sites with Disease

The aforementioned human-specific gains in histone methylation at *DPP10* and the emergence of human RNA de novo at this locus could reflect a phylogenetically driven reorganization of neuronal functions that may have contributed not only to the emergence of human-specific executive and social-emotional functions, but also for increased susceptibility for developmental brain disease [42]. In this context, we noticed that the 33 *^neu^*HP (which are defined by two criteria which are (i) human-specific gain compared to non-human primates and (ii) high H3K4me3 in PFC neurons but not their surrounding non-neuronal cells) included multiple genes conferring susceptibility to neurological disease. Three loci, including *DPP10* on chromosome 2q14.1 and two genes in close proximity on chromosome 3p26.3, *CNTN4* and *CHL1*, both encoding cell adhesion molecules [34],[43]–[45], confer very strong susceptibility to autism, schizophrenia, and related disease. Other disease-associated loci with human-specific gain selectively in PFC neurons include *ADCYAP1*, a schizophrenia [46],[47] and movement disorder gene [48] that is part of a cAMP-activating pathway also implicated in posttraumatic stress [49]. *PDE4DIP* (*MYOMEGALIN*) (Figure 1D) encodes a centrosomal regulator of brain size and neurogenesis [50] that in some studies was 9-fold higher expressed in human as compared to chimpanzee cortex [51],[52]. *SORCS1* is implicated in beta amyloid processing and Alzheimer disease [53],[54] and attention deficit hyperactivity disorder [55], which again are considered human-specific neurological conditions [10]. Because four of 33, or 12% of *^neu^*HP overlapped with neurodevelopmental susceptibility genes (*CNTN4, CHL1, DPP10, SORCS1*), we then checked whether the entire set of 410 human-specific peaks is enriched for genes and loci conferring genetic risk for autism, intellectual disability, and related neurological disease with onset in early childhood. However, there was only minimal overlap with the Simons Foundation Autism Research Initiative database (SFARI) [56], and Human unidentified Gene Encoded protein database (HuGE) for pervasive developmental disorder (including autism) associated polymorphism [57], and recent reference lists for mental retardation and/or autism-related genes (each of these databases five or fewer of the human-enriched peaks) [58]. Likewise, there was minimal, and non-significant overlap with the set of 61 human- and 337 chimpanzee-depleted peaks, or the 551 chimpanzee-enriched in PFC neurons (five or fewer of peaks/database). None of the lists of peaks with human- or chimpanzee-specific gain or loss of H3K4me3 revealed statistical significance for any associations with the Gene Ontology (GO) database. We conclude that DNA sequences subject to differential histone methylation in human or chimpanzee PFC neurons are, as a group, not clustered together into specific cellular signaling pathways or functions. Table 1 presents examples of disease-associated genes associated with human-specific gain, or loss of H3K4-trimethylation.

**Table 1:** Examples of disease-associated genes with human-specific gain or loss of H3K4 trimethylation in PFC neurons.

Gene; Location; HGNC	Gene	H3K4me3 Change in Human	Disease Association	Function in the Forebrain, Including Cerebral Cortex
*ADCYAP1*; 18p11.32; 241	adenylate cyclase activating polypeptide 1	Gain	Schizophrenia [46],[47], movement disorder [48], PTSD [49]	Alternate camp signaling pathway, mediates synaptic plasticity and LTD in hippocampus [95]
*CACNA1C*; 12p13.33; 1390	calcium channel, voltage-dependent, L type, alpha 1C subunit	Gain	Confers genetic risk for mood, psychosis, and autism spectrum disorders [96],[97]	Coupling of cell membrane depolarization to transient increase of membrane permeability for calcium [96]
*CHL1*; 3p26.3; *1939*	cell adhesion molecule with homology to L1CAM	Gain	Autism, schizophrenia [35],[44]–[46]	Thalamocortical axon guidance via interaction with ephrin receptors [98],[99]
*CNTN4*; 3p26.3; *2174*	contactin 4	Gain	Autism, intellectual disability [34],[43]–[45]	Developmental patterning of functional odor maps in olfactory bulb, axon-associated cell adhesion molecule [34],[43]–[45]
DGCR6; 22q11.21; 2844	DiGeorge syndrome critical region gene 6	Gain	Autism, schizophrenia [74],[75]	Regulates intracellular distribution of GABA_B_ receptor [100]
*DPP10*; 2q14.1; 20823	dipeptidyl-peptidase 10	Gain	Autism, mood disorder, schizophrenia, asthma [34]–[36]	Regulation of neuronal excitability as auxiliary subunit of potassium channels [33]
*FOXP2*; 7q31.1; 13875	forkhead box P2	Loss	Speech and language disorder with subtle structural and functional changes in brain circuitry [81],[82]	Transcription factor regulating gene expression programs in vocal communication, including human speech and birdsong [82],[101],[102]
*LMX1B*; 9q33.3; 6654	Lim homeobox transcription factor 1, beta	Loss	ADHD and depression [103]	Key control point in gene expression programs for dopaminergic and serotonergic neurons [104],[105]
*NOTCH4*; 6p21.3; 7884	neurogenic locus notch homolog gene 4	Gain	Schizophrenia [106],[107]	Endothelial Notch 4 regulates brain vasculature [108]
*PDE4DIP*; 1q21.1; 15580	phoshodiesterase 4D Interacting protein	Gain	Altered phospho-diesterase signaling broadly relevant for mood and psychosis spectrum disorders [109],[110]	Anchor protein for cAMP pathway in the Golgi/centrosomal complex, homologue to drosophila *centrosomin* regulating brain development and implicated in neurogenesis [50],[111]
*SLC2A3*; 12p13.31; 11007	solute carrier family 2 (facilitated glucose transporter),member 3	Gain	Dyslexia, ADHD [78],[79]	Neuronal glucose transporter, highly expressed in neuronal processes and synaptic structures and neuropil of human cerebral cortex and other brain regions [112],[113]
*SORCS1*; 10q25.1; 16697	sortilin-related VPS10 domain containing receptor 1	Gain	ADHD [54]	In a complex with pro-NGF, involved in NGF-mediated cell signaling and neuroapoptosis [114]. Interacts, like other sortilins, with gamma-secretase implicated in Alzheimer disease [54]
*TRIB3*; 20p13; 16228	tribbles homolog 3 pseudo-kinase	Gain	Genetic determinant for information-processing speed in human [115] and insulin-dependent diabetes [116]	Competes in complex with ATF4 with CREB transcription factor to regulate expression of synaptosomal-associated protein 25 (SNAP-25) involved in insulin exocytosis and neurotransmission [116]
*TUBB2B*; 6p25.2; 30829	class IIb beta-tubulin	Gain	Cortical malformations including poly-microgyria [117], microcephaly, seizures, intellectual disability [118]	Essential for neuronal migration and other functions of the microtubuli complex [80]
*ZNF423*; 16p12.1; 16762	zinc-finger protein 423	Loss	16q12 microdeletion syndrome with micro-cephaly and dysmorpho-genesis of fore- and hindbrain [119],[120]	C2H2-type zinc finger transcription factor that controls the switch to neuronal maturation during olfactory neurogenesis [121] and axonal projections across forebrain commissures [122].

ADHD, attention deficit hyperactivity disorder; LTD, long-term depression; NGF, nerve growth factor; PTSD, post-traumatic stress disorder.

### Evolutionary Footprints at Sites Defined by Human-Specific Histone Methylation

We then asked whether the subset of DNA sequences with species- and cell type-specific epigenetic regulation, including the *^neu^*HP peaks mentioned above carry a strong footprint of hominid evolution. Indeed, nucleotide substitution analysis revealed that both *DPP10 peaks DPP10 -1/2*, as well as *ADCYAP1*, *CHL1*, *CNTN4*, *NRSN2*, and *SIRPA* show a significantly elevated rate, with 2- to 5-fold increase specifically in the human branch of the primate tree, when compared to four other anthropoid primate species (*Pan troglodytes, Gorilla gorilla*, *Pongo abelii, Macaca mulatta*) (Table S11). The finding that both *DPP10* peaks, *DPP10*-1 and -2 showed a significant, >4-fold increase in nucleotide substitution rates in the human branch of the primate tree—indicating “co-evolution” (or coordinated loss of constraint)—is very plausible given that chromatin structures surrounding these DNA sequences are in direct physical contact (discussed above), reflecting a potential functional interaction and shared regulatory mechanisms between peaks.

To further confirm the role of phylogenetic factors in the emergence of human-specific H3K4me3 peaks, we focused on the set of 33 *^neu^*HP and calculated the total number of human-specific sequence alterations (HSAs), in a comparative genome analyses across five primates (*H. sapiens*, *P. troglodytes, G. gorilla*, *P. abelii, M. mulatta*). We recorded altogether 1,519 HSAs, with >90% as single nucleotide substitutions, five >100 bp INDELs, one (*Alu*) retrotransposon-like element at *TRIB3* pseudokinase consistent with a role of mobile elements in primate evolution [3], and gain or loss of hundreds of regulatory motifs (Table S12). When compared to a group of (neuronal) H3K4me3 peaks showing minimal changes between the three primate species (Table S13), the ^neu^HP, as a group, showed a significant, 2.5-fold increase in the number of HSA (20.08±5.52 HSAs versus 8.36±2.44 HSAs per 1-kb sequence, *p* = 2.4e−06, Wilcoxon rank sum test; Figure S3). The findings further confirm that genetic differences related to speciation indeed could play a major role for changes in the brain's histone methylation landscape, particularly for H3K4me3 peaks that are highly specific for human neurons (^neu^HP). Interestingly, none of the above loci showed evidence for accelerated evolution of neighboring protein coding sequences (Table S11), reaffirming the view that protein coding sequences for synaptic and other neuron-specific genes are extremely conserved across the primate tree [1],[2].

These DNA sequence alterations at sites of neuron-restricted H3K4me3 peaks (with human-specific gain) point, at least for this subset of loci, to a strong evolutionary footprint before the split of human–chimpanzee lineage several million years ago [3]. Next, we wanted to find out whether there is also evidence for more recent selective pressures at these loci. Indeed, a subset of *^neu^*HP contain *H. sapiens*-specific sequences not only absent in rodents, anthropoid primates, but even in extinct members of the genus *homo*, including *H. neanderthalensis* and *H. denisova* [59]. Some of the ancestral alleles (including *MIAT, SIRPA, NRSN*) shared with archaic hominins exhibit very low frequencies at 0%–3% in all modern populations, and therefore it remains possible that positive selection for newly derived alleles contributed to their high population frequencies in modern humans (Table S14). However, for the entire set of *^neu^*HP that are defined by high H3K4me3 levels in PFC neurons (but not non-neurons), the number of HSAs that emerged after the human lineage was split from *H. denisova* or *H. neanderthalensis* were 3.31% and 1.75%, respectively, which is approximately 2-fold lower as compared to 32 control H3K4me3 peaks with minimal differences among the three primate species (5.03% and 3.77%). The 2-fold difference in the number of *H. sapiens*-specific alleles (*^neu^*HP compared to control peaks) showed a strong trend toward significant (*p* = 0.067) for the Denisova, and reached the level of significance (*p* = 0.034) for the Neanderthal genome (based on permutation test with 10,000 simulations [60]). Taken together, these results suggest that at least a subset of the TSS regions with H3K4me3 enrichment in human (compared to non-human primates) were exposed to evolutionary driven DNA sequence changes on a lineage of the common ancestor of *H. sapiens* and the archaic hominins, but subsequently were stabilized in more recent human evolution, after splitting from other hominins.

To further test whether or not there were recent, perhaps even ongoing selective pressures at loci defined by human-specific gain in H3K4me3 peaks of PFC neurons, we searched for overlap among the peaks in our study with hundreds of candidate regions in the human genome showing evidence of selection during the past 10–100,000 years from other studies. These loci typically extend over several kb, and were identified in several recent studies on the basis of criteria associated with a “selective sweep,” which describes the elimination of genetic variation in sequences surrounding an advantageous mutation while it becomes fixed [61]–[64]. However, screening of the entire set of 410 human gain and 61 human depleted H3K4me3 sequences against nine datasets for putative selection in humans [65] revealed only five loci with evidence for recent sweeps (Table S15). One of these matched to the *^neu^*HP on chromosome 2q14.1, corresponding to the second *DPP10* (*DPP10*-2) peak (see above). In independent analyses, using the 1,000 genome database, we further confirmed recent adaptive fixations around *DPP10-2* (Table S16), as well as two other loci, *POLL and TSPAN4*. While it is presently extremely difficult to determine how much of the genome has been affected by positive selection (of note, a recent metanalysis of 21 recent studies using total genomic scans for positive selection using human polymorphism data revealed unexpectedly minimal overlap between studies [65]), we conclude that the overwhelming majority of loci associated with human-specific H3K4me3 gain or loss in PFC neurons (compared to non-human primates) indeed does not show evidence for more recent selective pressures.

To provide an example on altered chromatin function due to an alteration in a regulatory DNA sequence that occurred after the human lineage split from the common ancestor with non-human primates, we focused on a change in a GATA-1 motif (A/TGATTAG) within a portion of *DPP10*-2 found in human, within an otherwise deeply conserved sequence across many mammalian lineages (Table S17). Gel shift assays demonstrate that the human-specific sequence harboring the novel GATA-1 site showed much higher affinity to HeLa nuclear protein extracts, compared to the chimpanzee/other mammal sequence (Figure 4C). The emergence of a novel GATA-1 motif at *DPP10* is unlikely to reflect a systemic trend because the motif overall was lost, rather than gained in ^neu^HP (10/355 versus 4/375, χ^2^ *p* = 0.053). Therefore, evolutionary and highly specific changes in a small subset of regulatory motifs at *DPP10* and other loci could potentially result in profound changes in nuclear protein binding at TSS and other regulatory sequences, thereby affecting histone methylation and epigenetic control of gene expression in humans, compared to other mammals including monkeys and great apes. Of note, potentially important changes in chromatin structure and function due to human-specific sequence alterations at a single nucleotide within an otherwise highly conserved mammalian sequence will be difficult to “capture” by comparative genome analyses alone. For example, when the total set of 410 HP was crosschecked against a database of 202 sequences with evidence for human-specific accelerated evolution in loci that are highly conserved between rodent and primate lineages [66], only one of 410 HP matched (Table S15).

### Species-Specific Transcriptional Regulation

H3K4me3 is a transcriptional mark that on a genome-wide scale is broadly associated with RNA polymerase II occupancies and RNA expression [67]. However, it is also associated with repressive chromatin remodeling complexes and at some loci the mark is linked to short antisense RNAs originating from bidirectional promoters, in conjunction with negative regulation of the (sense) gene transcript [37],[38]. Indeed, this is what we observed for the *DPP10* locus (Figure 6). Therefore, a comprehensive assessment of all transcriptional changes associated with the evolutionary alterations in H3K4me3 landscape of PFC neurons would require deep sequencing of intra- and extranuclear RNA, to ensure full capture of short RNAs and all other transcripts that lack polyadenylation and/or export into cytoplasm. While this is beyond the scope of the present study, we found several additional examples for altered RNA expression at the site of human-specific H3K4me3 change. There were four of 33 ^neu^HP loci associated with novel RNA expression specific for human PFC, including the aforementioned *DPP10* locus. The remaining three human-specific transcripts included two additional putative non-coding RNAs, *LOC421321*(chr7p14.3) and *AX746692* (chr17p11.2). There was also a novel transcript for *ASPARATE DEHYDROGENASE ISOFORM 2* (*ASPDH*)(chr19q13.33) (Figure S2). Furthermore, a fifth ^neu^HP, positioned within an intronic portion of the tetraspanin gene *TSPAN4* (chr11p15.5), was associated with a dramatic, human-specific decrease of local transcript, including the surrounding exons (Figure S2). Comparative analyses of prefrontal RNA-seq signals for the entire set of the 410 HP included at least 18 loci showing a highly consistent, at least 2-fold increase or decrease in RNA levels of human PFC, compared to the other two primate species (Table S18).

## Discussion

In the present study, we report that on a genome-wide scale, 471 loci show a robust, human-specific change in H3K4me3 levels at TSS and related regulatory sequences in neuronal chromatin from PFC, in comparison to the chimpanzee and macaque. Among the 410 sequences with human-specific gain in histone methylation, there was a 4-fold overrepresentation of loci subject to species-specific DNA methylation in sperm [19]. This would suggest that there is already considerable “epigenetic distance” between the germline of *H. sapiens* and non-human primates (including the great apes), which during embryonic development and tissue differentiation is then “carried over” into the brain's epigenome. The fact that many loci show species-specific epigenetic signatures both in sperm [19] and PFC neurons (Figure 1B) raises questions about the role of epigenetic inheritance [68] during hominid evolution. However, to further clarify this issue, additional comparative analysis of epigenetic markings in brain and germline will be necessary, including histone methylation maps from oocytes, which currently do not exist. However, the majority of species-specific epigenetic decorations, including those that could be vertically transmitted through the germline, could ultimately be driven by genetic differences. On the basis of DNA methylation analyses in three-generation pedigrees, more than 92% of the differences in methylcytosine load between alleles are explained by haplotype, suggesting a dominant role of genetic variation in the establishment of epigenetic markings, as opposed to environmental influences [69]. A broad overall correlation between genetic and epigenetic differences was also reported in a recent human–chimpanzee sperm DNA methylation study [19], and there is general consensus that the inherent mutability of methylated cytosine residues due to their spontaneous deamination to thymine is one factor contributing to sequence divergence at CpG rich promoters with differential DNA methylation between species [19],[70]. Furthermore, human-specific sequences in the DNA binding domains of *PRDM9*, which encodes a rapidly evolving methyltransferase regulating H3K4me3 in germ cells, were recently identified as a major driver for human–chimpanzee differences in meiotic recombination and genome organization [71]. It will be interesting to explore whether PRDM9-dependent histone methyltransferase activity was involved in the epigenetic regulation of the human-enriched H3K4me3 peaks that were identified in the present study.

Another interesting finding that arose from the present study concerns the non-random distribution of histone methylation peaks with human-specific gain, due to a significant, 2- to 3-fold overrepresentation of peak-pairing or -clustering on a 500 kb to 1 Mb scale. This result fits well with the emerging insights into the spatial organization of interphase chromosomes, including the “loopings,” “‘tetherings” and “globules” that bring DNA sequences that are spatially separated on the linear genome into close physical contact with each other [72]. Specifically, many chromosomal areas are partitioned into Mb-scale “topological domains”, which are defined by robust physical interaction of intra-domain sequences carrying the same set of epigenetic decorations [22]. These mechanisms could indeed have set the stage for coordinated genetic and epigenetic changes during the course of hominid brain evolution. The *DPP10* (2q14.1) neurodevelopmental susceptibility locus provides a particularly illustrative example: here, two H3K4me3 peak sequences with strong human-specific gain were separated by hundreds of kilobases of interspersed sequence, yet showed a strikingly similar, 4-fold acceleration of nucleotide substitution rates specifically in the human branch of the primate tree. Importantly, the two H3K4me3 peaks, *DPP10-1* and -*2*, as shown here, are bundled together in a loop or other types of higher order chromatin. Therefore, our findings lead to a complex picture of the human-specific shapings of the neuronal epigenome, including a mutual interrelation of DNA sequence alterations and epigenetic adaptations involving histone methylation and higher order chromatin structures. The confluence of these factors could then, in a subset of PFC neurons (Figure 5A), result in the expression of a novel antisense RNA, which associates with transcriptional repressors to regulate the target transcript in cis, *DPP10* (Figures 5D and 6).

While the present study identified a few loci, including the aforementioned *DPP10* (chromosome 2q14.1), in which DNA sequences associated with a human-specific gain in neuronal histone methylation showed signs for positive selection in the human population, it must be emphasized that the overwhelming majority of sites with human-specific H3K4me3 changes did not show evidence for recent adaptive fixations in the surrounding DNA. Therefore, and perhaps not unsurprisingly, neuronal histone methylation mapping in human, chimpanzee, and macaque primarily reveals information about changes in epigenetic decoration of regulatory sequences in the hominid genome after our lineage split from the common ancestor shared with present-day non-human primates.

Moreover, according to the present study, the subset of 33 sequences with human-specific H3K4me3 gain and selective enrichment in neuronal (as opposed to non-neuronal) PFC chromatin show a significant, 3-fold increase in human-specific (DNA sequence) alterations in comparison to non-human primate genomes. This finding speaks to the importance of evolutionary changes in regulatory sequences important for neuronal functions. Strikingly, however, the same set of sequences show a significant, approximately 1.5- to 2-fold decrease in sequence alterations when compared to the two archaic hominin (*H. denisova, H. neanderthalensis*) genomes. This finding further reaffirms that sequences defined by differential epigenetic regulation in human and non-human primate brain, as a group, are unlikely to be of major importance for more recent evolution, including any (yet elusive) genetic alterations that may underlie the suspected differences in human and neanderthal brain development [73]. However, these general conclusions by no means rule out a critical role for a subset of human-specific sequence alterations on the single nucleotide level within any of the HPs described here, including the *DPP10* locus.

Such types of single nucleotide alterations and polymorphisms may be of particular importance at the small number of loci with human-specific H3K4me3 gain that contribute to susceptibility of neurological and psychiatric disorders that are unique to human (though it should be noticed that as a group, the entire set of sequences subject to human-specific gain, or loss, of H3K4me3 are not significantly enriched for neurodevelopmental disease genes). The list would not only include the already discussed *ADYCAP1*, *CHL1*, *CNTN4*, and *DPP10*, which were among the narrow list of 33 human-specific peaks highly enriched in neuronal but not non-neuronal PFC chromatin), but also *DGCR6*, an autism and schizophrenia susceptibility gene [74],[75] within the DiGeorge/Velocardiofacial syndrome/22q11 risk locus, *NOTCH4* and *CACNA1C* encoding transmembrane signaling proteins linked to schizophrenia and bipolar disorder in multiple genome-wide association studies [76],[77], *SLC2A3* encoding a neuronal glucose transporter linked to dyslexia and attention-deficit hyperactivity disorder [78],[79] and the neuronal migration gene *TUBB2B* that has been linked to polymicrogria and defective neurodevelopment [80]. Furthermore, among the 61 peaks with human-specific loss of H3K4me3 is a 700-bp sequence upstream of the TSS of *FOXP2*, encoding a forkhead transcription factor essential for proper human speech and language capabilities [81] and that has been subject to accelerated evolution with amino acid changes leading to partially different molecular functions in human compared to great apes [82],[83]. The homebox gene *LMX1B* is another interesting disease-associated gene that is subject to human-specific H3K4me3 depletion (Table 1). While expression of many of these disease-associated genes is readily detectable even in mouse cerebral cortex [84], the neuropsychiatric conditions associated with them lack a correlate in anthropoid primates and other animals. This could speak to the functional significance of H3K4 methylation as an additional layer for transcriptional regulation, with adaptive H3K4me3 changes at select loci and TSS potentially resulting in improved cognition while at the same time in the context of genetic or environmental risk factors contribute to neuropsychiatric disease. More generally, our findings are in line with a potential role for epigenetic (dys)regulation in the pathophysiology of a wide range of neurological and psychiatric disorders [85]–[88].

Our study also faces important limitations. While we used child and adult brains for cross-species comparisons, human-specific signatures in the cortical transcriptome are thought to be even more pronounced during pre- and perinatal development [89]. Therefore, younger brains could show changes at additional loci, or more pronounced alterations at the TSS of some genes identified in the present study, including the above mentioned susceptibility genes *CNTN4* and myelomegalin/*PDE4DIP*, which are expressed at very high levels in the human frontal lobe at midgestation [90]. In this context, our finding that a large majority, or 345 of 410 H3K4me3 peaks showed a human-specific gain both in children and adults, resonates with Somel and colleagues [11] who suggested that some of the age-sensitive differences in cortical gene expression among primate species are due to trans-acting factors such as microRNA,s while *cis*-regulatory changes (which were the focus of the present study) primarily affect genes that are subject to a lesser regulation by developmental processes. More broadly, our studies supports the general view that transcriptional regulation of both of coding and non-coding (including antisense) RNAs could play a role in the evolution of the primate brain [91].

Furthermore, the cell type-specific, neuronal versus non-neuronal chromatin studies as presented here provide a significant advancement over conventional approaches utilizing tissue homogenate. However, pending further technological advances, it will be interesting to explore genome organization in select subsets of nerve cells that bear particularly strong footprints of adaptation, such as the Von Economo neurons, a type of cortical projection neuron highly specific for the hominid lineage of the primate tree and other mammals with complex social and cognitive-emotional skill sets [92]. Furthermore, our focus on PFC does not exclude the possibility that other cortical regions [93], or specialized sublayers such as within the fourth layer of visual cortex that shows a complex transcriptional architecture [94], show human-specific histone methylation gains at additional TSS that were missed by the present study.

More broadly, the approach provided here, which is region- and cell type-specific epigenome mapping in multiple primate species, highlights the potential of epigenetic markings to identify regulatory non-coding sequences with a potential role in the context of hominid brain evolution and the shaping of human-specific brain functions. Remarkably, a small subset of loci, including the aforementioned *DPP10* (chromosome 2q14.1), shows evidence for ongoing selective pressures in humans, resulting in DNA sequence alterations and the remodeling of local histone methylation landscapes, after the last common ancestor of human and non-human primates.

## Materials and Methods

Text S1 contains detailed description for sample preparation for ChIP-seq and RNA-seq, qRT-PCR, gel shift, and 3C assays including primer sequences, RNA immunoprecipitation and in situ hybridization, bioinformatics and analyses of deep sequencing data, exploration of regulatory motifs, calculation of nucleotide substitution rates in the primate tree, and sweep analyses for polymorphic regions.

## Supporting Information

Figure S1 H3K4me3 ChIP-seq browser tracks (UCSC) for ^neu^HP peaks with >2-fold gain in human PFC, compared to chimpanzee and macaque. y-Axis represents normalized tag densities (0–15, ppm) after annotation to the three reference genomes (HG19, rheMac2 = RM2, panTro2 = PT2).
(PDF)

Figure S2 RNAseq tag densities in human, chimpanzee, and macaque PFC for H3K4me3 peaks shown in Figure S1.
(PDF)

Figure S3 Comparison of number of human-specific DNA sequence alterations in H3K4me3 peaks with and without human-specific gain.
(PPTX)

Table S1 Sample information, including age, gender, and postmortem brain interval, and H3K4me3 ChIP-seq parameters.
(XLS)

Table S2 Sample-to-sample correlations of raw promoter tag counts of H3K4me3 ChIP-seq from PFC NeuN+ nuclei.
(XLSX)

Table S3 List of 410 H3K4me3 peaks with human-specific gain, with at least 2-fold higher normalized tag densities in 11 humans as compared to the three macaques and four chimpanzees, including human genome (HG) 19 coordinates, distance to nearest TSS, and overlap (1) or no overlap (0) with DNA hypomethylated regions in sperm DNA methylation database comparing human and chimpanzee [20].
(XLSX)

Table S4 List of 885 H3K4me3 peaks with human-specific gain, with at least 1.5-fold higher tag density in nine adult humans as compared to the three macaques and four chimpanzees.
(XLSX)

Table S5 List of 61 H3K4me3 peaks with human-specific depletion, with at least 2-fold lower normalized tag densities in of 11 (seven children, four adult) humans as compared to the three macaques and four chimpanzees, including human genome (HG) 19 coordinates, distance to nearest TSS, and overlap (1) or no overlap (0) with DNA hypomethylated regions in sperm DNA methylation database comparing human and chimpanzee [20].
(XLSX)

Table S6 List of 177 H3K4me3 peaks with human-specific depletion, with at least 1.5-fold lower normalized tag densities in nine adult humans as compared to the three macaques and four chimpanzees.
(XLSX)

Table S7 List of 551 H3K4me3 peaks with chimpanzee-specific enrichment, with at least 2-fold higher tag density in four chimpanzees compared to 11 humans.
(XLSX)

Table S8 List of 337 H3K4me3 peaks selectively depleted in the chimpanzee, with at least 2-fold higher tag density in 11 humans compared to four chimpanzees.
(XLSX)

Table S9 Sequences with human-specific H3K4me3 gain in prefrontal neurons that were recently shown to be a part of chromatin loopings in conjunction with RNA polymerase II occupancy [25].
(XLSX)

Table S10 Genome coordinates of the subset of 33 ^neu^HP that are significantly enriched in prefrontal neurons (NeuN+) as compared to lymphocytes (see Text S1, “ChiP-seq analyses”) and to non-neurons in the PFC (NeuN−; Figure S1) and that show human-specific gain in 11 humans as compared to the three macaques and four chimpanzees, including HG19 (and after liftover), *Rhesus macaqua* 2 (RM2) and *P. troglodytes 2* (PT2) genome coordinates, and species-specific enrichments.
(XLS)

Table S11 Nucleotide substitution rates for the 33 ^neu^HP defined by human-specific gain and neuron-specific enrichment. Baseml (for genomic sequence) and codeml (for amino acid sequence) was to calculate branch- and site-specific nucleotide substitution rates, using human, chimpanzee, gorilla, orangutan, and macaque genome sequences.
(XLSX)

Table S12 Regulatory motifs (cis-Red database) gained and lost in the 1,519 human-specific DNA sequence alterations found in the 33 ^neu^HP peaks, in comparison to four other primates and in comparison to archaic hominin genomes (*H. neanderthalensis* and *H. denisova*).
(XLSX)

Table S13 List of 32 control peaks with the least/no differences in H3K4me3 levels between human and non-human primates, to determine human-specific sequence alterations and compare with 33 ^neu^HP.
(XLSX)

Table S14 Ancestral allele frequencies for subset of ^neu^HP peaks (Pilot 1000 Genome Project).
(XLSX)

Table S15 Overlap between HP and sequences subject to adaptive fixations (sweep) in modern populations. Overlap between HP and human accelerated sequences within domains highly conserved between rodent and primates.
(XLSX)

Table S16 Polymorphism-based sweep analyses for ^neu^HP peaks, using 1,000 genomes pilot data.
(XLSX)

Table S17 Novel human-specific GATA-1 motif in DPP10-2 promoter sequence otherwise deeply conserved across mammalian lineages.
(XLSX)

Table S18 RNAseq normalized tag densities from human, chimpanzee, and macaque PFC, for HP sequences and their surrounding 1–2 kb.
(XLSX)

Text S1 Methods.
(DOC)

## Abbreviations

3C: chromosome conformation capture
H3K4me3: trimethyl-H3-lysine 4
HEK: human embryonic kidney
HP: human-specific peak
HSA: human-specific sequence alteration
PFC: prefrontal cortex
RT: reverse transcriptase
TSS: transcription start site
