# Supplementary material for: Human-Specific Histone Methylation Signatures at Transcription Start Sites in Prefrontal Neurons
Source: PLoS Biol. 2012 Nov 20;10(11):e1001427. doi: 10.1371/journal.pbio.1001427 (PMC3502543; doi:10.1371/journal.pbio.1001427)
Supplement: Figure S1 — H3K4me3 ChIP-seq browser tracks (UCSC) for neuHP peaks with >2-fold gain in human PFC, compared to chimpanzee and macaque. y-Axis represents normalized tag densities (0–15, ppm) after annotation to the three reference genomes (HG19, rheMac2 = RM2, panTro2 = PT2). (PDF) [file pbio.1001427.s001.pdf]

Supplementary Figure 1 (Shulha et al):

UCSC genome browser tracks showing for each human-specific H3K4me3 peak the tag densities (y-axis, range 0-15 parts per million) for 11 human NeuN+ (green), 2 human NeuN- (red), 3 macaque NeuN+ (black) and 4 chimpanzee NeuN+ (blue) samples; non-human samples mapped both to HG19 and to their appropriate reference genome (macaque, rheMac2; chimpanzee, panTro2) as indicated.

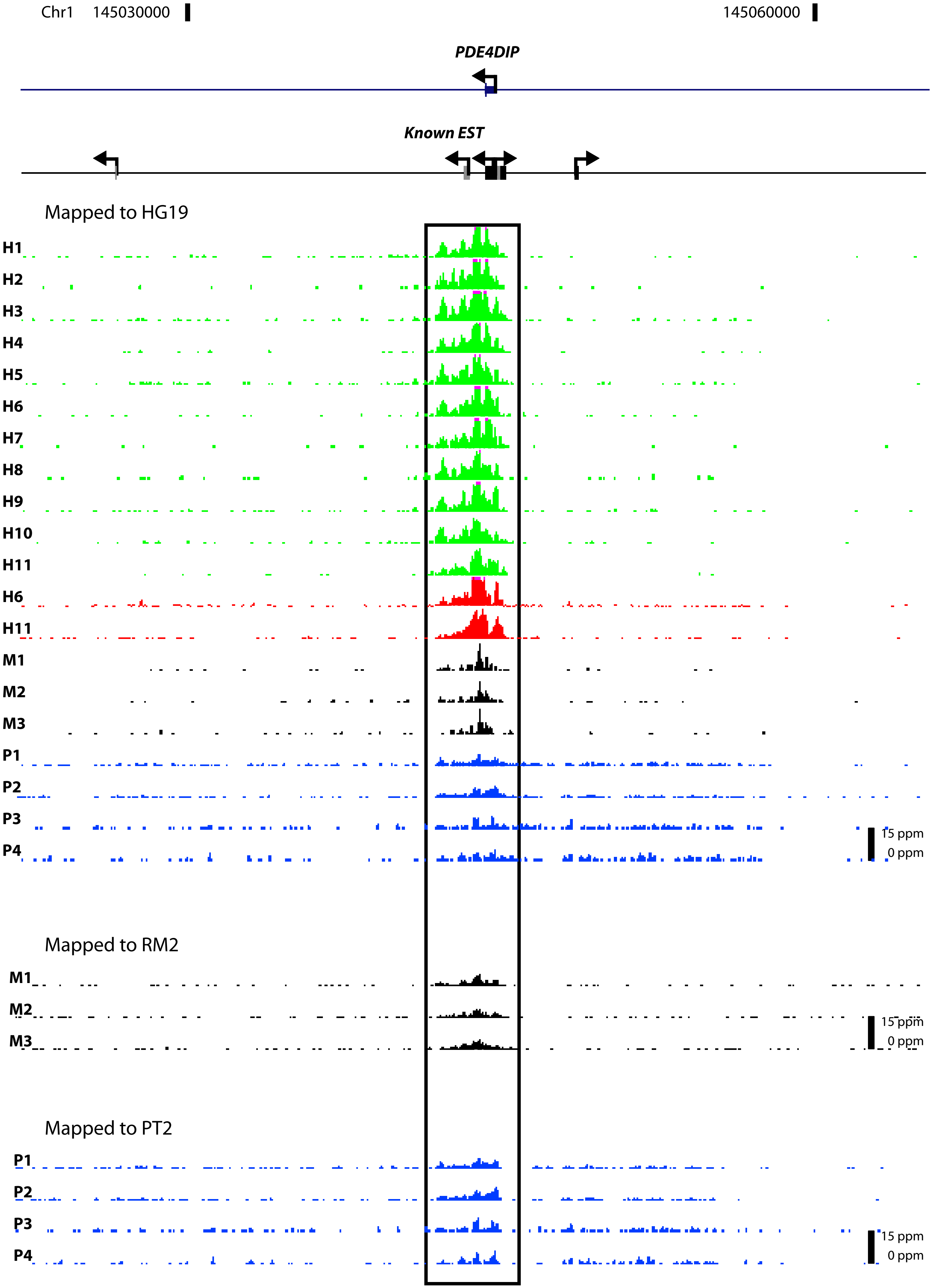

Chr1 145030000
145060000
PDE4DIP
Known EST
Mapped to HG19
H1
H2
H3
H4
H5
H6
H7
H8
H9
H10
H11
H6
H11
M1
M2
M3
P1
P2
P3
P4
15 ppm
0 ppm
Mapped to RM2
M1
M2
M3
15 ppm
0 ppm
Mapped to PT2
P1
P2
P3
P4
15 ppm
0 ppm

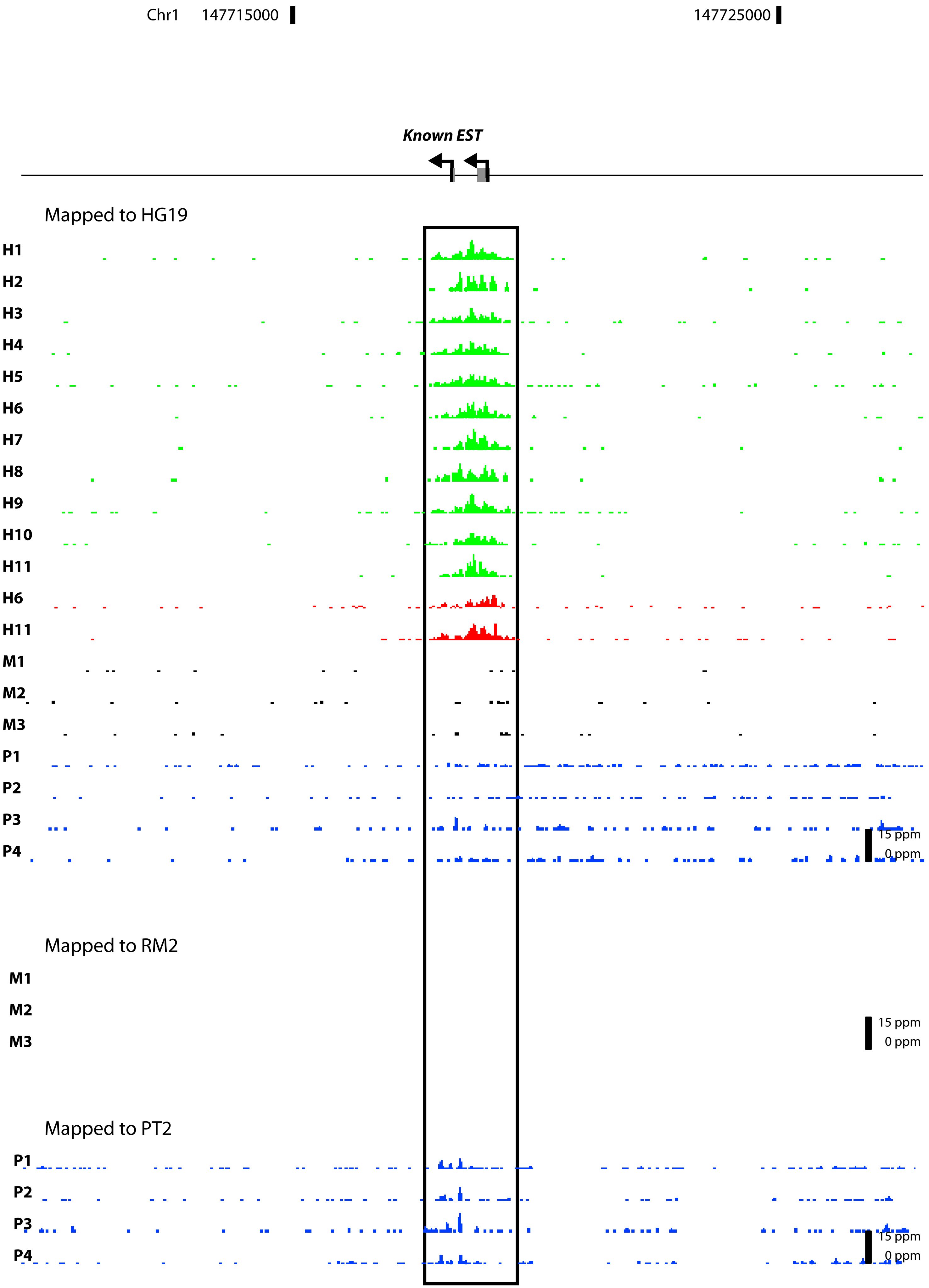

Chr1 147715000
147725000
Known EST
Mapped to HG19
H1
H2
H3
H4
H5
H6
H7
H8
H9
H10
H11
H6
H11
M1
M2
M3
P1
P2
P3
P4
15 ppm
0 ppm
Mapped to RM2
M1
M2
M3
15 ppm
0 ppm
Mapped to PT2
P1
P2
P3
P4
15 ppm
0 ppm

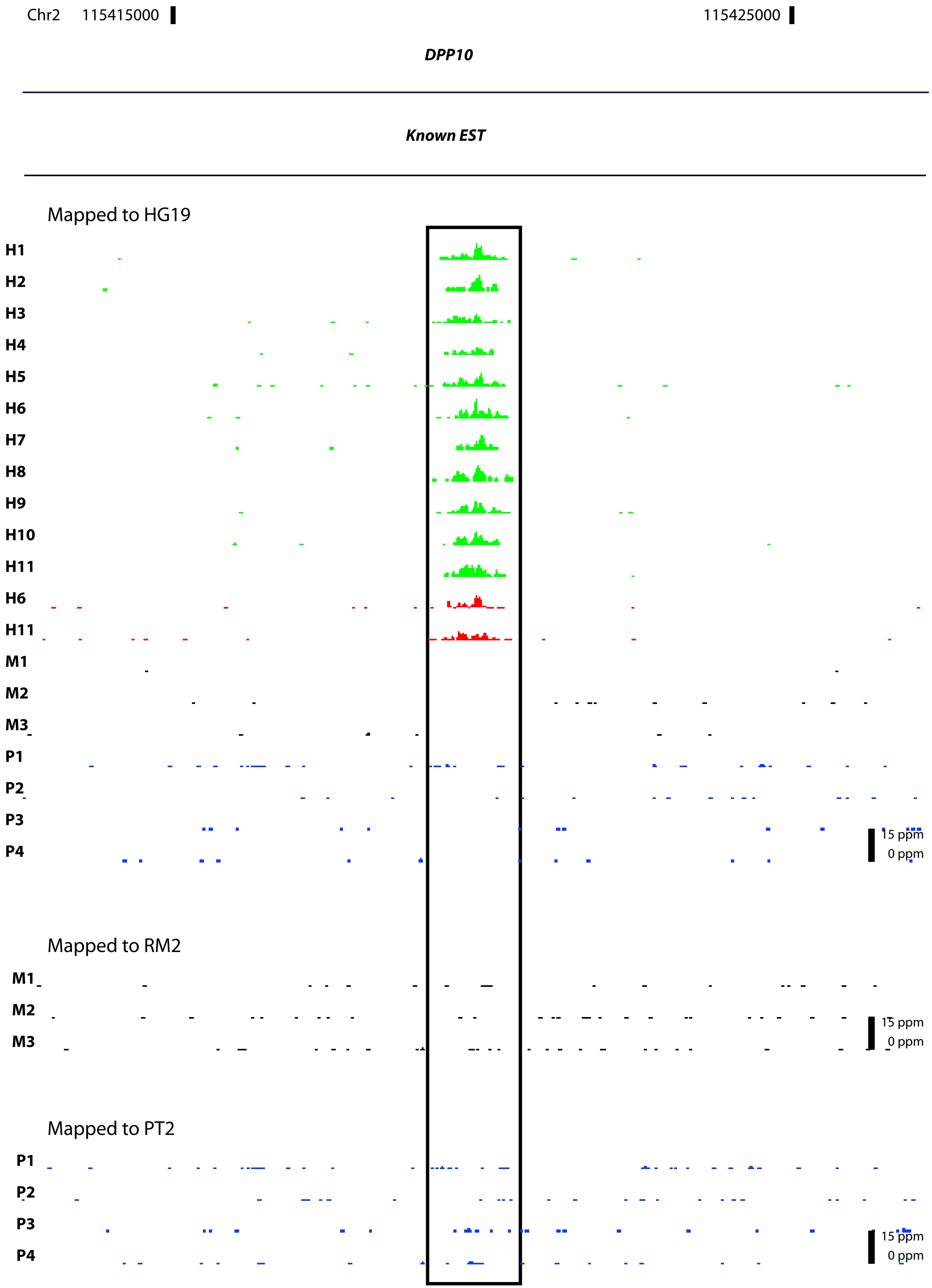

Chr2 115415000
115425000
DPP10
Known EST
Mapped to HG19
H1
H2
H3
H4
H5
H6
H7
H8
H9
H10
H11
H6
H11
M1
M2
M3
P1
P2
P3
P4
15 ppm
0 ppm
Mapped to RM2
M1
M2
M3
15 ppm
0 ppm
Mapped to PT2
P1
P2
P3
P4
15 ppm
0 ppm

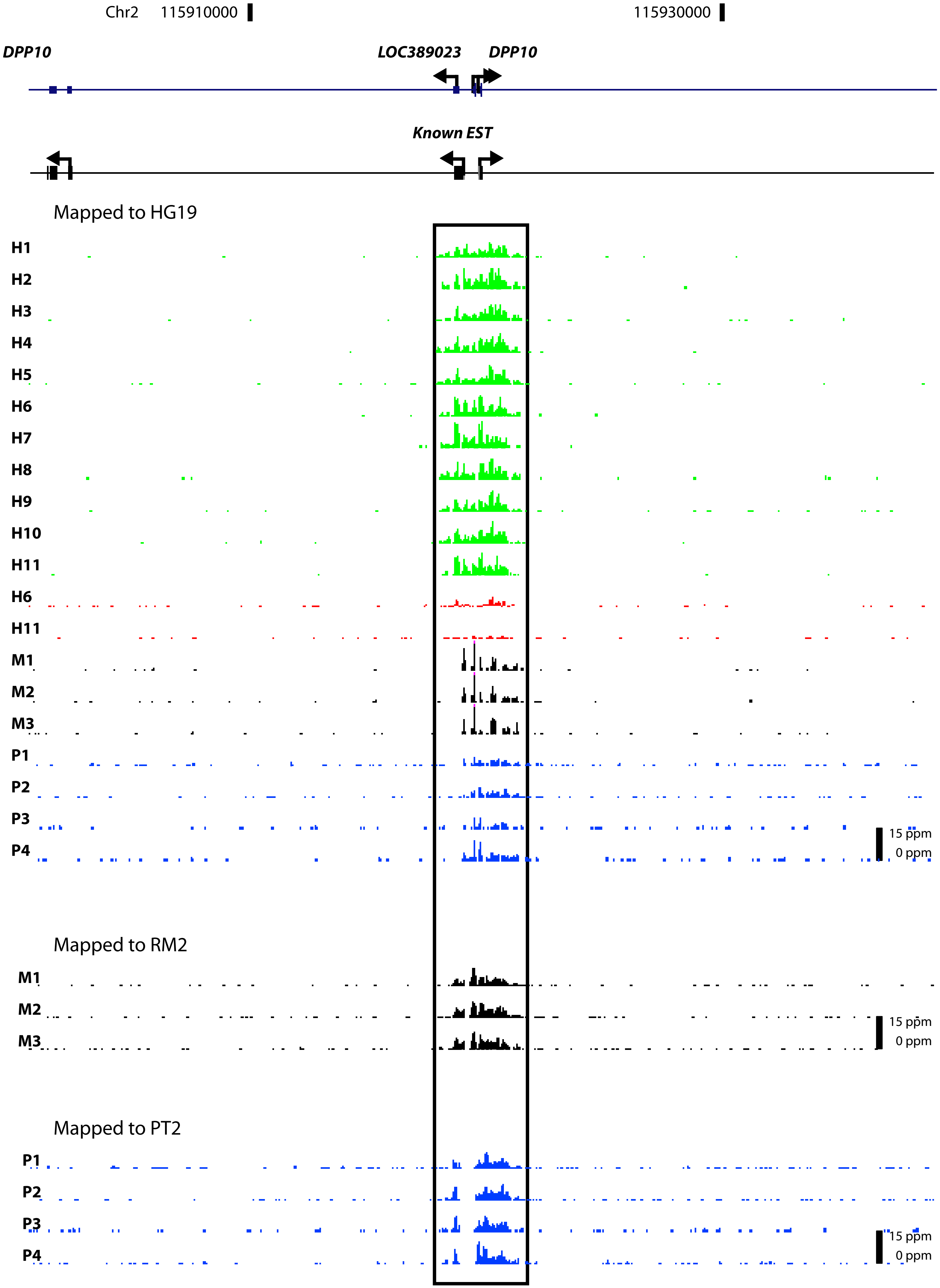

Chr2 115910000
115930000
DPP10
LOC389023
DPP10
Known EST
Mapped to HG19
H1
H2
H3
H4
H5
H6
H7
H8
H9
H10
H11
H6
H11
M1
M2
M3
P1
P2
P3
P4
15 ppm
0 ppm
Mapped to RM2
M1
M2
M3
15 ppm
0 ppm
Mapped to PT2
P1
P2
P3
P4
15 ppm
0 ppm

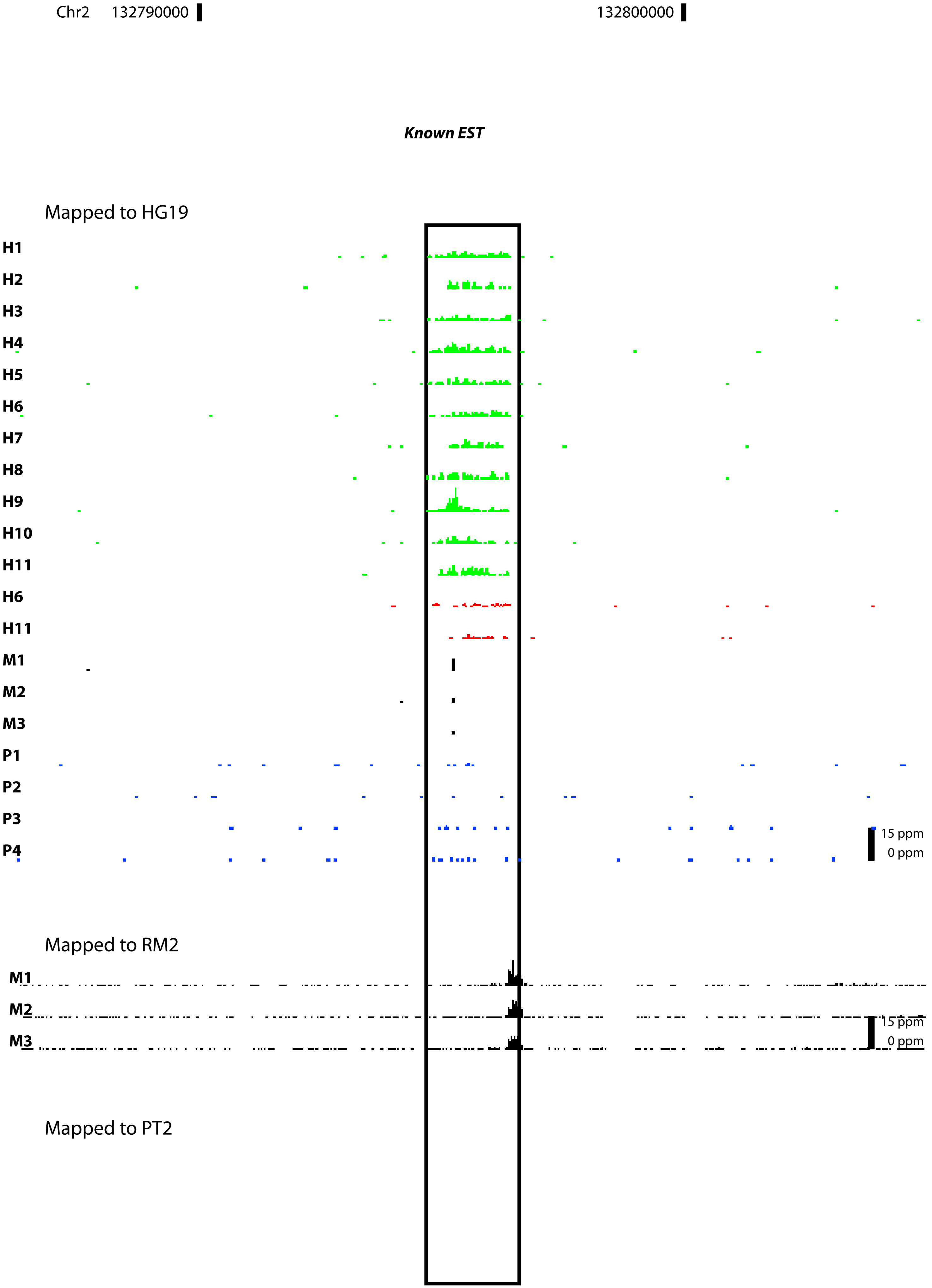
Chr2
132790000
132800000
Known EST
Mapped to HG19
H1
H2
H3
H4
H5
H6
H7
H8
H9
H10
H11
H6
H11
M1
M2
M3
P1
P2
P3
P4
15 ppm
0 ppm
Mapped to RM2
M1
M2
M3
15 ppm
0 ppm
Mapped to PT2

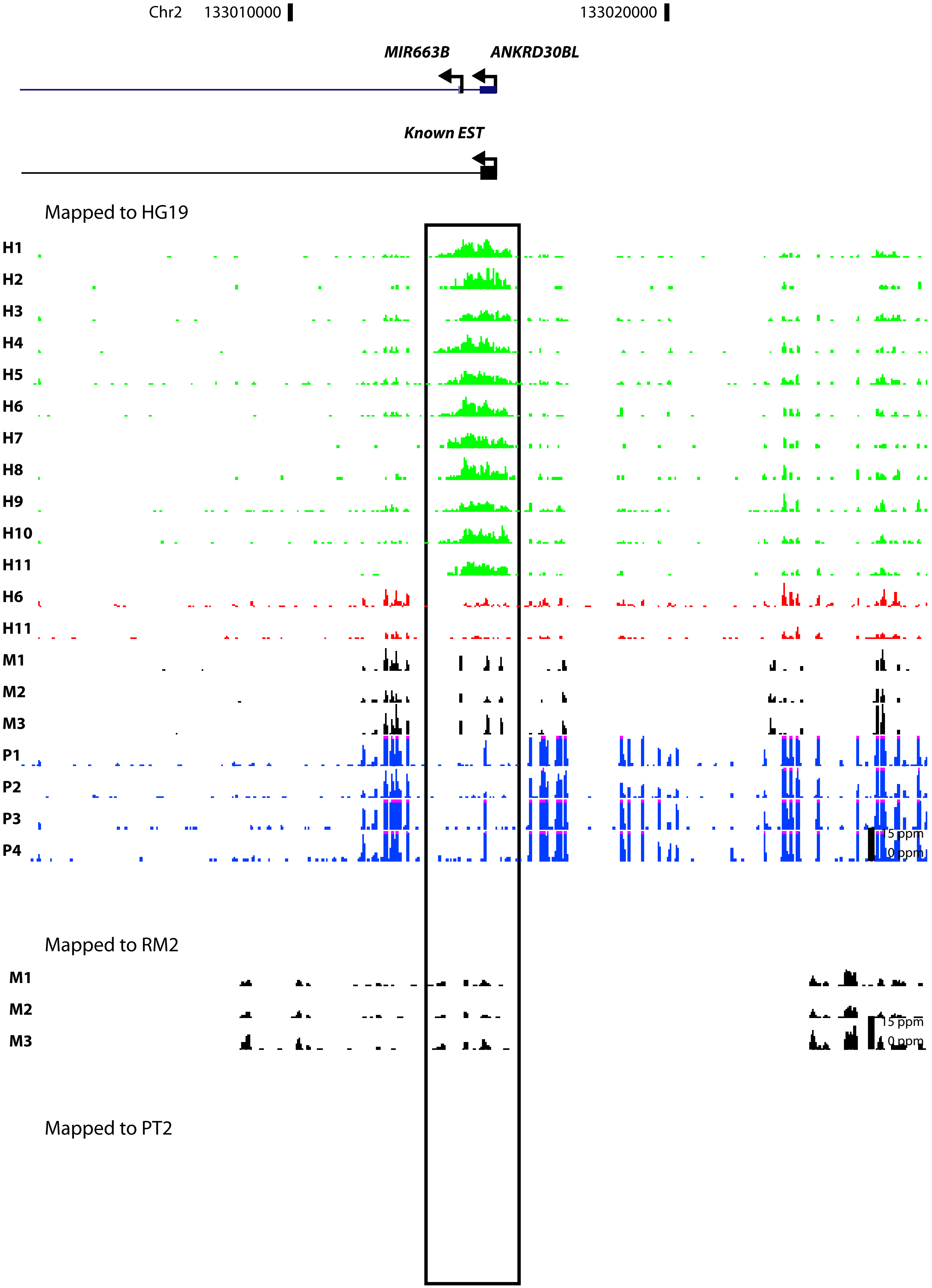

Chr2 133010000
133020000
MIR663B
ANKRD30BL
Known EST
Mapped to HG19
H1
H2
H3
H4
H5
H6
H7
H8
H9
H10
H11
H6
H11
M1
M2
M3
P1
P2
P3
P4
15 ppm
0 ppm
Mapped to RM2
M1
M2
M3
15 ppm
0 ppm
Mapped to PT2

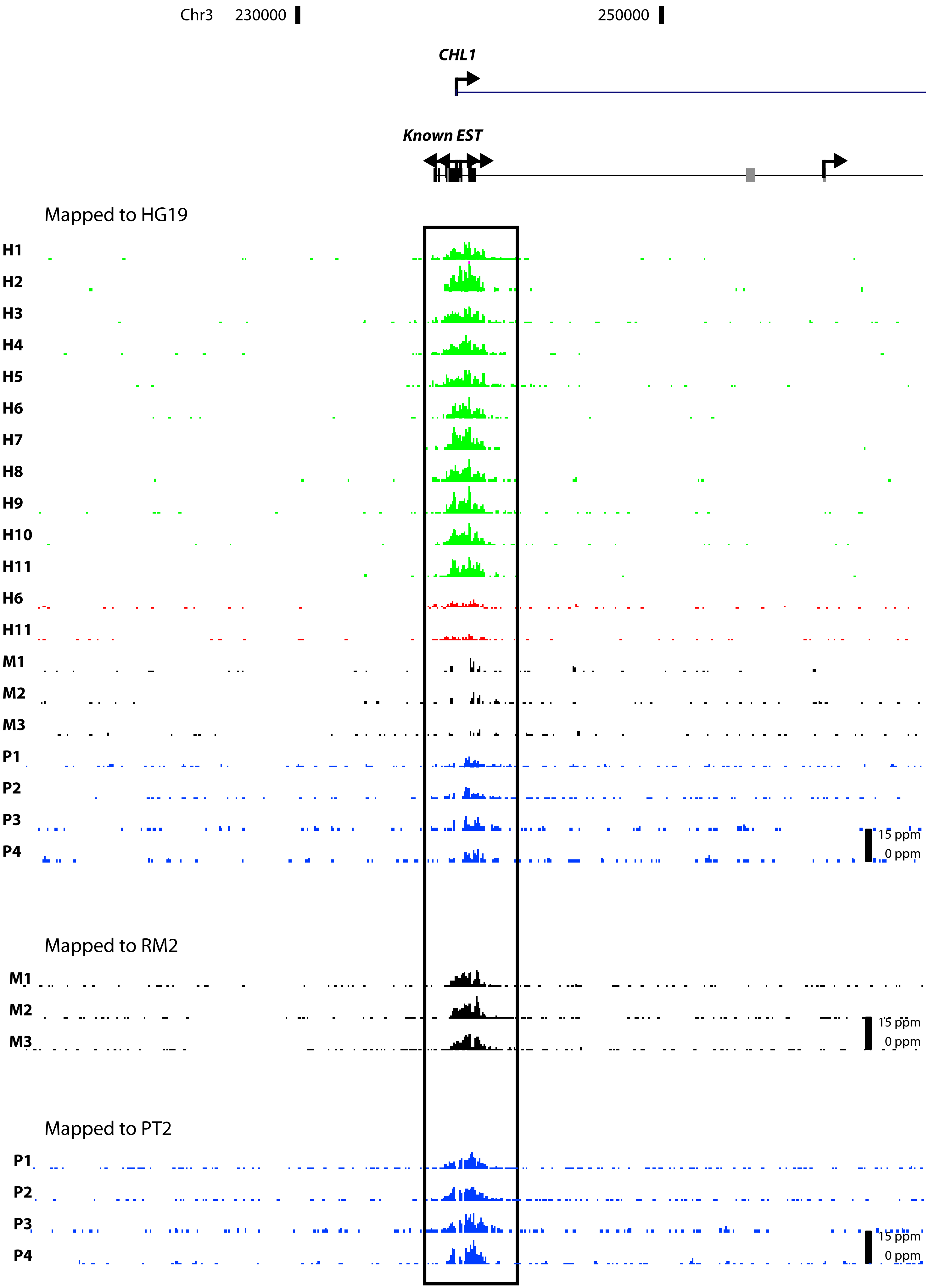

Chr3 230000
250000
CHL1
Known EST
Mapped to HG19
H1
H2
H3
H4
H5
H6
H7
H8
H9
H10
H11
H6
H11
M1
M2
M3
P1
P2
P3
P4
15 ppm
0 ppm
Mapped to RM2
M1
M2
M3
15 ppm
0 ppm
Mapped to PT2
P1
P2
P3
P4
15 ppm
0 ppm

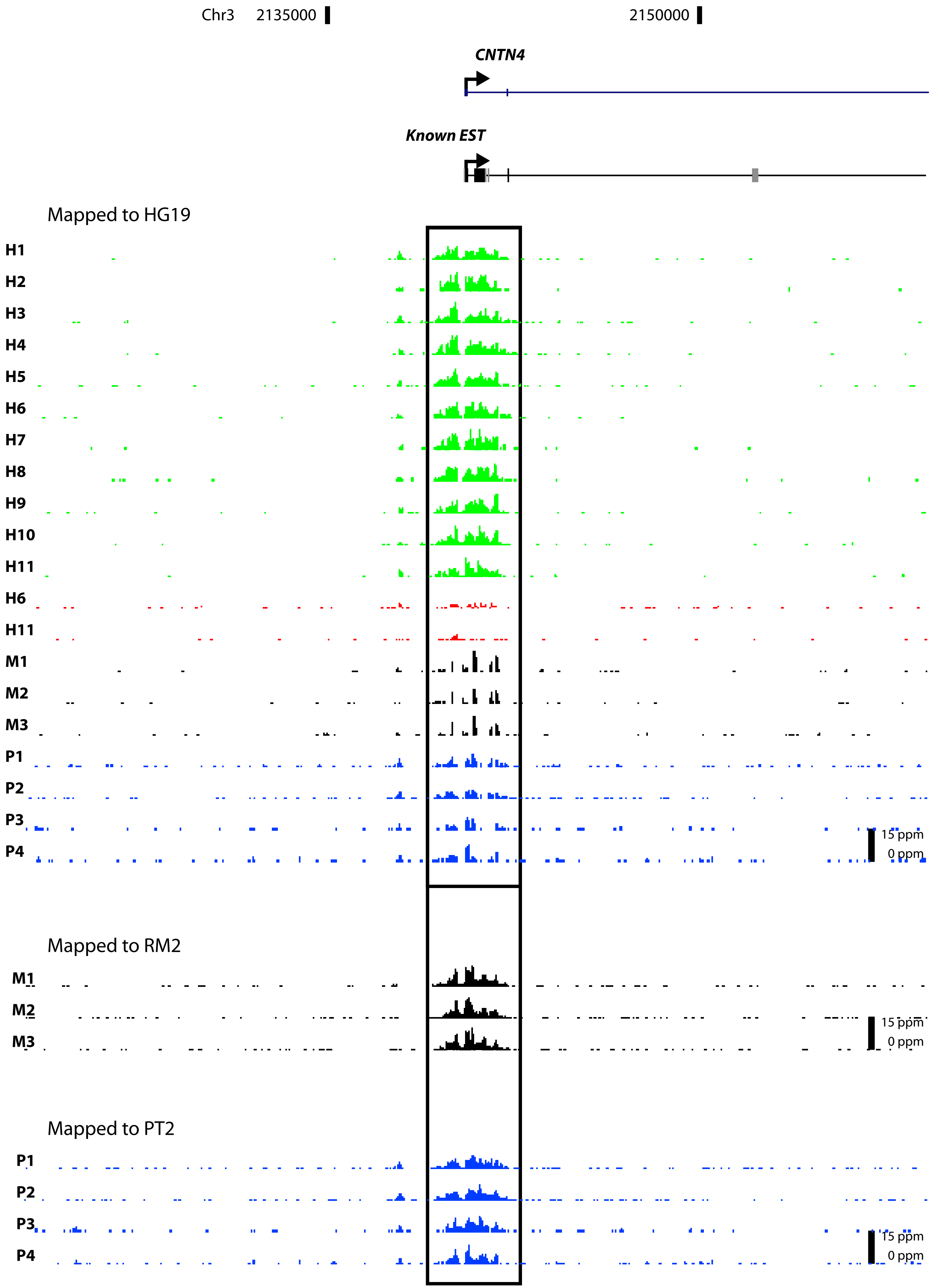

Chr3 2135000
2150000
CNTN4
Known EST
Mapped to HG19
H1
H2
H3
H4
H5
H6
H7
H8
H9
H10
H11
H6
H11
M1
M2
M3
P1
P2
P3
P4
15 ppm
0 ppm
Mapped to RM2
M1
M2
M3
15 ppm
0 ppm
Mapped to PT2
P1
P2
P3
P4
15 ppm
0 ppm

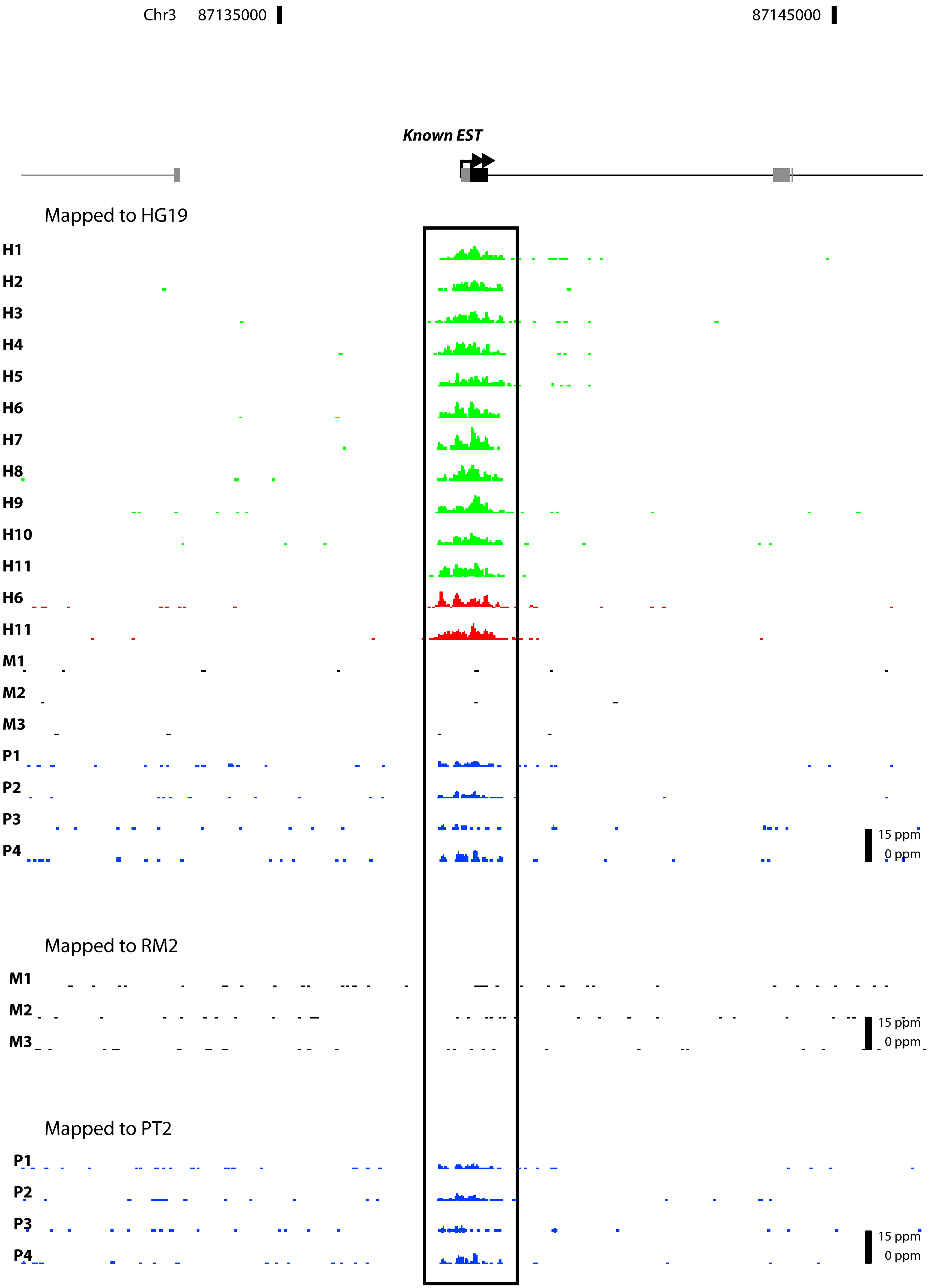

Chr3 87135000
87145000
Known EST
Mapped to HG19
H1
H2
H3
H4
H5
H6
H7
H8
H9
H10
H11
H6
H11
M1
M2
M3
P1
P2
P3
P4
15 ppm
0 ppm
Mapped to RM2
M1
M2
M3
15 ppm
0 ppm
Mapped to PT2
P1
P2
P3
P4
15 ppm
0 ppm

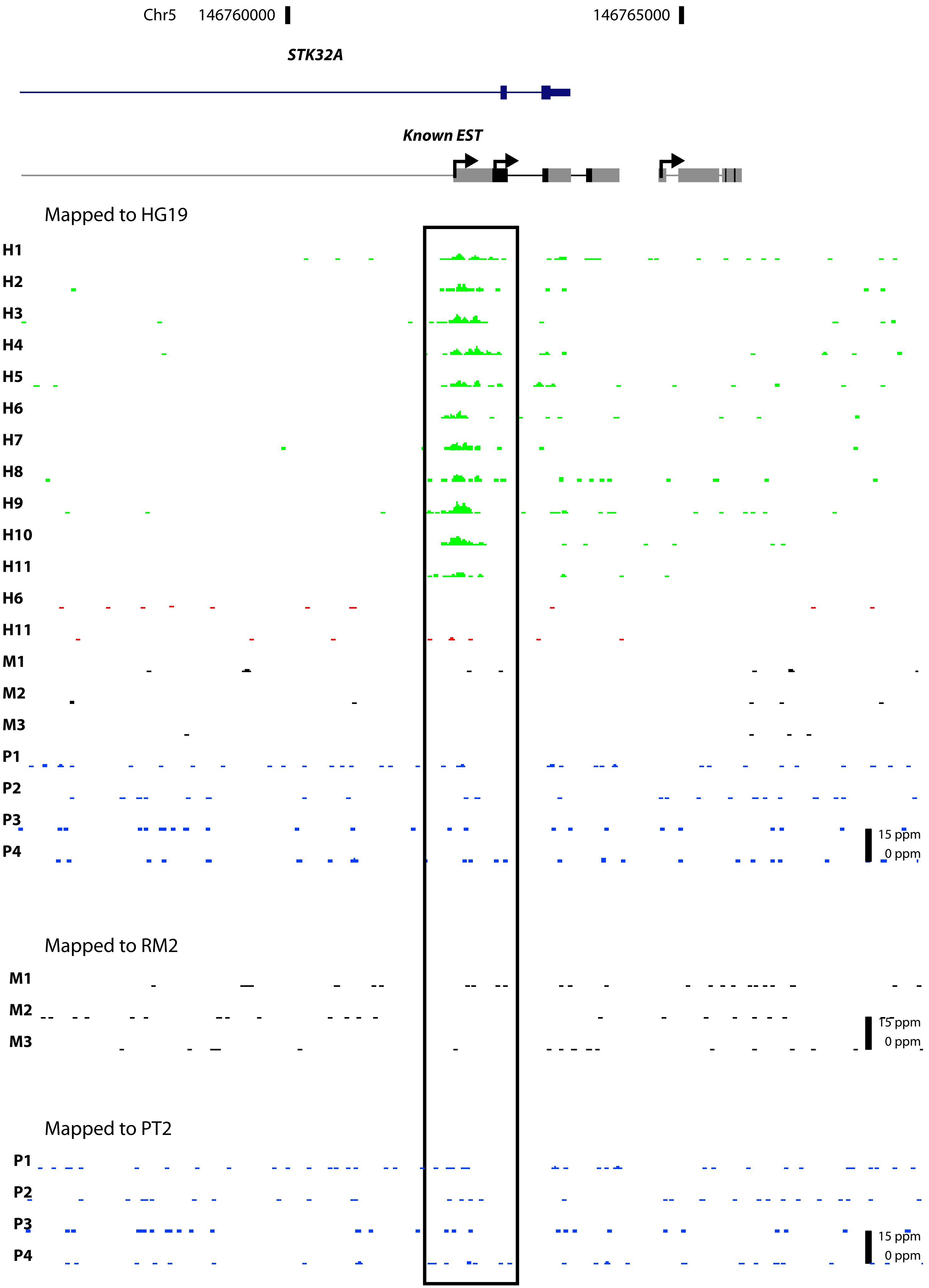

Chr5 146760000
146765000
STK32A
Known EST
Mapped to HG19
H1
H2
H3
H4
H5
H6
H7
H8
H9
H10
H11
H6
H11
M1
M2
M3
P1
P2
P3
P4
15 ppm
0 ppm
Mapped to RM2
M1
M2
M3
15 ppm
0 ppm
Mapped to PT2
P1
P2
P3
P4
15 ppm
0 ppm

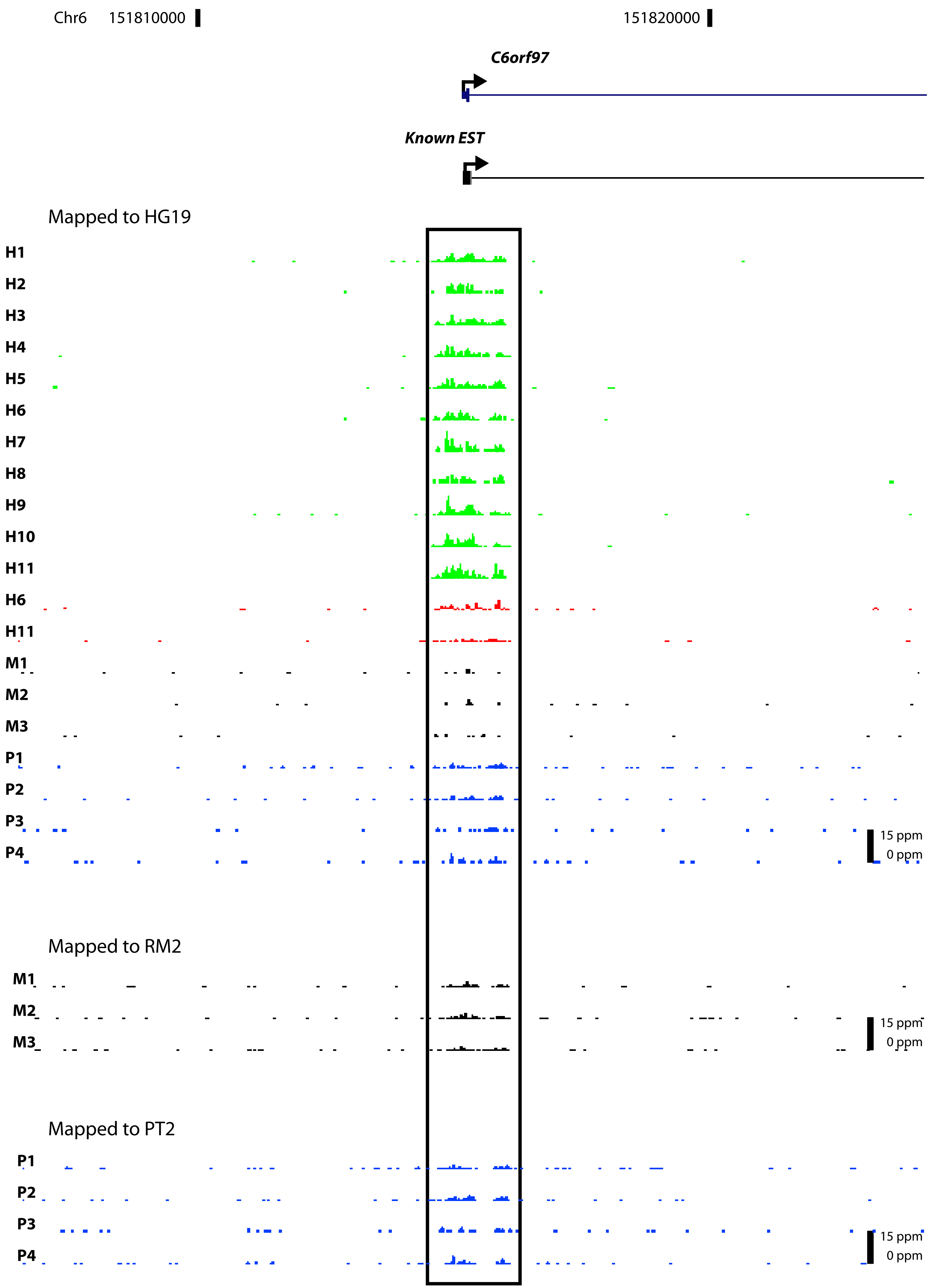

Chr6 151810000
151820000
C6orf97
Known EST
Mapped to HG19
H1
H2
H3
H4
H5
H6
H7
H8
H9
H10
H11
H6
H11
M1
M2
M3
P1
P2
P3
P4
15 ppm
0 ppm
Mapped to RM2
M1
M2
M3
15 ppm
0 ppm
Mapped to PT2
P1
P2
P3
P4
15 ppm
0 ppm

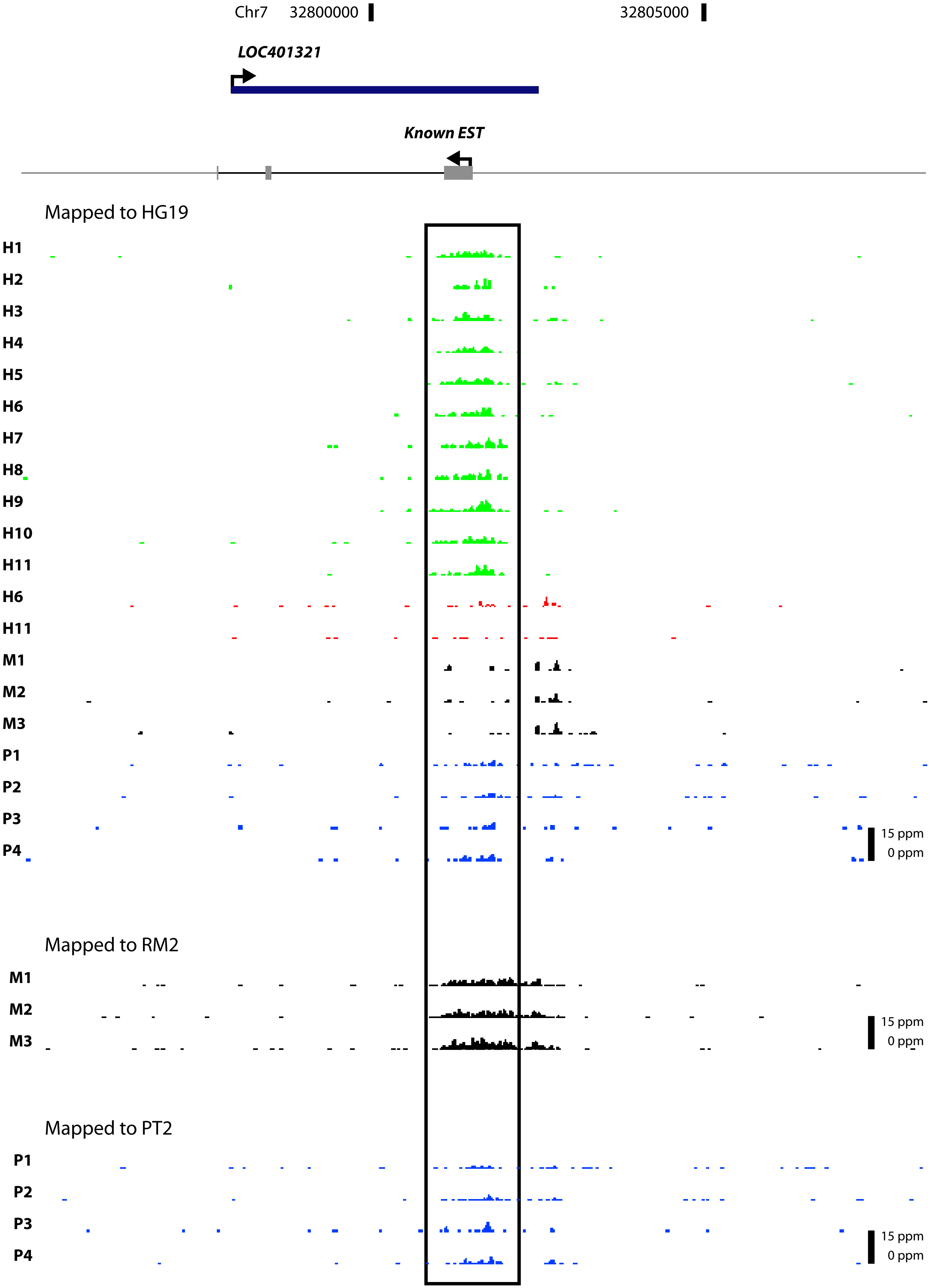

Chr7
32800000
32805000
LOC401321
Known EST
Mapped to HG19
H1
H2
H3
H4
H5
H6
H7
H8
H9
H10
H11
H6
H11
M1
M2
M3
P1
P2
P3
P4
15 ppm
0 ppm
Mapped to RM2
M1
M2
M3
15 ppm
0 ppm
Mapped to PT2
P1
P2
P3
P4
15 ppm
0 ppm

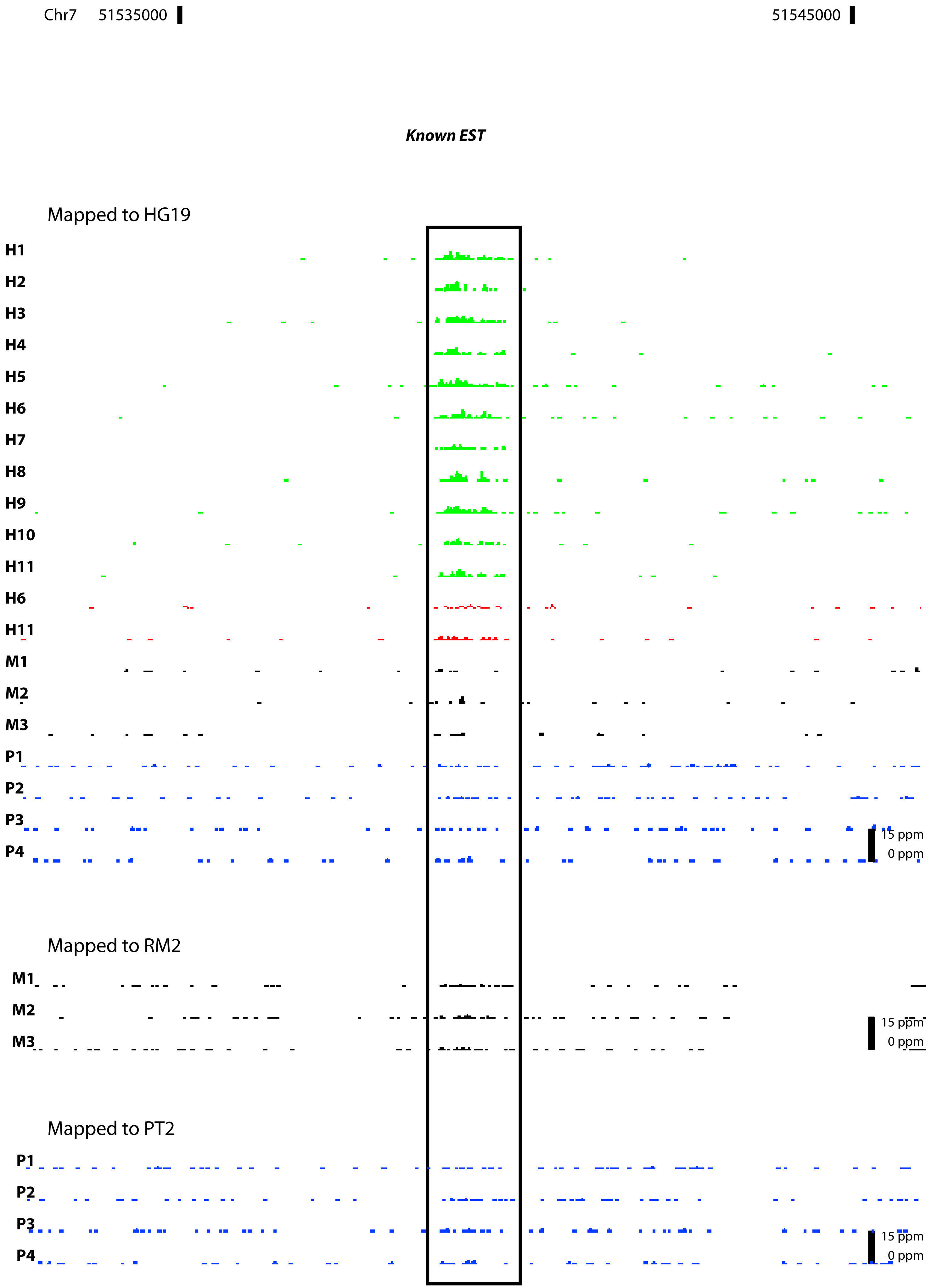

Chr7
51535000
51545000
Known EST
Mapped to HG19
H1
H2
H3
H4
H5
H6
H7
H8
H9
H10
H11
H6
H11
M1
M2
M3
P1
P2
P3
P4
15 ppm
0 ppm
Mapped to RM2
M1
M2
M3
15 ppm
0 ppm
Mapped to PT2
P1
P2
P3
P4
15 ppm
0 ppm

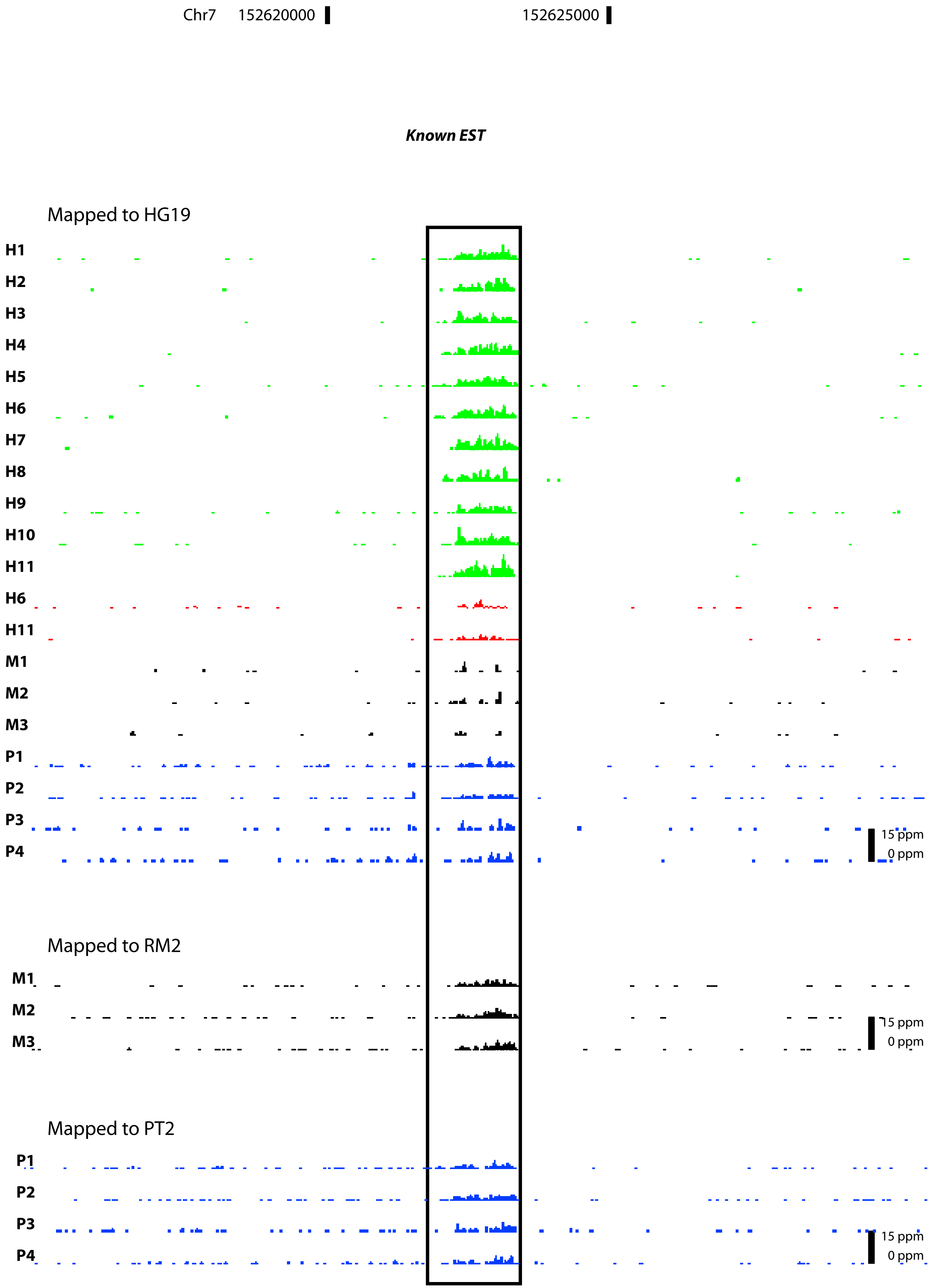
Chr7
152620000
152625000
Known EST
Mapped to HG19
H1
H2
H3
H4
H5
H6
H7
H8
H9
H10
H11
H6
H11
M1
M2
M3
P1
P2
P3
P4
15 ppm
0 ppm
Mapped to RM2
M1
M2
M3
15 ppm
0 ppm
Mapped to PT2
P1
P2
P3
P4
15 ppm
0 ppm

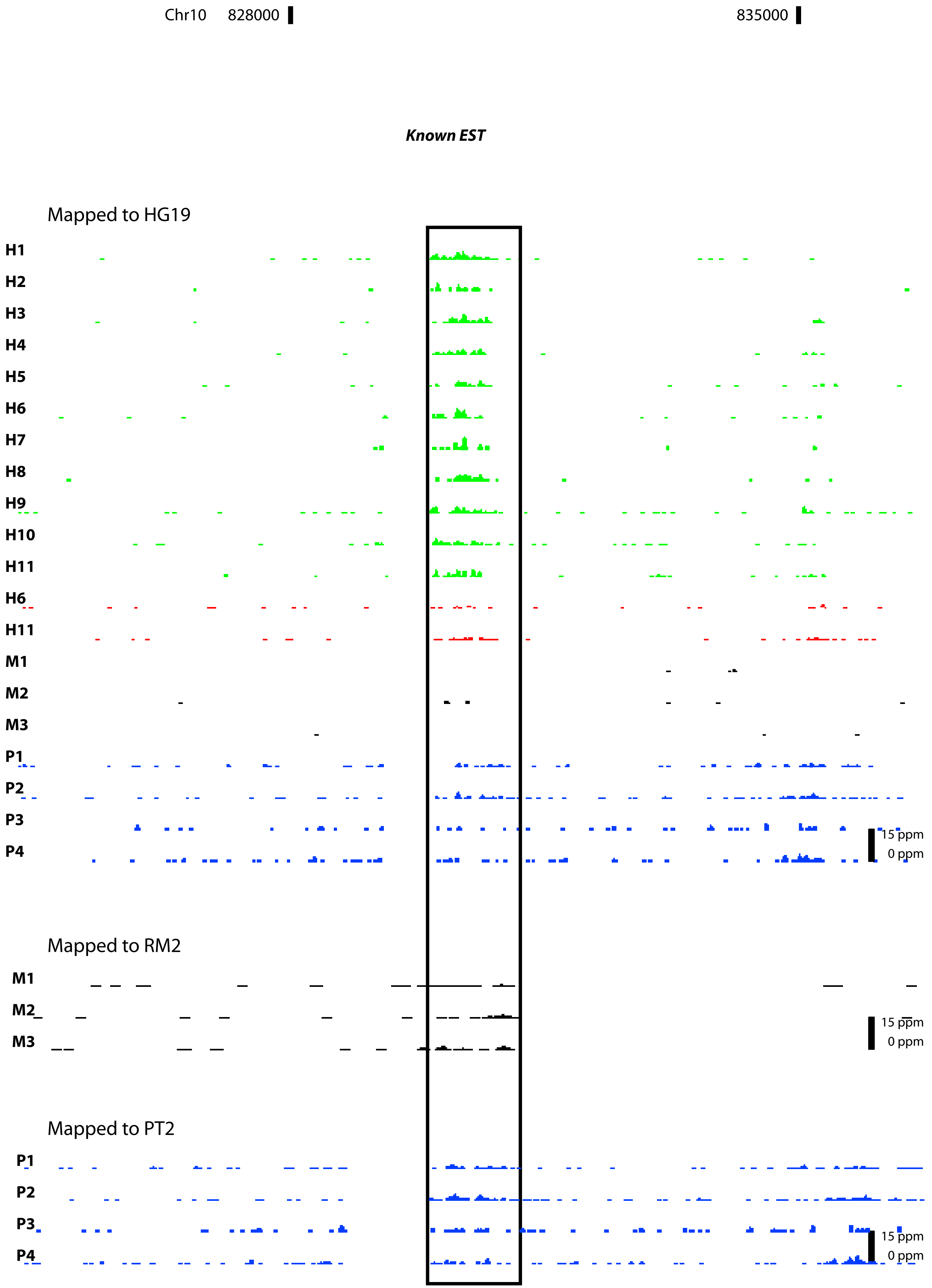

Chr10 828000
835000
Known EST
Mapped to HG19
H1
H2
H3
H4
H5
H6
H7
H8
H9
H10
H11
H6
H11
M1
M2
M3
P1
P2
P3
P4
15 ppm
0 ppm
Mapped to RM2
M1
M2
M3
15 ppm
0 ppm
Mapped to PT2
P1
P2
P3
P4
15 ppm
0 ppm

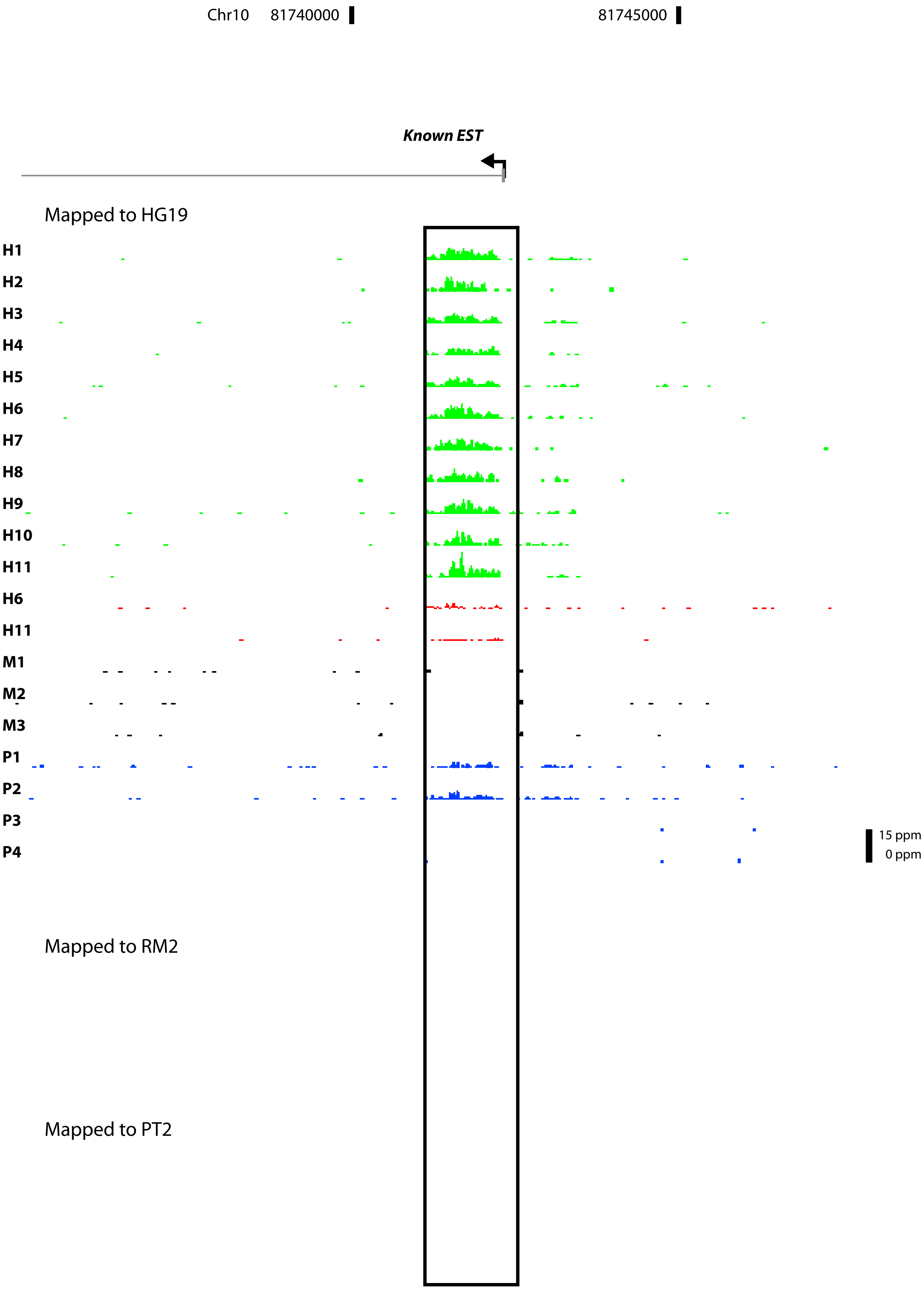

Chr10
81740000
81745000
Known EST
Mapped to HG19
H1
H2
H3
H4
H5
H6
H7
H8
H9
H10
H11
H6
H11
M1
M2
M3
P1
P2
P3
P4
15 ppm
0 ppm
Mapped to RM2
Mapped to PT2

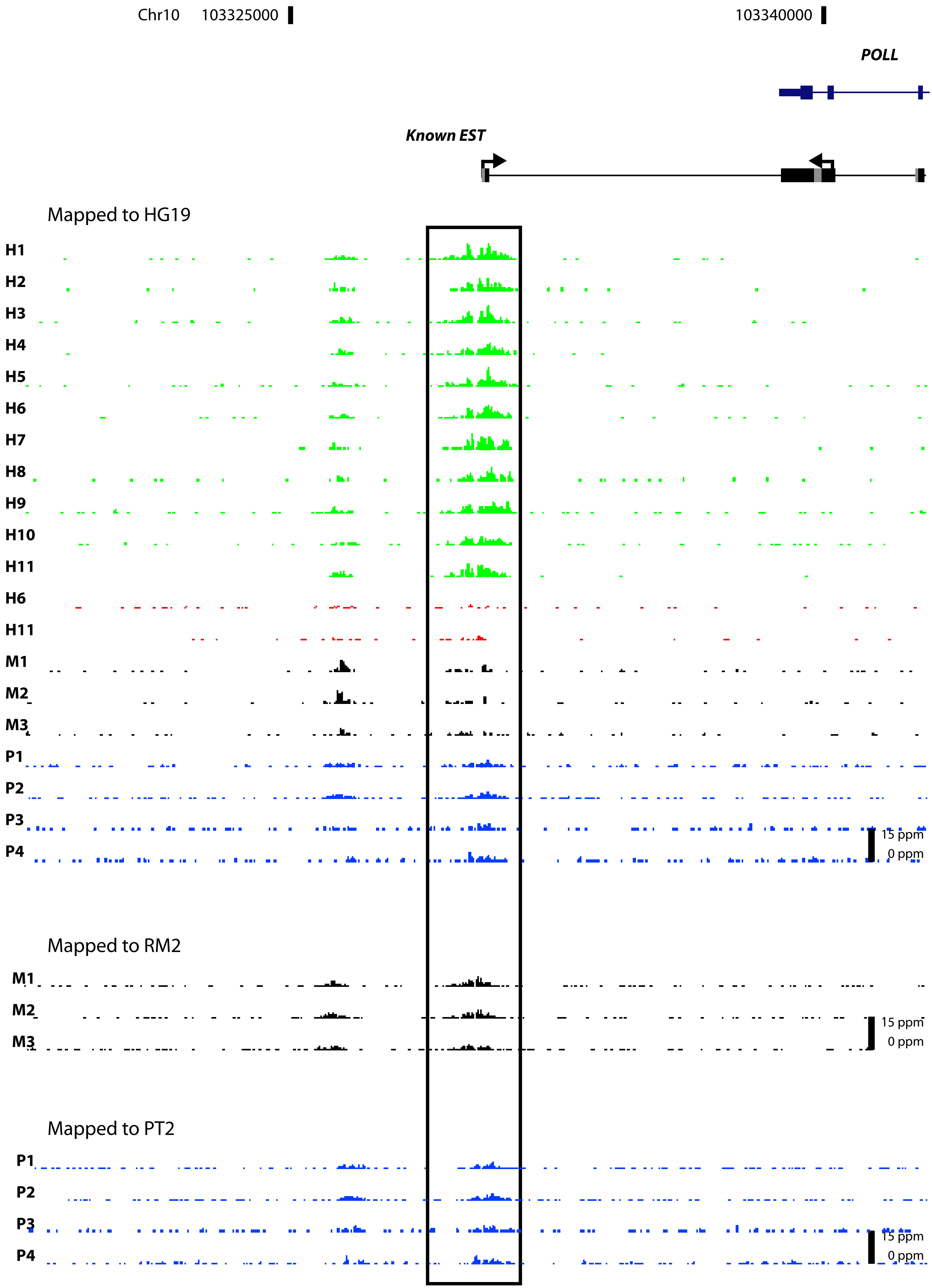
Chr10
103325000
103340000
POLL
Known EST
Mapped to HG19
H1
H2
H3
H4
H5
H6
H7
H8
H9
H10
H11
H6
H11
M1
M2
M3
P1
P2
P3
P4
15 ppm
0 ppm
Mapped to RM2
M1
M2
M3
15 ppm
0 ppm
Mapped to PT2
P1
P2
P3
P4
15 ppm
0 ppm

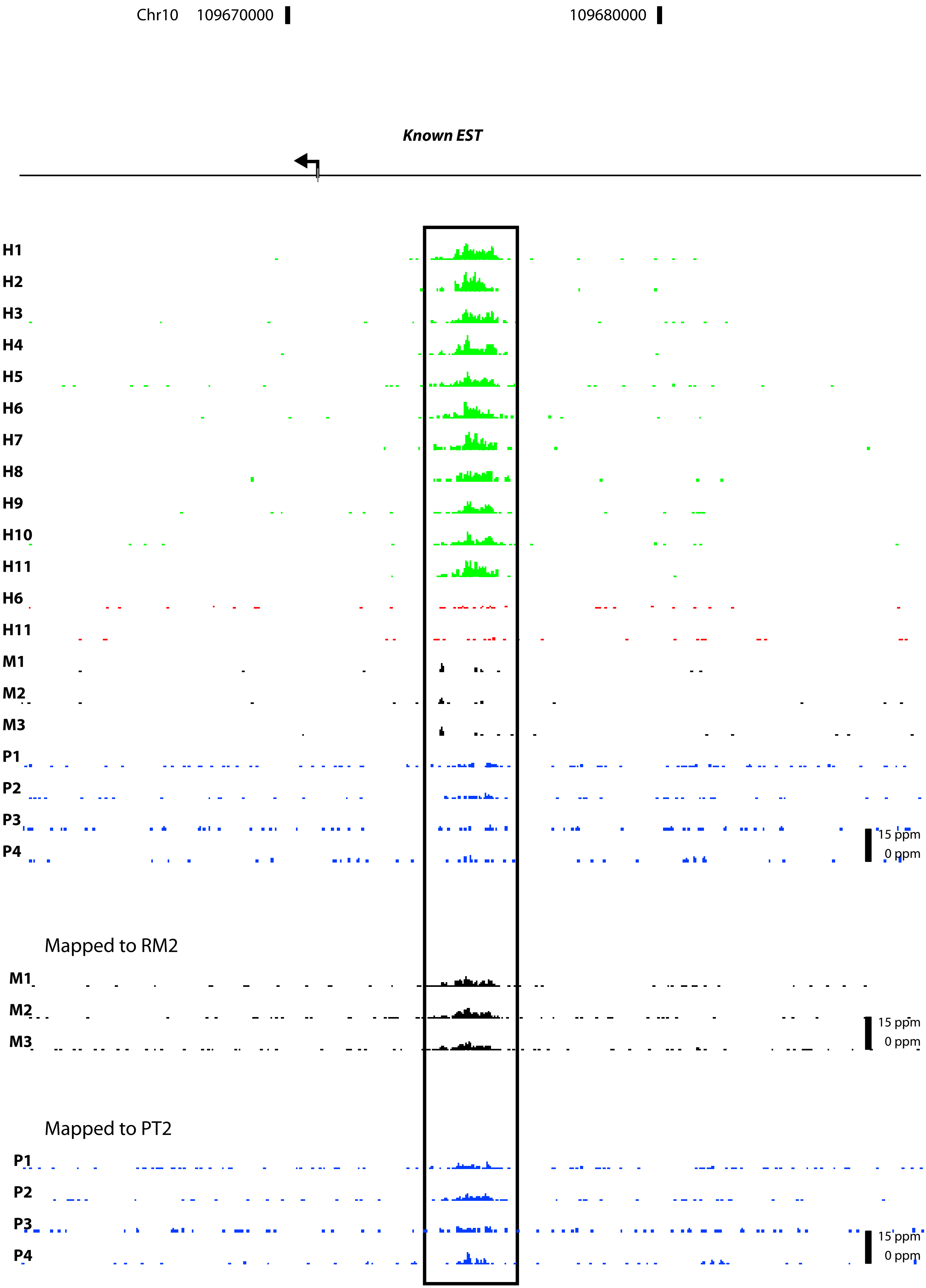
Chr10 109670000
109680000
Known EST
H1
H2
H3
H4
H5
H6
H7
H8
H9
H10
H11
H6
H11
M1
M2
M3
P1
P2
P3
P4
15 ppm
0 ppm
Mapped to RM2
M1
M2
M3
15 ppm
0 ppm
Mapped to PT2
P1
P2
P3
P4
15 ppm
0 ppm

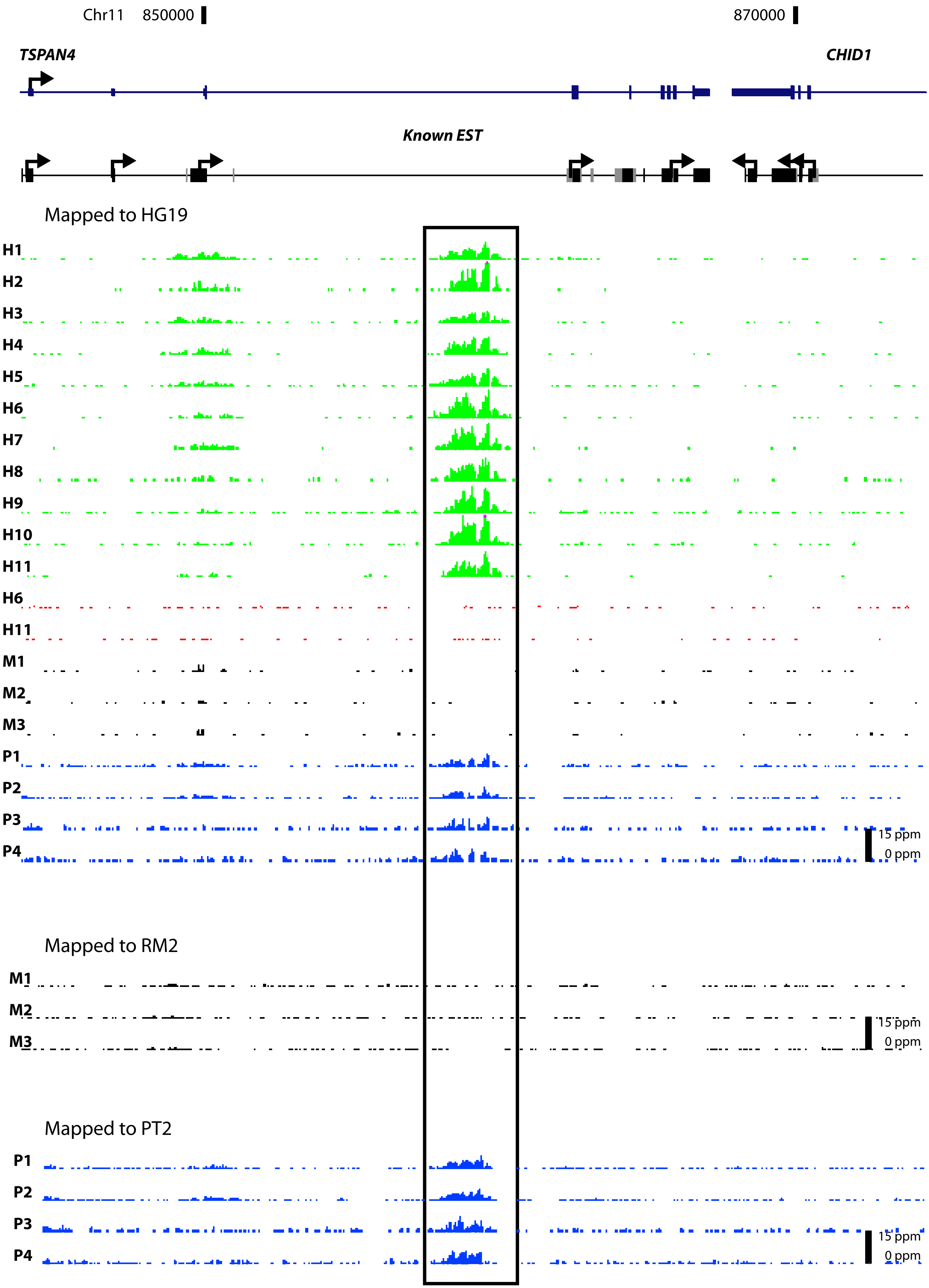

Chr11
850000
870000
TSPAN4
CHID1
Known EST
Mapped to HG19
H1
H2
H3
H4
H5
H6
H7
H8
H9
H10
H11
H6
H11
M1
M2
M3
P1
P2
P3
P4
15 ppm
0 ppm
Mapped to RM2
M1
M2
M3
15 ppm
0 ppm
Mapped to PT2
P1
P2
P3
P4
15 ppm
0 ppm

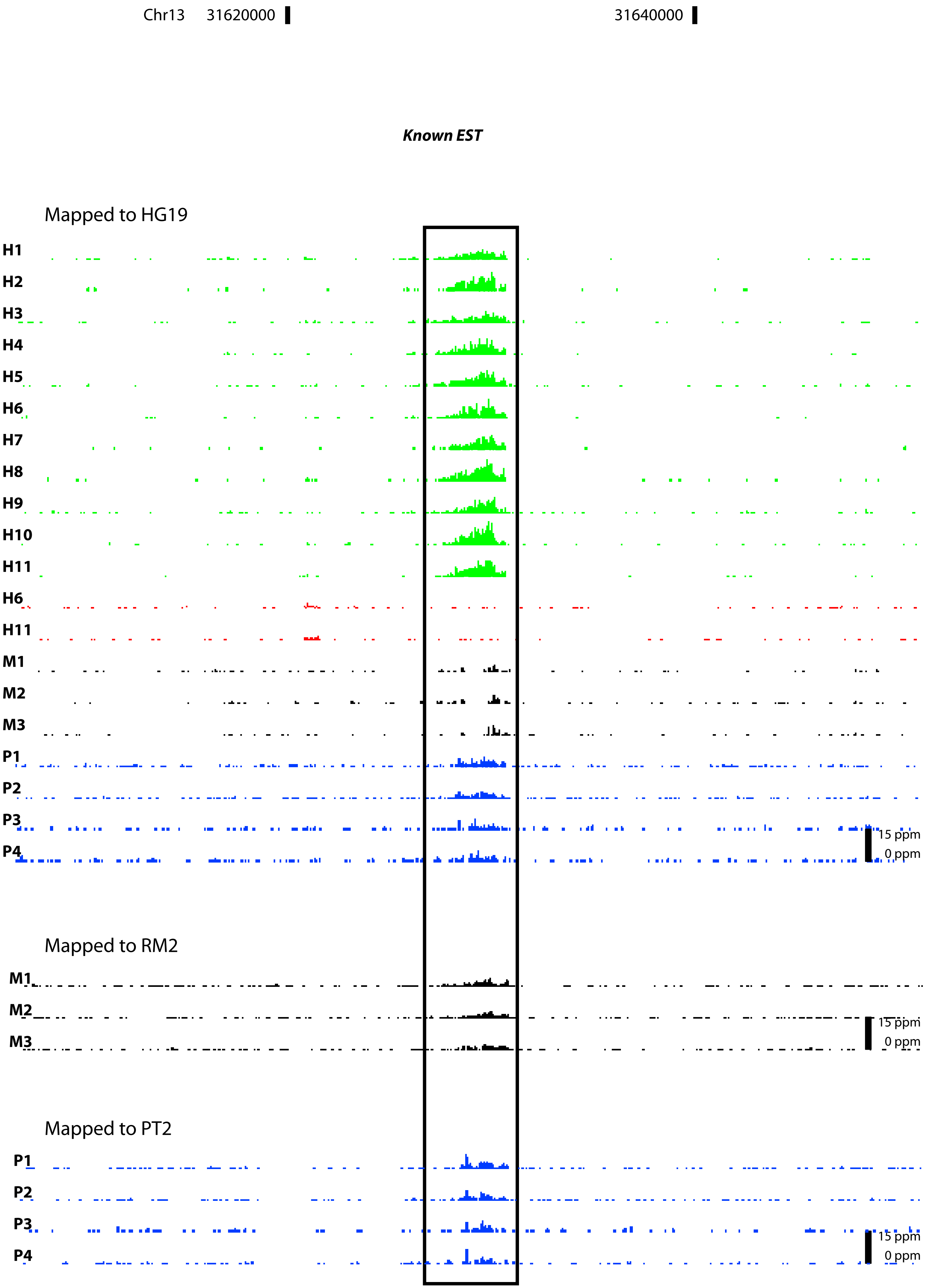
Chr13 31620000
31640000
Known EST
Mapped to HG19
H1
H2
H3
H4
H5
H6
H7
H8
H9
H10
H11
H6
H11
M1
M2
M3
P1
P2
P3
P4
15 ppm
0 ppm
Mapped to RM2
M1
M2
M3
15 ppm
0 ppm
Mapped to PT2
P1
P2
P3
P4
15 ppm
0 ppm

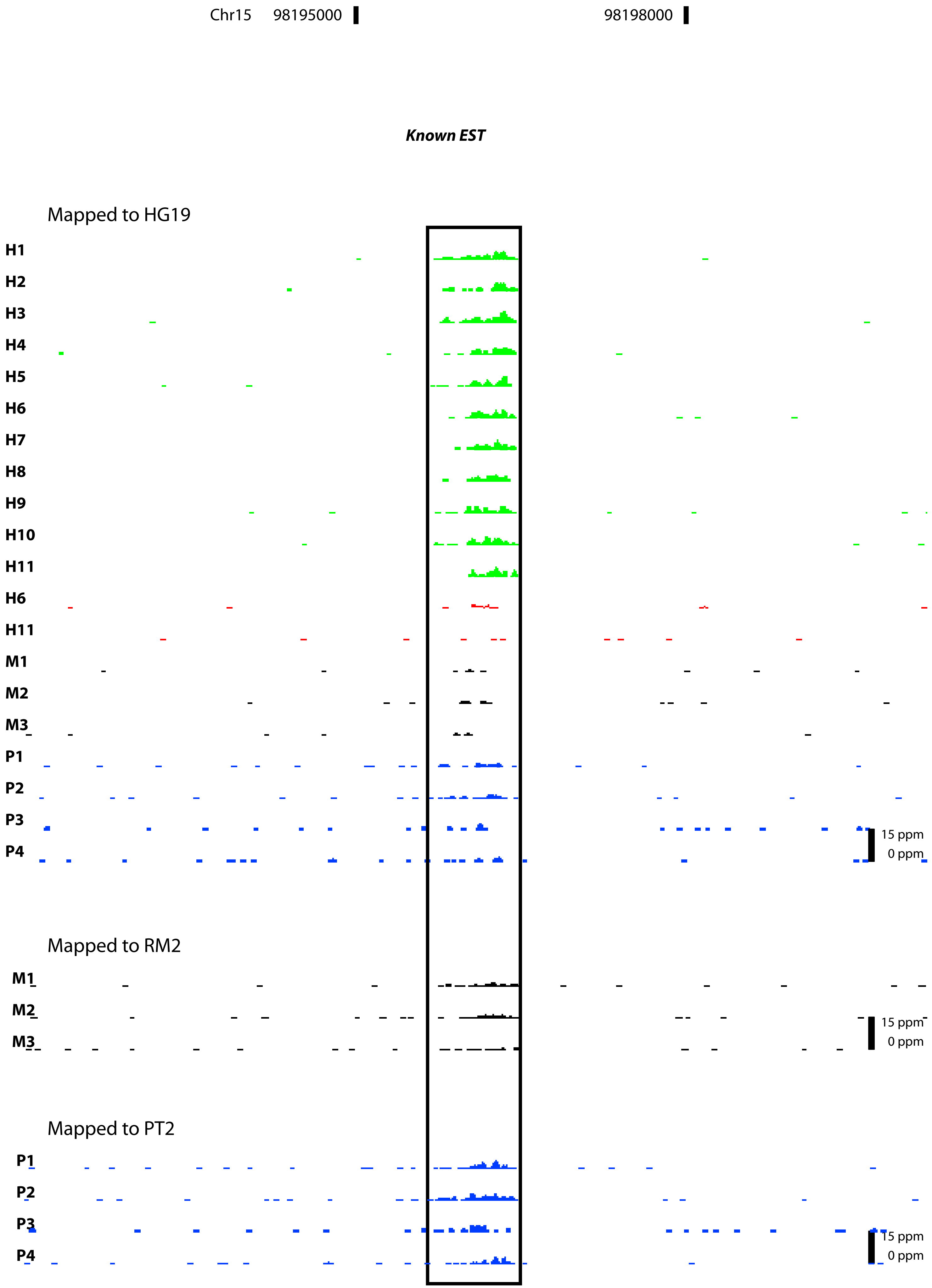

Chr15 98195000
98198000
Known EST
Mapped to HG19
H1
H2
H3
H4
H5
H6
H7
H8
H9
H10
H11
H6
H11
M1
M2
M3
P1
P2
P3
P4
15 ppm
0 ppm
Mapped to RM2
M1
M2
M3
15 ppm
0 ppm
Mapped to PT2
P1
P2
P3
P4
15 ppm
0 ppm

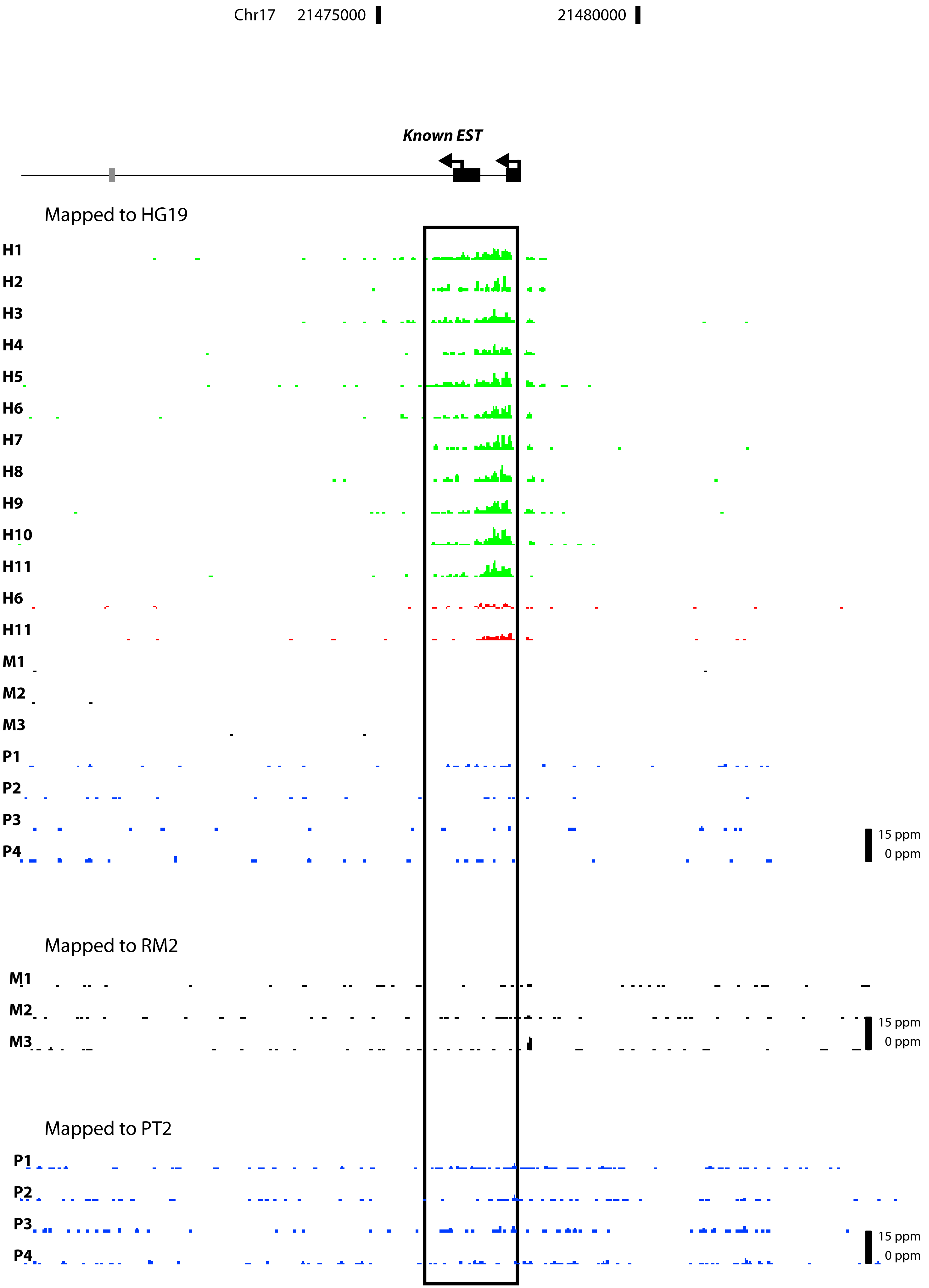

Chr17 21475000
21480000
Known EST
Mapped to HG19
H1
H2
H3
H4
H5
H6
H7
H8
H9
H10
H11
H6
H11
M1
M2
M3
P1
P2
P3
P4
15 ppm
0 ppm
Mapped to RM2
M1
M2
M3
15 ppm
0 ppm
Mapped to PT2
P1
P2
P3
P4
15 ppm
0 ppm

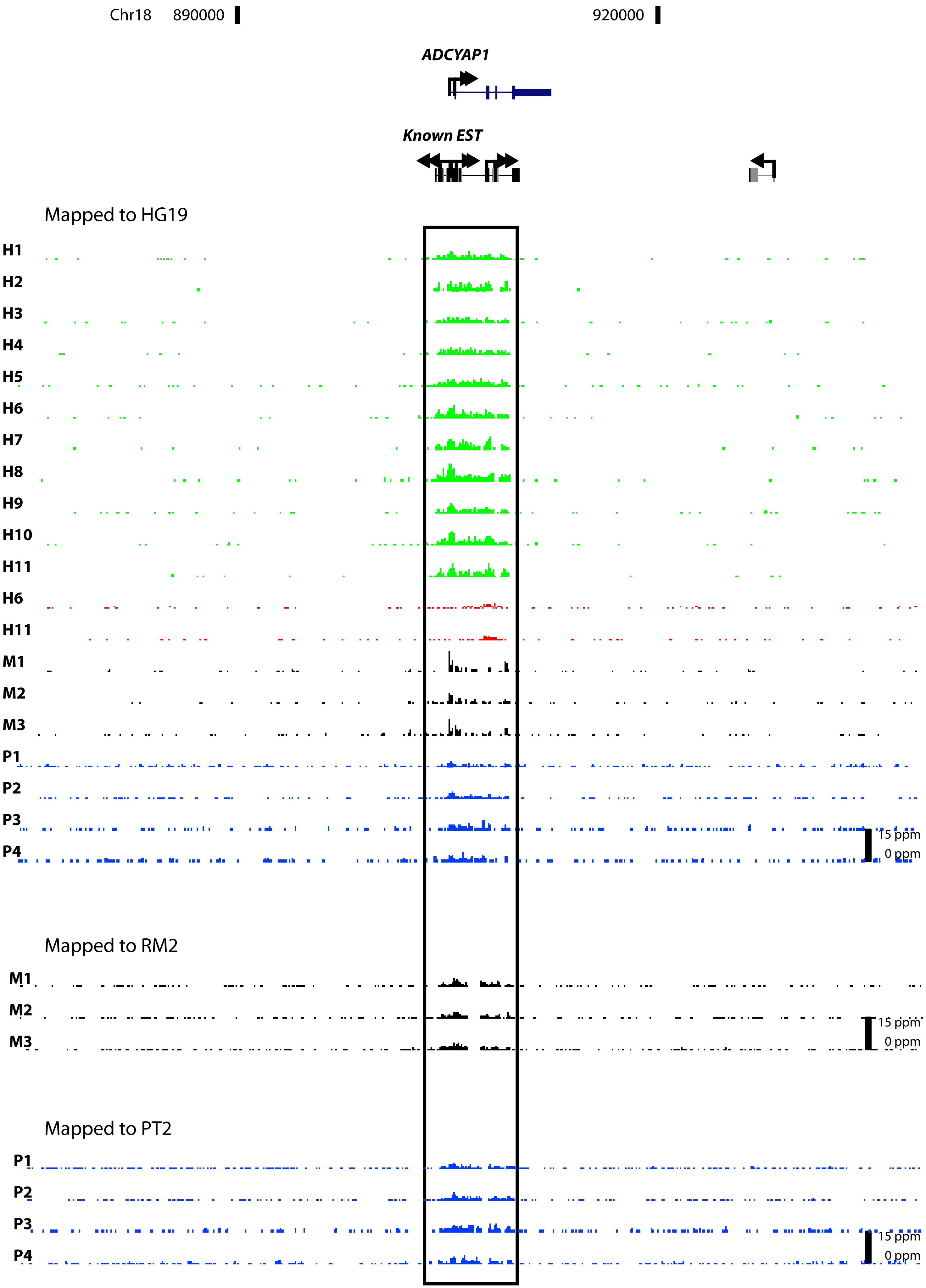
Chr18 890000
920000
ADCYAP1
Known EST
Mapped to HG19
H1
H2
H3
H4
H5
H6
H7
H8
H9
H10
H11
H6
H11
M1
M2
M3
P1
P2
P3
P4
15 ppm
0 ppm
Mapped to RM2
M1
M2
M3
15 ppm
0 ppm
Mapped to PT2
P1
P2
P3
P4
15 ppm
0 ppm

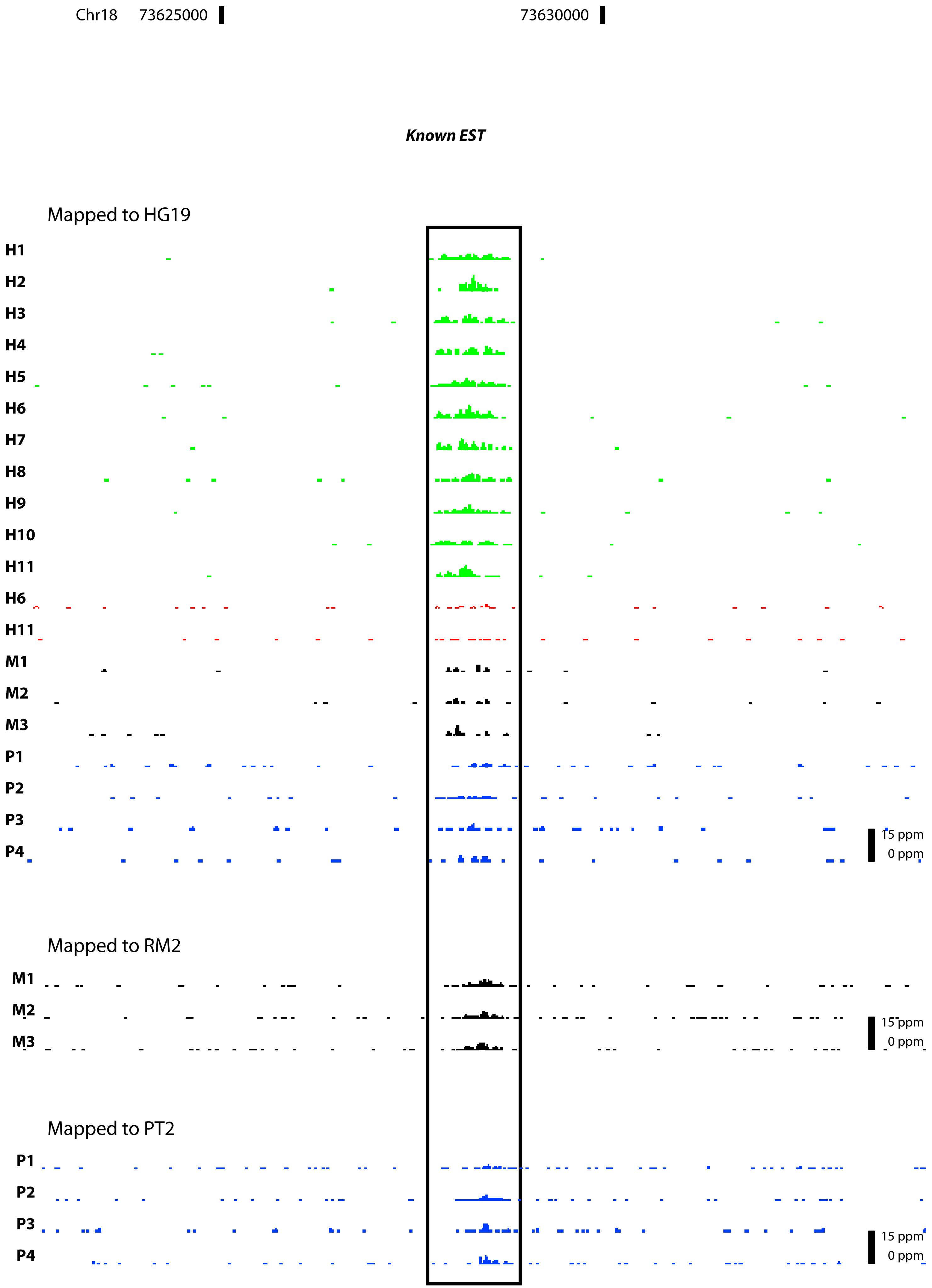

Chr18 73625000
73630000
Known EST
Mapped to HG19
H1
H2
H3
H4
H5
H6
H7
H8
H9
H10
H11
H6
H11
M1
M2
M3
P1
P2
P3
P4
15 ppm
0 ppm
Mapped to RM2
M1
M2
M3
15 ppm
0 ppm
Mapped to PT2
P1
P2
P3
P4
15 ppm
0 ppm

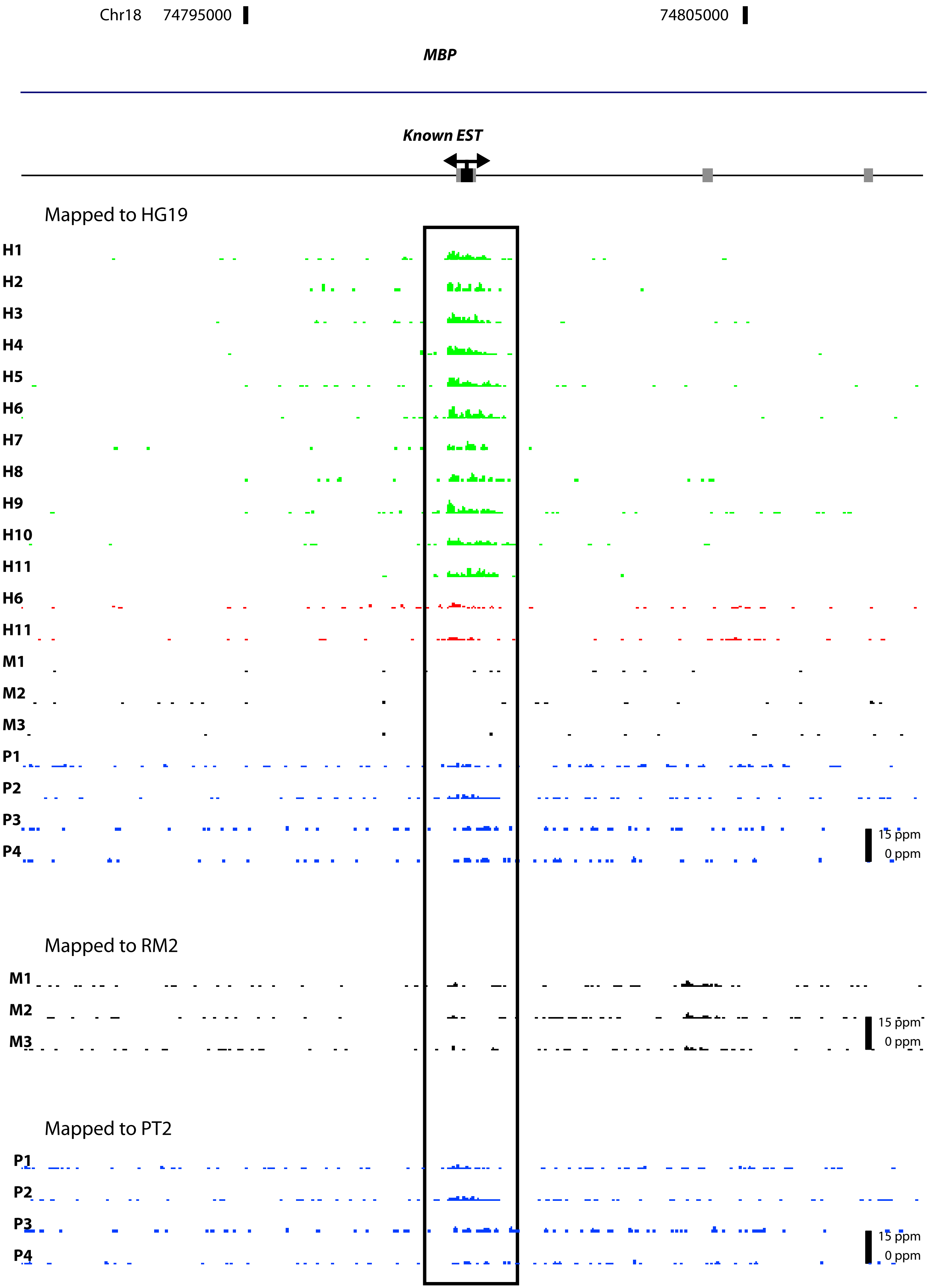

Chr18 74795000
74805000
MBP
Known EST
Mapped to HG19
H1
H2
H3
H4
H5
H6
H7
H8
H9
H10
H11
H6
H11
M1
M2
M3
P1
P2
P3
P4
15 ppm
0 ppm
Mapped to RM2
M1
M2
M3
15 ppm
0 ppm
Mapped to PT2
P1
P2
P3
P4
15 ppm
0 ppm

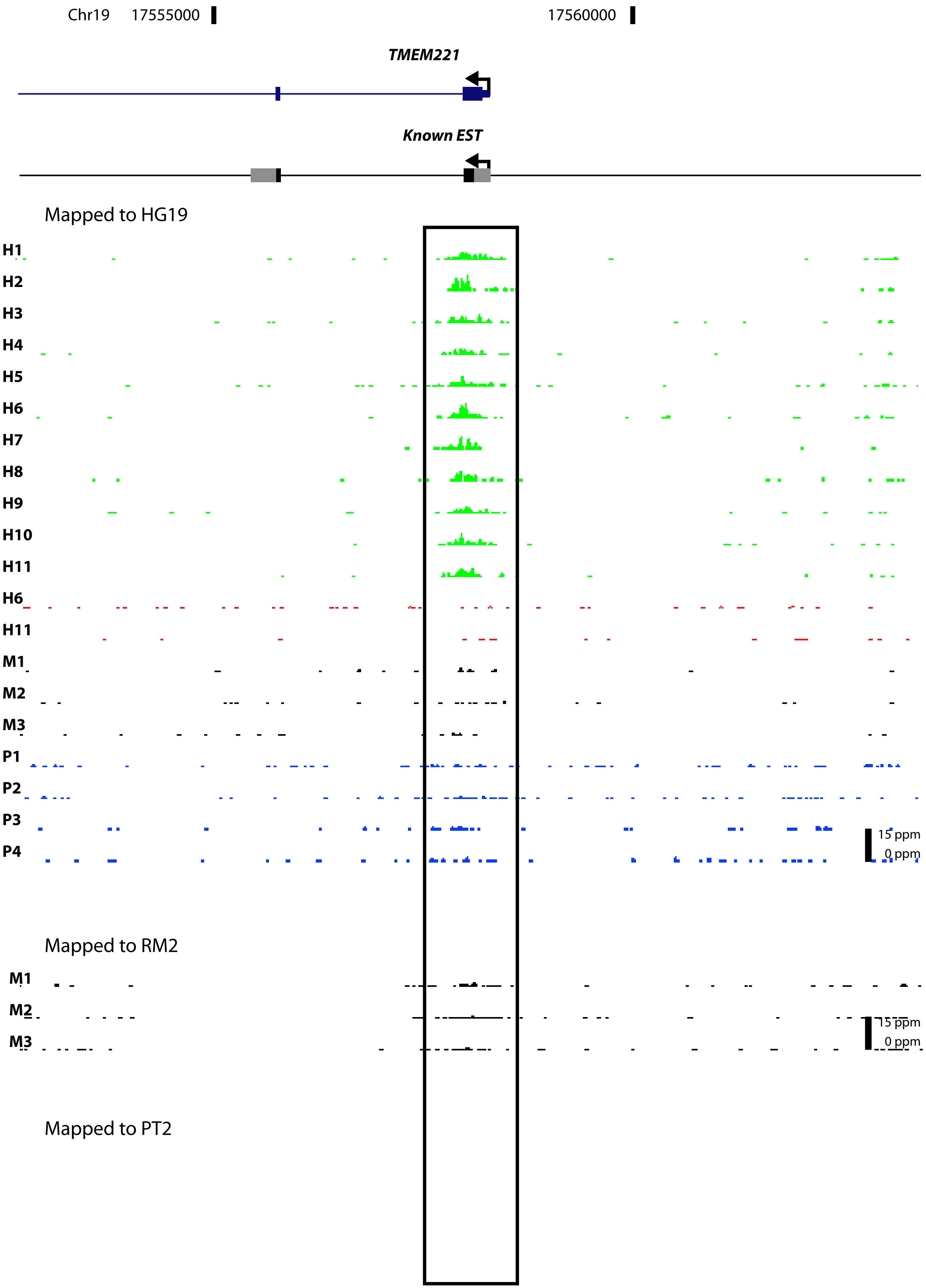

Chr19 17555000
17560000
TMEM221
Known EST
Mapped to HG19
H1
H2
H3
H4
H5
H6
H7
H8
H9
H10
H11
H6
H11
M1
M2
M3
P1
P2
P3
P4
15 ppm
0 ppm
Mapped to RM2
M1
M2
M3
15 ppm
0 ppm
Mapped to PT2

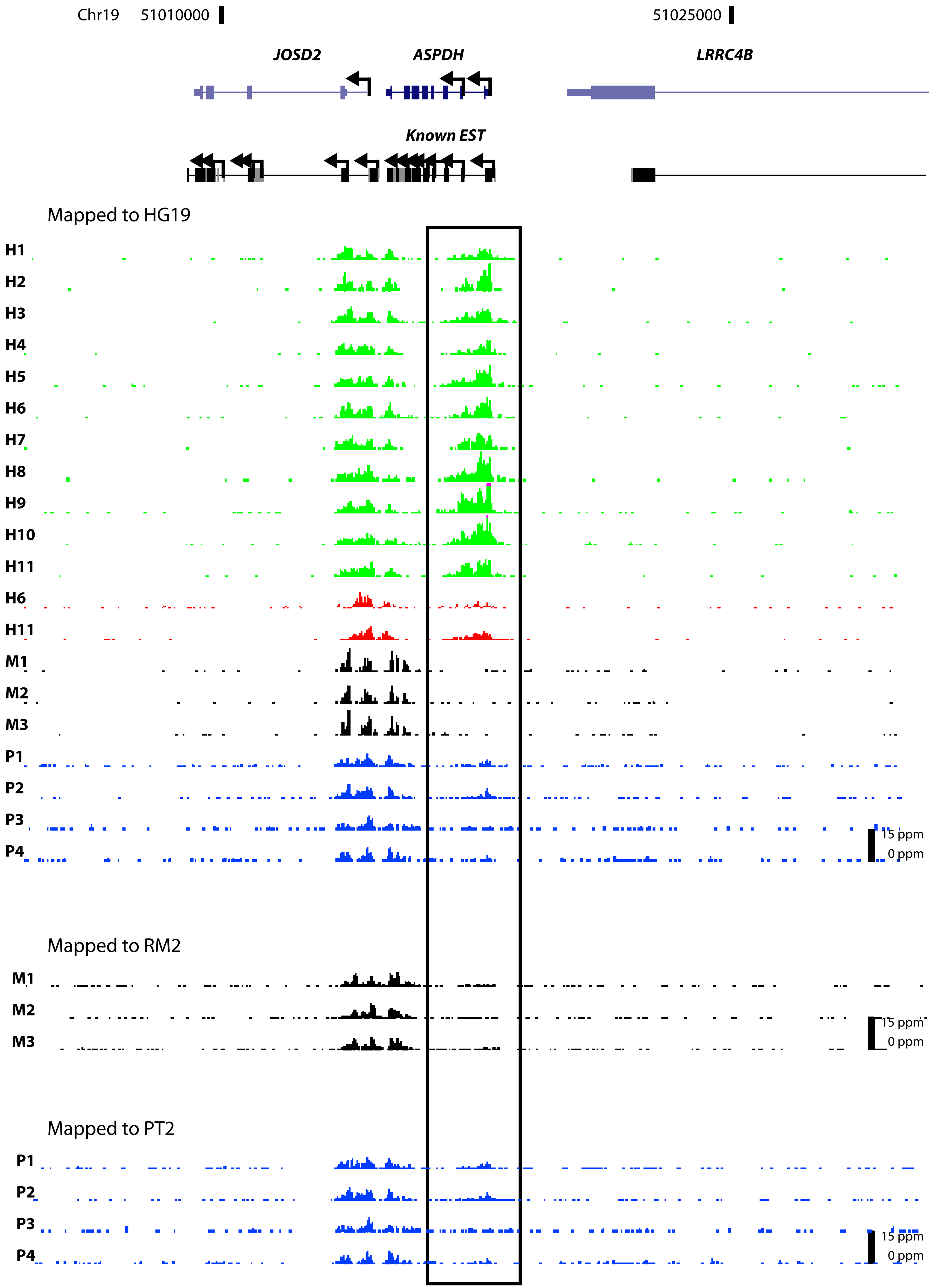

Chr19 51010000
51025000
JOSD2
ASPDH
LRRC4B
Known EST
Mapped to HG19
H1
H2
H3
H4
H5
H6
H7
H8
H9
H10
H11
H6
H11
M1
M2
M3
P1
P2
P3
P4
15 ppm
0 ppm
Mapped to RM2
M1
M2
M3
15 ppm
0 ppm
Mapped to PT2
P1
P2
P3
P4
15 ppm
0 ppm

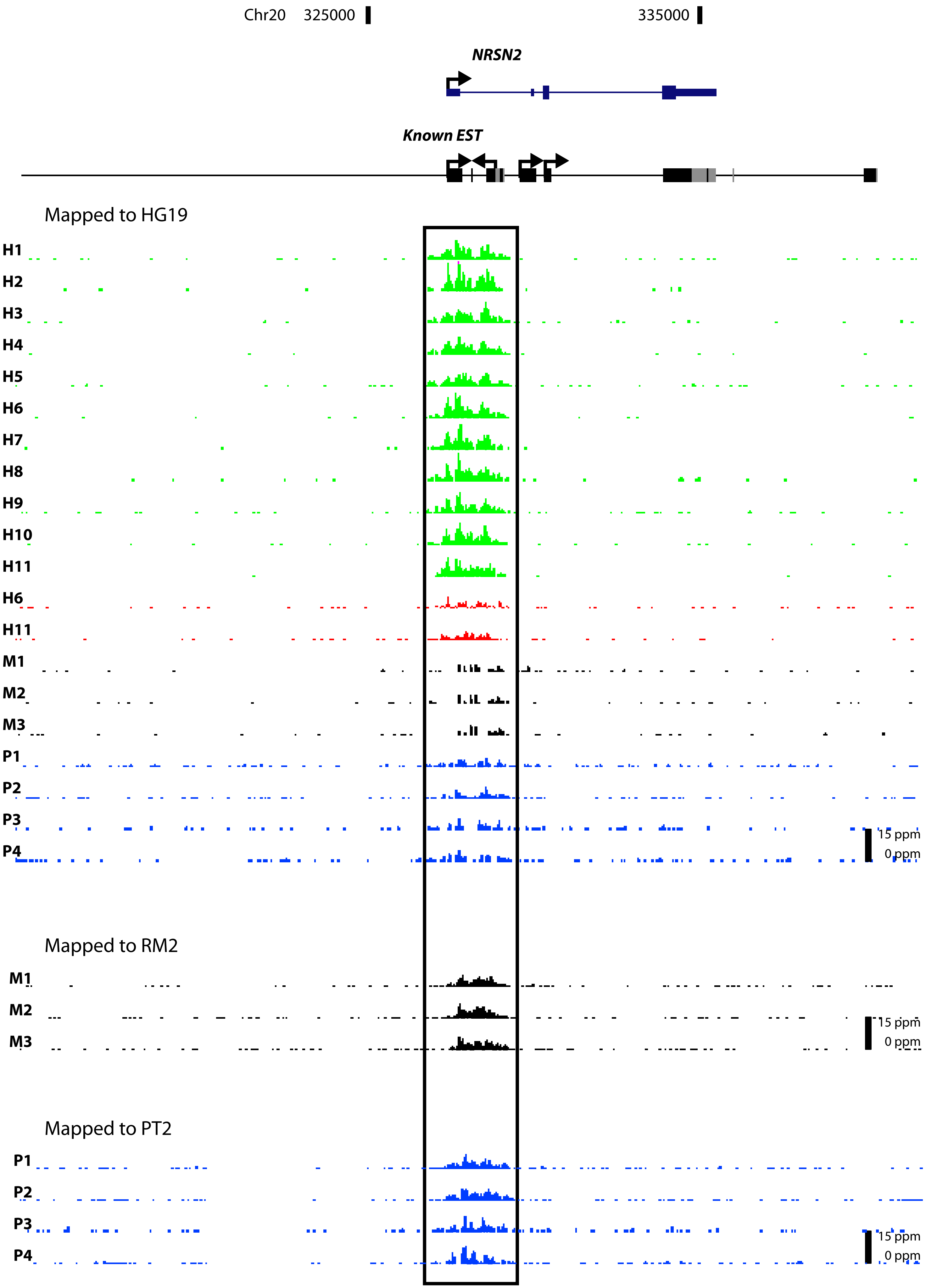

Chr20
325000
335000
NRSN2
Known EST
Mapped to HG19
H1
H2
H3
H4
H5
H6
H7
H8
H9
H10
H11
H6
H11
M1
M2
M3
P1
P2
P3
P4
15 ppm
0 ppm
Mapped to RM2
M1
M2
M3
15 ppm
0 ppm
Mapped to PT2
P1
P2
P3
P4
15 ppm
0 ppm

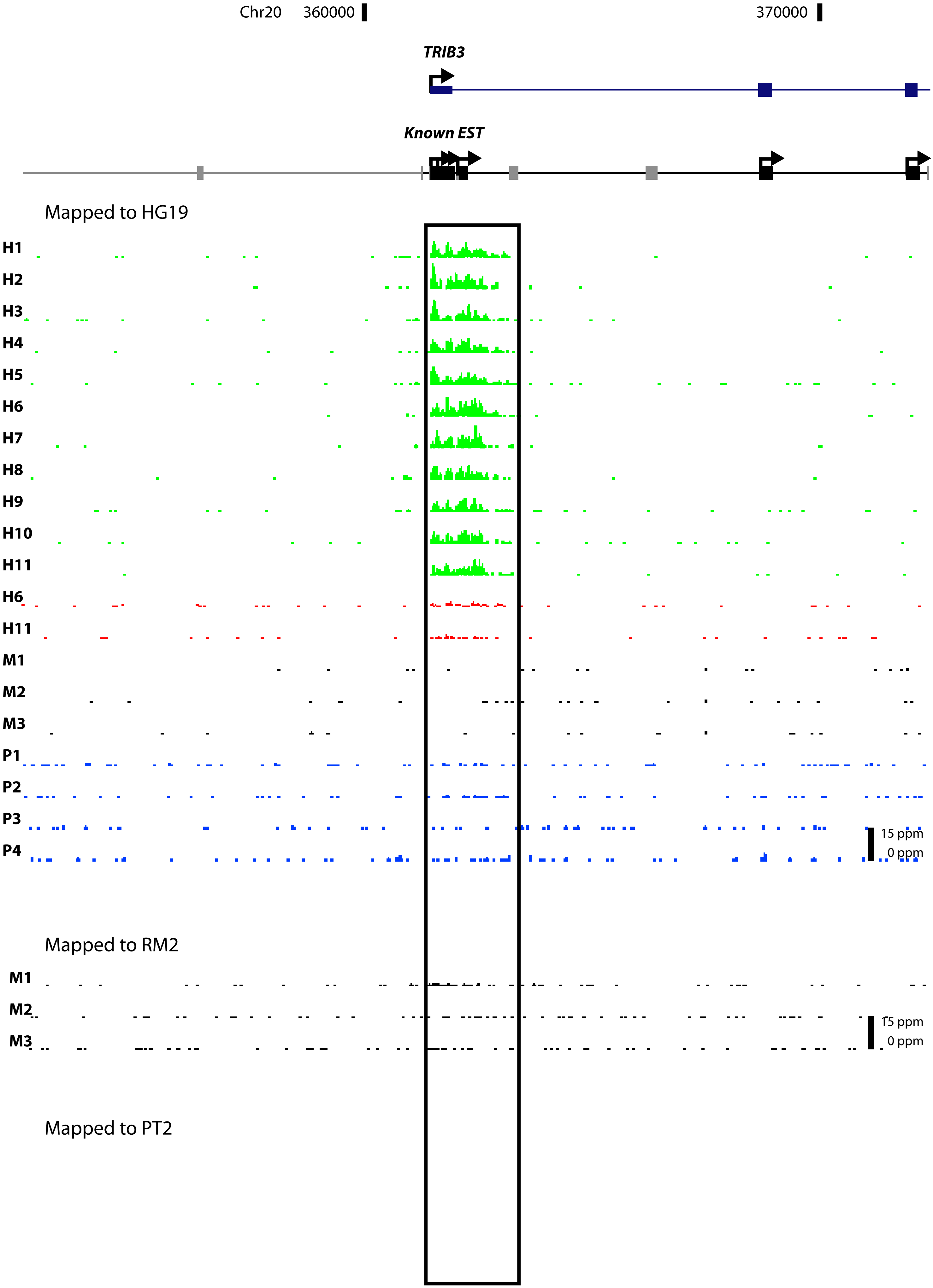

Chr20
360000
370000
TRIB3
Known EST
Mapped to HG19
H1
H2
H3
H4
H5
H6
H7
H8
H9
H10
H11
H6
H11
M1
M2
M3
P1
P2
P3
P4
15 ppm
0 ppm
Mapped to RM2
M1
M2
M3
15 ppm
0 ppm
Mapped to PT2

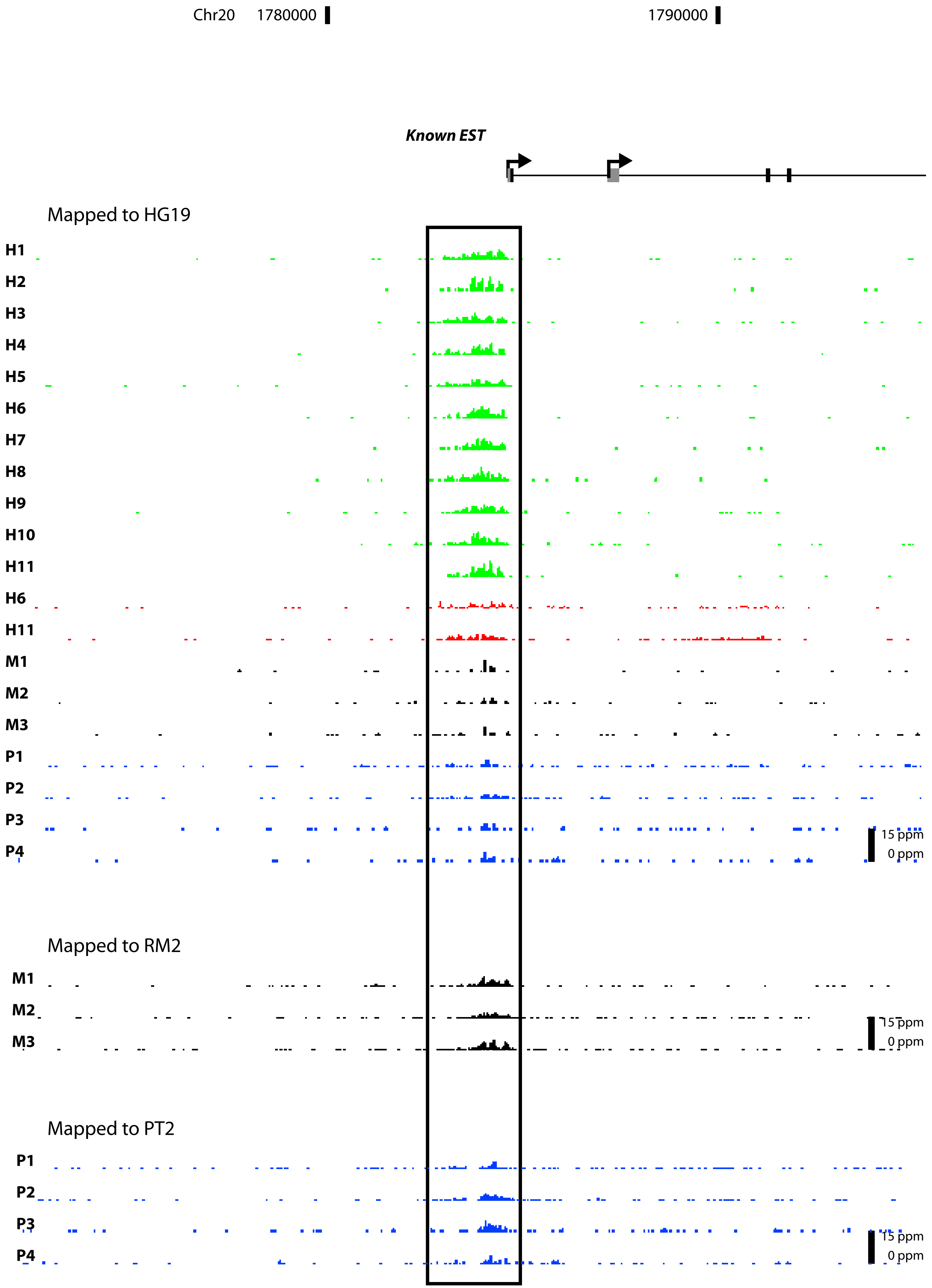

Chr20
1780000
1790000
Known EST
Mapped to HG19
H1
H2
H3
H4
H5
H6
H7
H8
H9
H10
H11
H6
H11
M1
M2
M3
P1
P2
P3
P4
15 ppm
0 ppm
Mapped to RM2
M1
M2
M3
15 ppm
0 ppm
Mapped to PT2
P1
P2
P3
P4
15 ppm
0 ppm

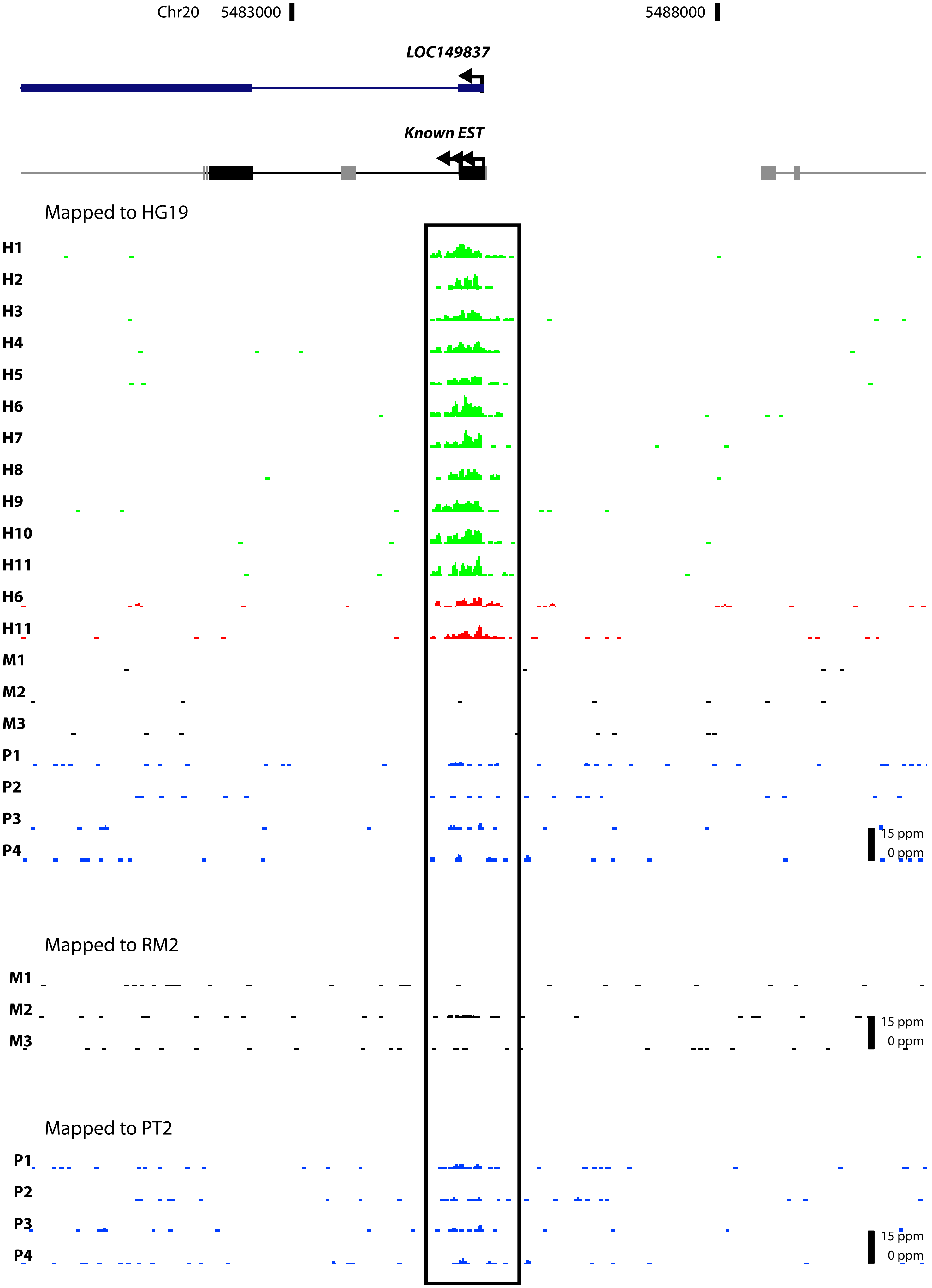

Chr20
5483000
5488000
LOC149837
Known EST
Mapped to HG19
H1
H2
H3
H4
H5
H6
H7
H8
H9
H10
H11
H6
H11
M1
M2
M3
P1
P2
P3
P4
15 ppm
0 ppm
Mapped to RM2
M1
M2
M3
15 ppm
0 ppm
Mapped to PT2
P1
P2
P3
P4
15 ppm
0 ppm

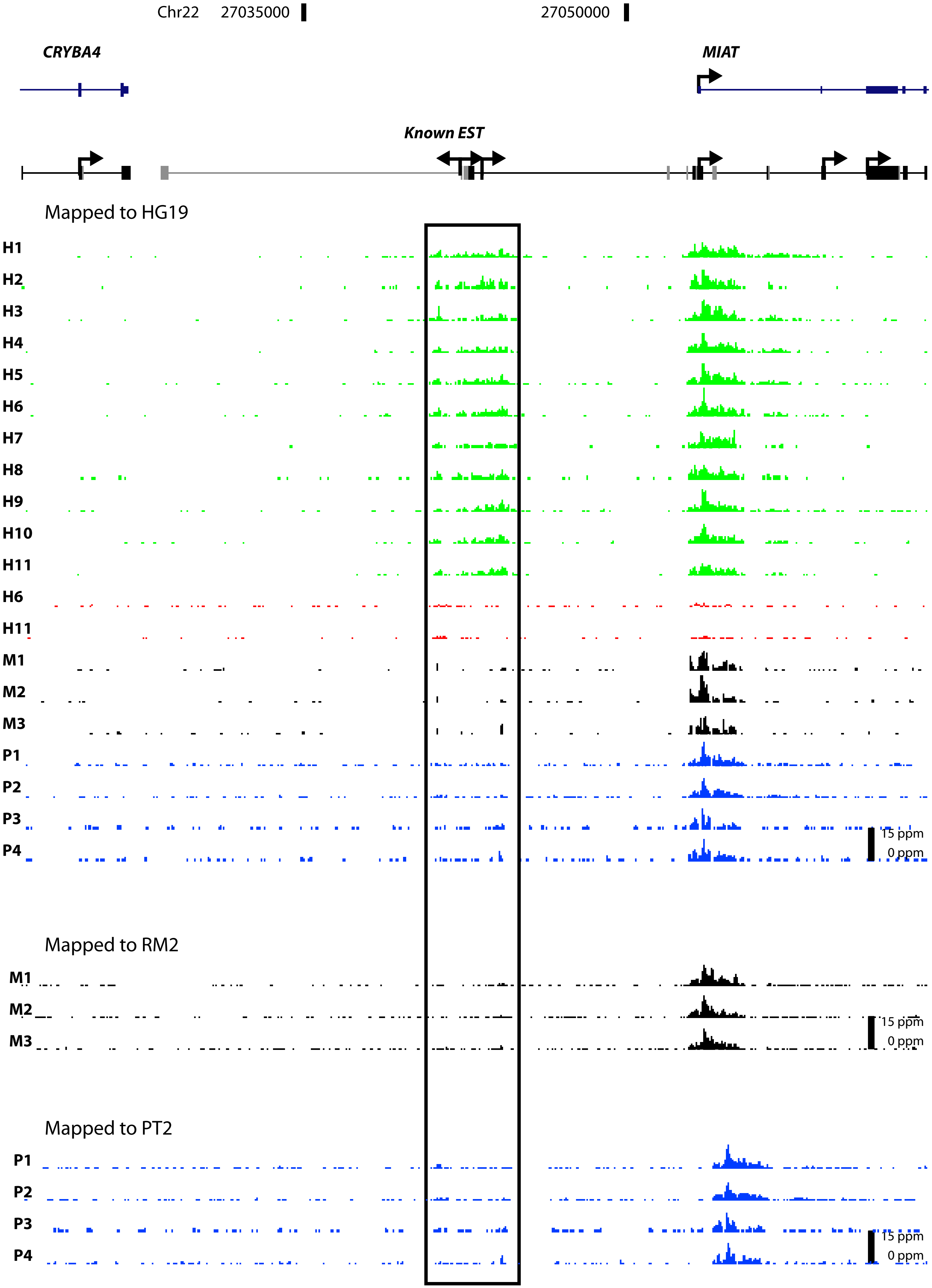

Chr22
27035000
27050000
CRYBA4
MIAT
Known EST
Mapped to HG19
H1
H2
H3
H4
H5
H6
H7
H8
H9
H10
H11
H6
H11
M1
M2
M3
P1
P2
P3
P4
15 ppm
0 ppm
Mapped to RM2
M1
M2
M3
15 ppm
0 ppm
Mapped to PT2
P1
P2
P3
P4
15 ppm
0 ppm

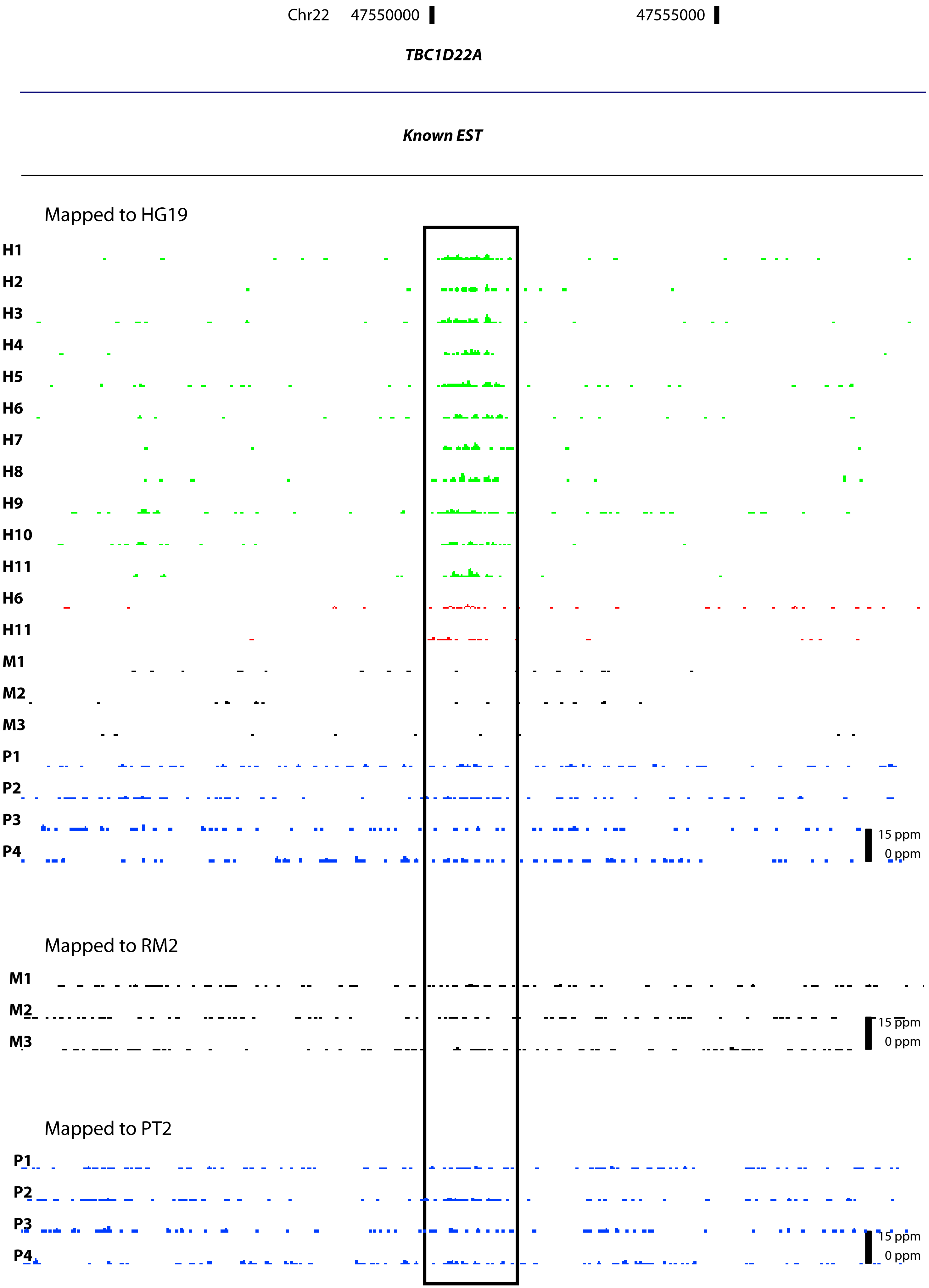

Chr22 47550000
47555000
TBC1D22A
Known EST
Mapped to HG19
H1
H2
H3
H4
H5
H6
H7
H8
H9
H10
H11
H6
H11
M1
M2
M3
P1
P2
P3
P4
15 ppm
0 ppm
Mapped to RM2
M1
M2
M3
15 ppm
0 ppm
Mapped to PT2
P1
P2
P3
P4
15 ppm
0 ppm
