# Supplementary material for: Human-Specific Histone Methylation Signatures at Transcription Start Sites in Prefrontal Neurons
Source: PLoS Biol. 2012 Nov 20;10(11):e1001427. doi: 10.1371/journal.pbio.1001427 (PMC3502543; doi:10.1371/journal.pbio.1001427)
Supplement: Figure S2 — RNAseq tag densities in human, chimpanzee, and macaque PFC for H3K4me3 peaks shown in Figure S1. (PDF) [file pbio.1001427.s002.pdf]

Supplementary Figure 2 (Shulha et al):

UCSC genome browser tracks showing RNA sequence tags in human (orange), macaque (green) and chimpanzee (brown) prefrontal cortex in region surrounding the human-specific H3K4me3 peak (shown as black bar, top). Human tracks represent original data, data for macaque (chimpanzee) RNA expression from GSE24538 (GSE30352).

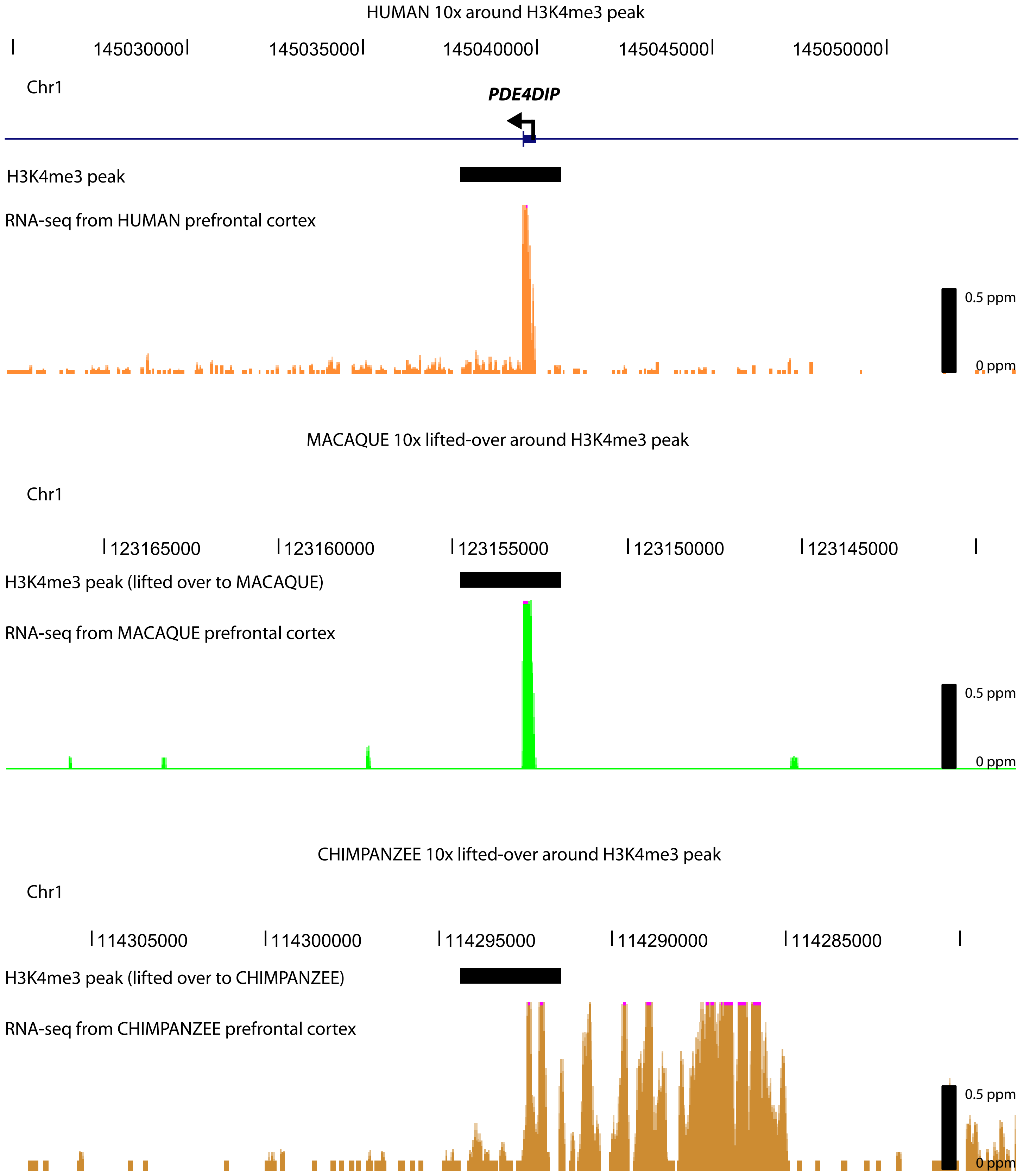
HUMAN 10x around H3K4me3 peak
145030000
145035000
145040000
145045000
145050000
Chr1
PDE4DIP
H3K4me3 peak
RNA-seq from HUMAN prefrontal cortex
0.5 ppm
0 ppm
MACAQUE 10x lifted-over around H3K4me3 peak
Chr1
123165000
123160000
123155000
123150000
123145000
H3K4me3 peak (lifted over to MACAQUE)
RNA-seq from MACAQUE prefrontal cortex
0.5 ppm
0 ppm
CHIMPANZEE 10x lifted-over around H3K4me3 peak
Chr1
114305000
114300000
114295000
114290000
114285000
H3K4me3 peak (lifted over to CHIMPANZEE)
RNA-seq from CHIMPANZEE prefrontal cortex
0.5 ppm
0 ppm

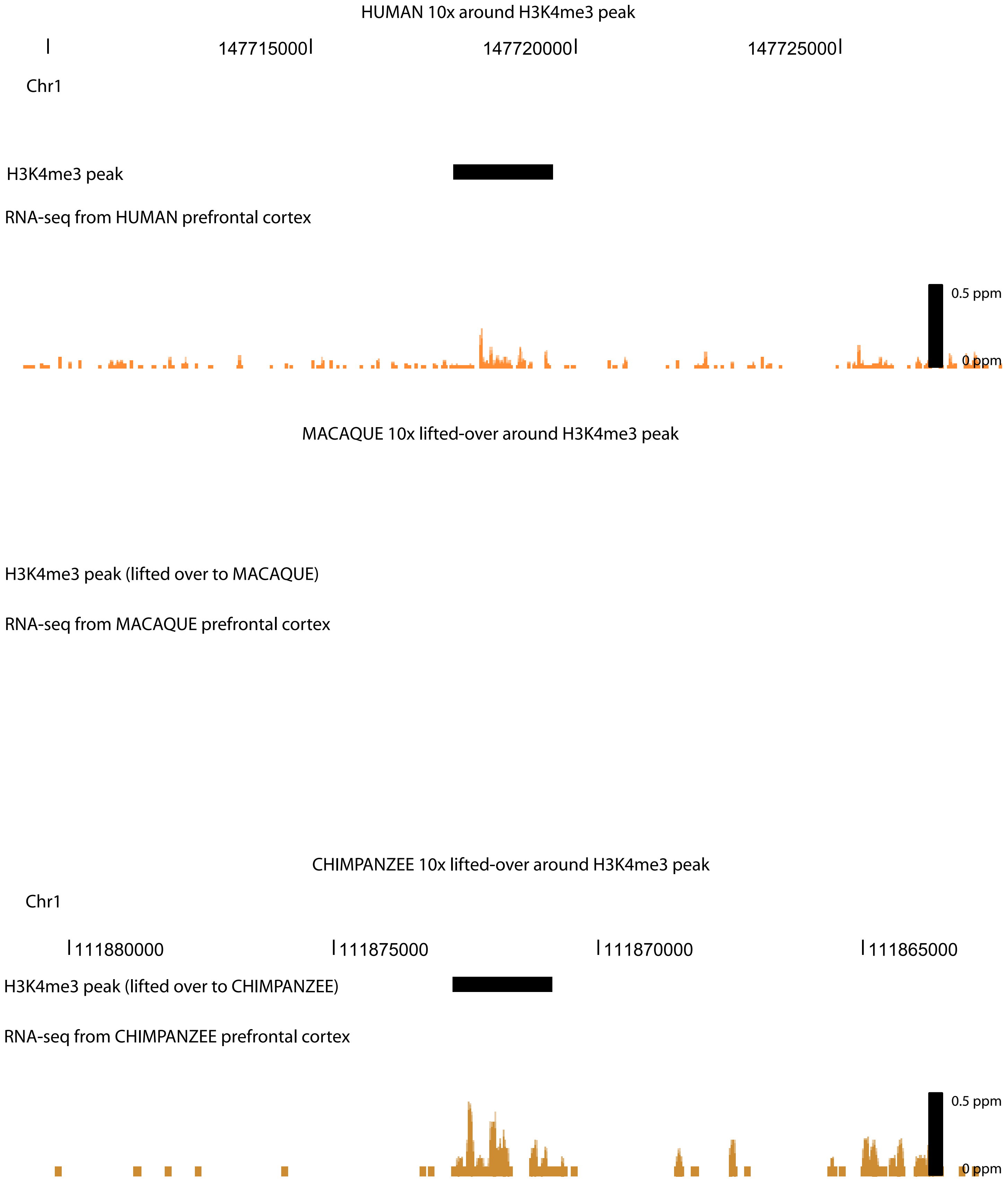
HUMAN 10x around H3K4me3 peak
147715000
147720000
147725000
Chr1
H3K4me3 peak
RNA-seq from HUMAN prefrontal cortex
0.5 ppm
0 ppm
MACAQUE 10x lifted-over around H3K4me3 peak
H3K4me3 peak (lifted over to MACAQUE)
RNA-seq from MACAQUE prefrontal cortex
CHIMPANZEE 10x lifted-over around H3K4me3 peak
Chr1
111880000
111875000
111870000
111865000
H3K4me3 peak (lifted over to CHIMPANZEE)
RNA-seq from CHIMPANZEE prefrontal cortex
0.5 ppm
0 ppm

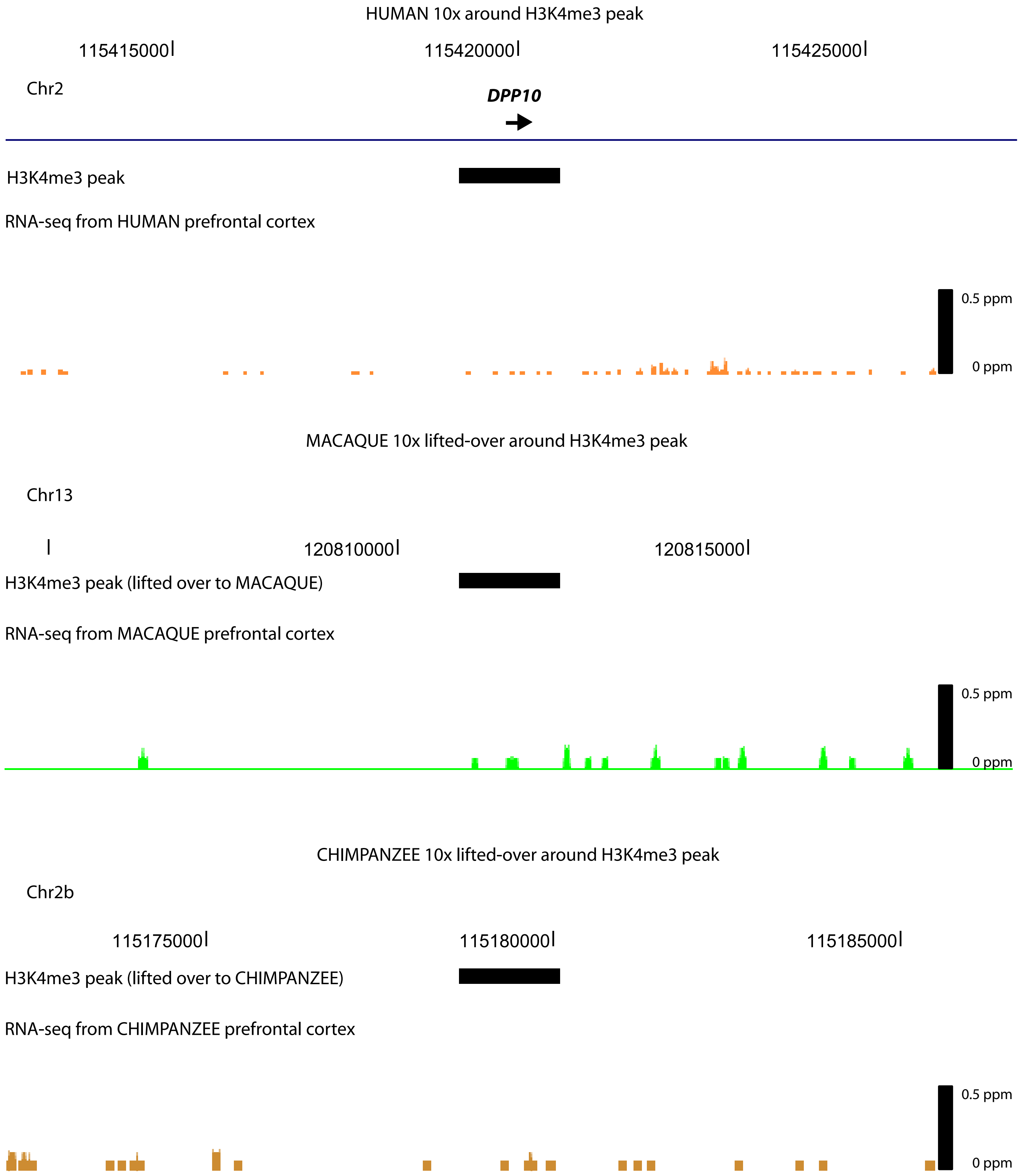

HUMAN 10x around H3K4me3 peak
115415000
115420000
115425000
Chr2
DPP10
H3K4me3 peak
RNA-seq from HUMAN prefrontal cortex
0.5 ppm
0 ppm
MACAQUE 10x lifted-over around H3K4me3 peak
Chr13
120810000
120815000
H3K4me3 peak (lifted over to MACAQUE)
RNA-seq from MACAQUE prefrontal cortex
0.5 ppm
0 ppm
CHIMPANZEE 10x lifted-over around H3K4me3 peak
Chr2b
115175000
115180000
115185000
H3K4me3 peak (lifted over to CHIMPANZEE)
RNA-seq from CHIMPANZEE prefrontal cortex
0.5 ppm
0 ppm

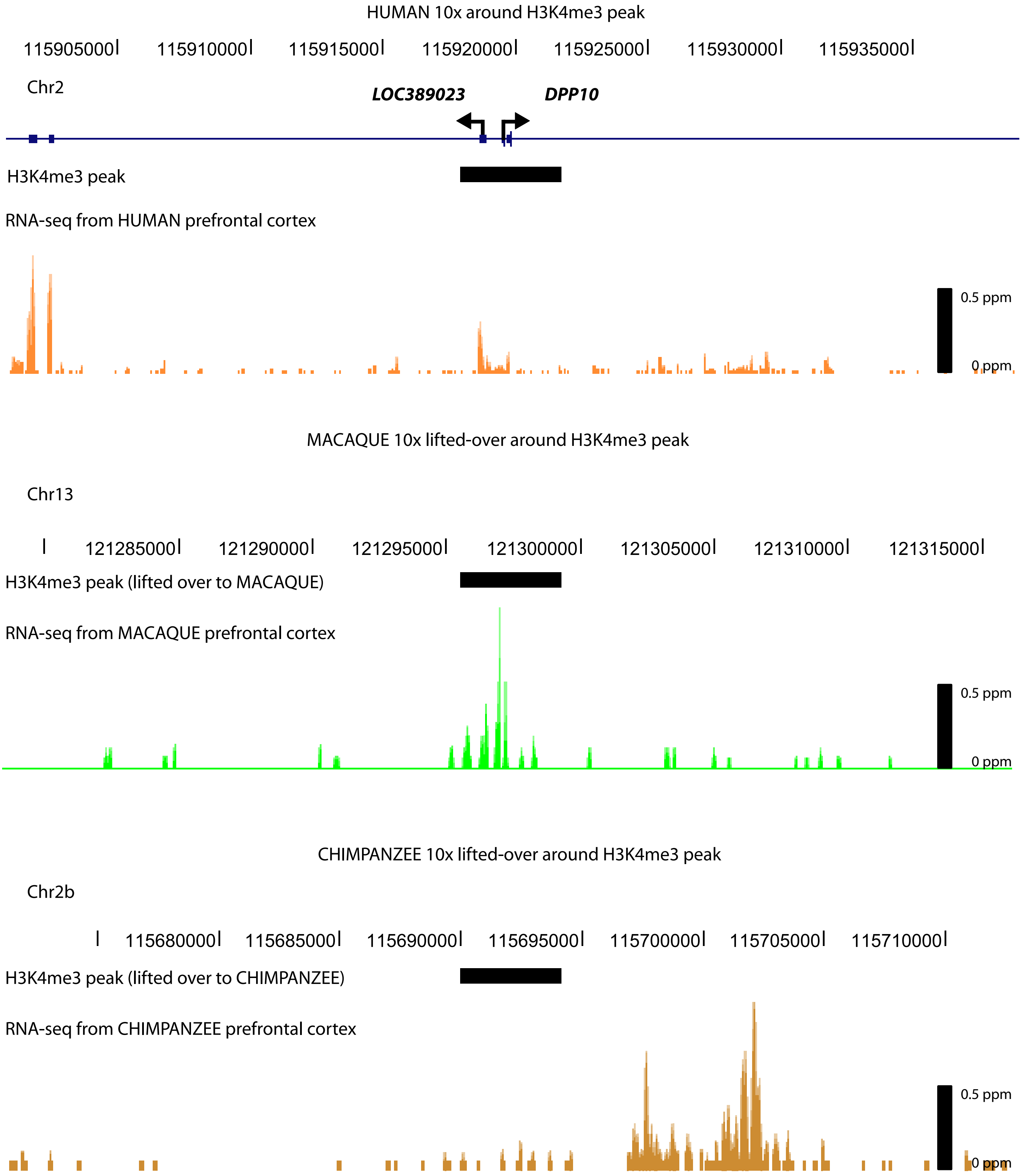
HUMAN 10x around H3K4me3 peak
115905000
115910000
115915000
115920000
115925000
115930000
115935000
Chr2
LOC389023
DPP10
H3K4me3 peak
RNA-seq from HUMAN prefrontal cortex
0.5 ppm
0 ppm
MACAQUE 10x lifted-over around H3K4me3 peak
Chr13
121285000
121290000
121295000
121300000
121305000
121310000
121315000
H3K4me3 peak (lifted over to MACAQUE)
RNA-seq from MACAQUE prefrontal cortex
0.5 ppm
0 ppm
CHIMPANZEE 10x lifted-over around H3K4me3 peak
Chr2b
115680000
115685000
115690000
115695000
115700000
115705000
115710000
H3K4me3 peak (lifted over to CHIMPANZEE)
RNA-seq from CHIMPANZEE prefrontal cortex
0.5 ppm
0 ppm

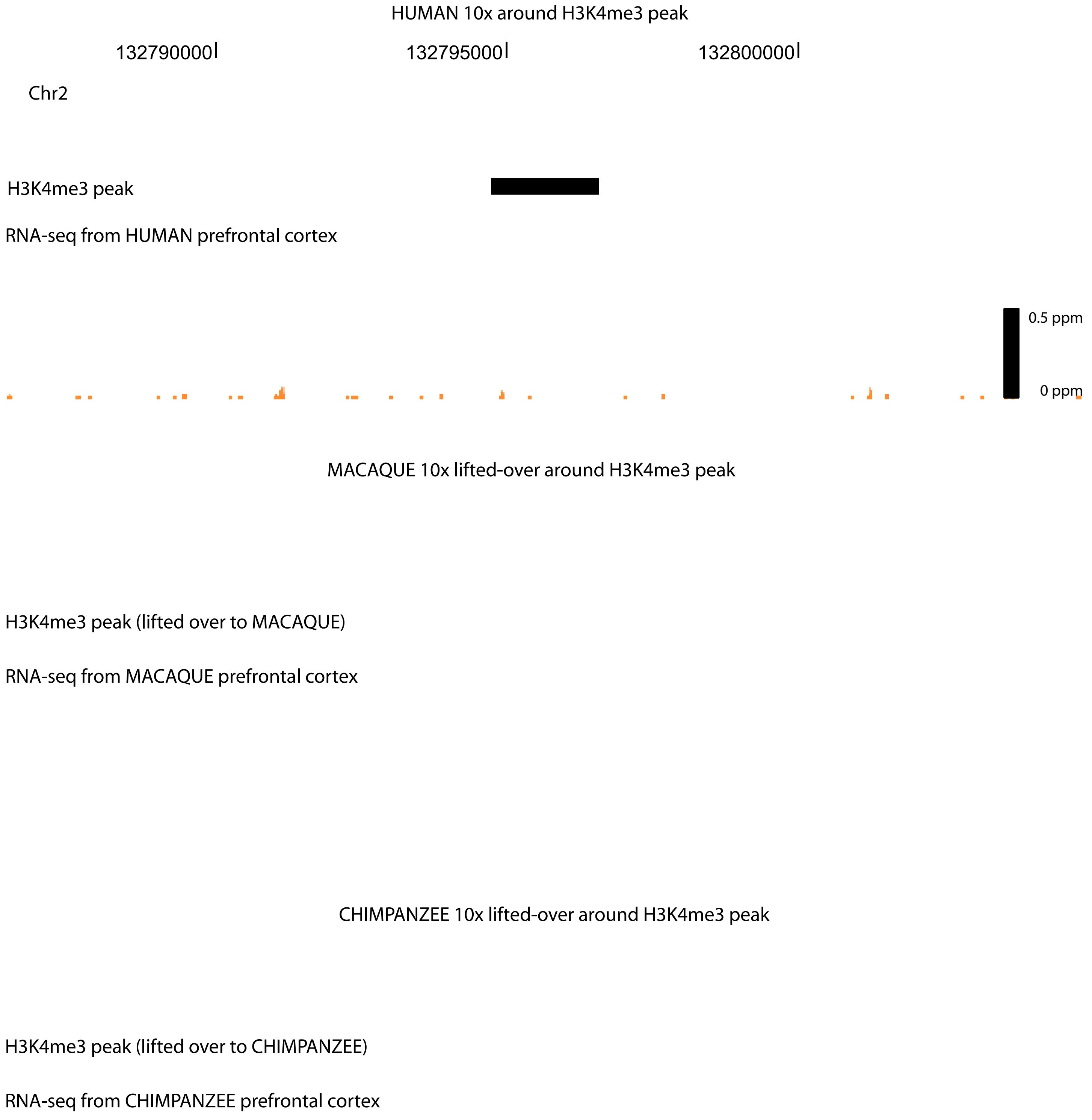
HUMAN 10x around H3K4me3 peak
132790000
132795000
132800000
Chr2
H3K4me3 peak
RNA-seq from HUMAN prefrontal cortex
0.5 ppm
0 ppm
MACAQUE 10x lifted-over around H3K4me3 peak
H3K4me3 peak (lifted over to MACAQUE)
RNA-seq from MACAQUE prefrontal cortex
CHIMPANZEE 10x lifted-over around H3K4me3 peak
H3K4me3 peak (lifted over to CHIMPANZEE)
RNA-seq from CHIMPANZEE prefrontal cortex

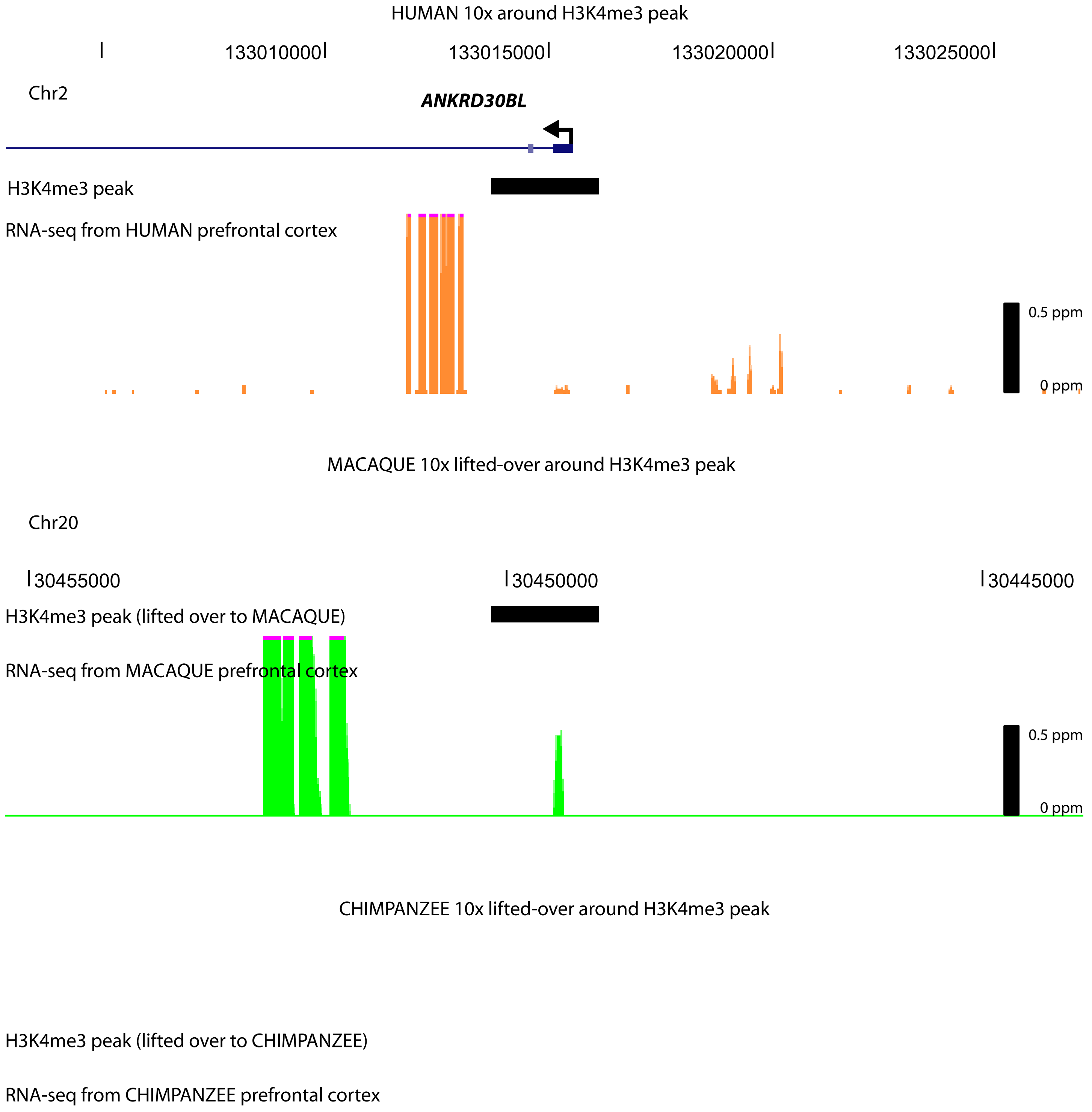

HUMAN 10x around H3K4me3 peak
133010000
133015000
133020000
133025000
Chr2
ANKRD30BL
H3K4me3 peak
RNA-seq from HUMAN prefrontal cortex
0.5 ppm
0 ppm
MACAQUE 10x lifted-over around H3K4me3 peak
Chr20
30455000
30450000
30445000
H3K4me3 peak (lifted over to MACAQUE)
RNA-seq from MACAQUE prefrontal cortex
0.5 ppm
0 ppm
CHIMPANZEE 10x lifted-over around H3K4me3 peak
H3K4me3 peak (lifted over to CHIMPANZEE)
RNA-seq from CHIMPANZEE prefrontal cortex

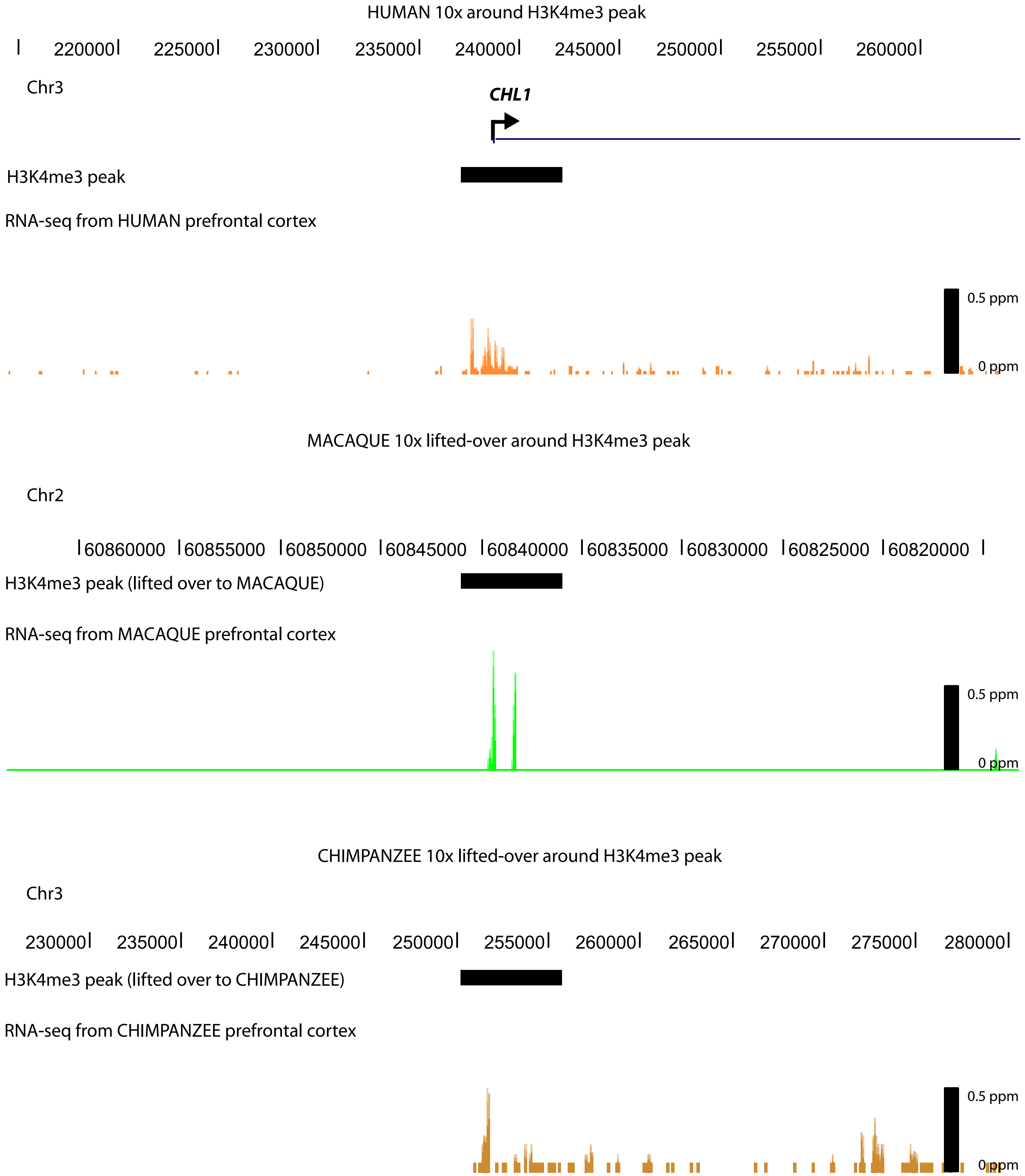
HUMAN 10x around H3K4me3 peak
220000 225000 230000 235000 240000 245000 250000 255000 260000
Chr3
CHL1
H3K4me3 peak
RNA-seq from HUMAN prefrontal cortex
0.5 ppm
0 ppm
MACAQUE 10x lifted-over around H3K4me3 peak
Chr2
60860000 60855000 60850000 60845000 60840000 60835000 60830000 60825000 60820000
H3K4me3 peak (lifted over to MACAQUE)
RNA-seq from MACAQUE prefrontal cortex
0.5 ppm
0 ppm
CHIMPANZEE 10x lifted-over around H3K4me3 peak
Chr3
230000 235000 240000 245000 250000 255000 260000 265000 270000 275000 280000
H3K4me3 peak (lifted over to CHIMPANZEE)
RNA-seq from CHIMPANZEE prefrontal cortex
0.5 ppm
0 ppm

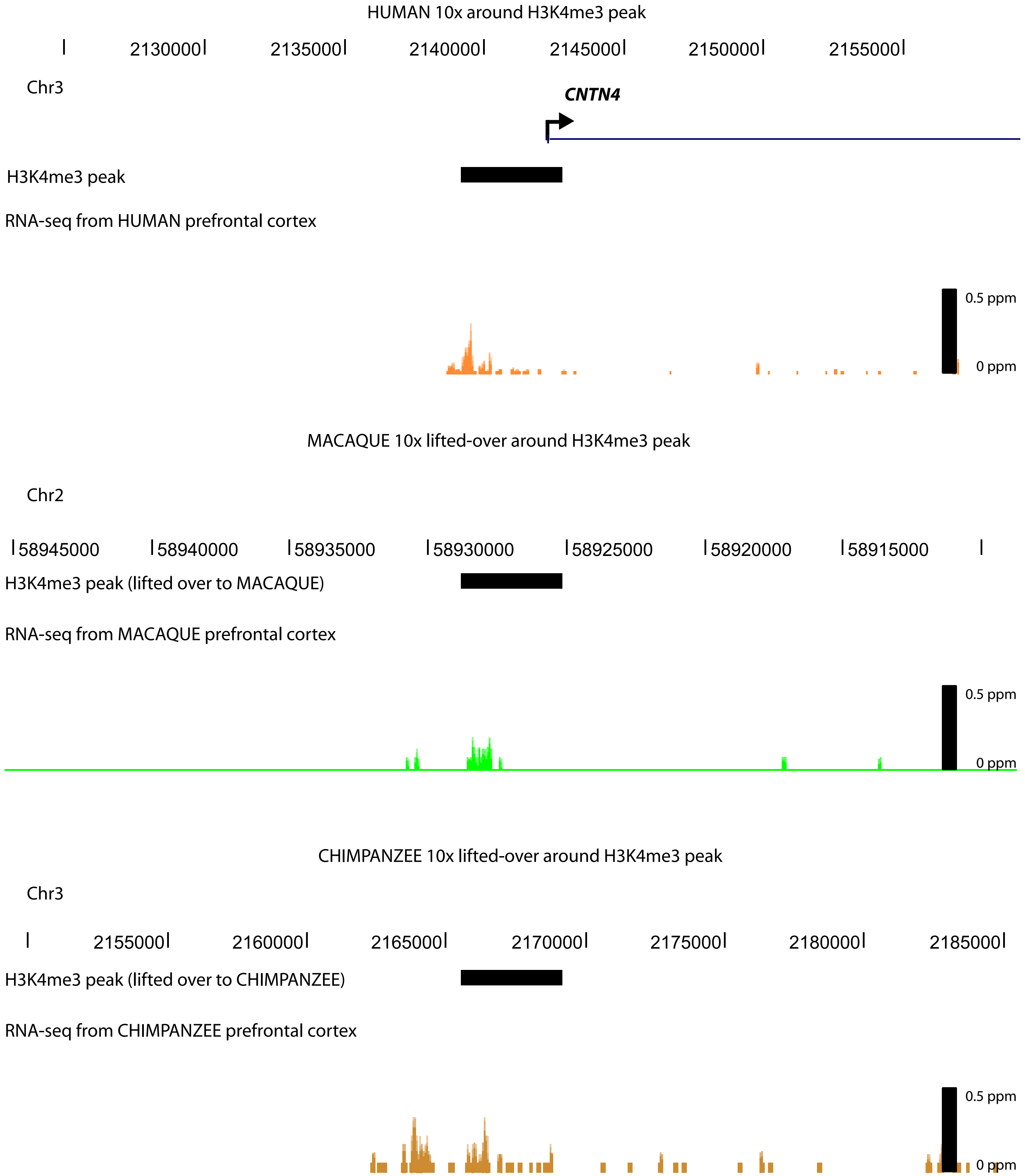
HUMAN 10x around H3K4me3 peak
2130000
2135000
2140000
2145000
2150000
2155000
Chr3
CNTN4
H3K4me3 peak
RNA-seq from HUMAN prefrontal cortex
0.5 ppm
0 ppm
MACAQUE 10x lifted-over around H3K4me3 peak
Chr2
58945000
58940000
58935000
58930000
58925000
58920000
58915000
H3K4me3 peak (lifted over to MACAQUE)
RNA-seq from MACAQUE prefrontal cortex
0.5 ppm
0 ppm
CHIMPANZEE 10x lifted-over around H3K4me3 peak
Chr3
2155000
2160000
2165000
2170000
2175000
2180000
2185000
H3K4me3 peak (lifted over to CHIMPANZEE)
RNA-seq from CHIMPANZEE prefrontal cortex
0.5 ppm
0 ppm

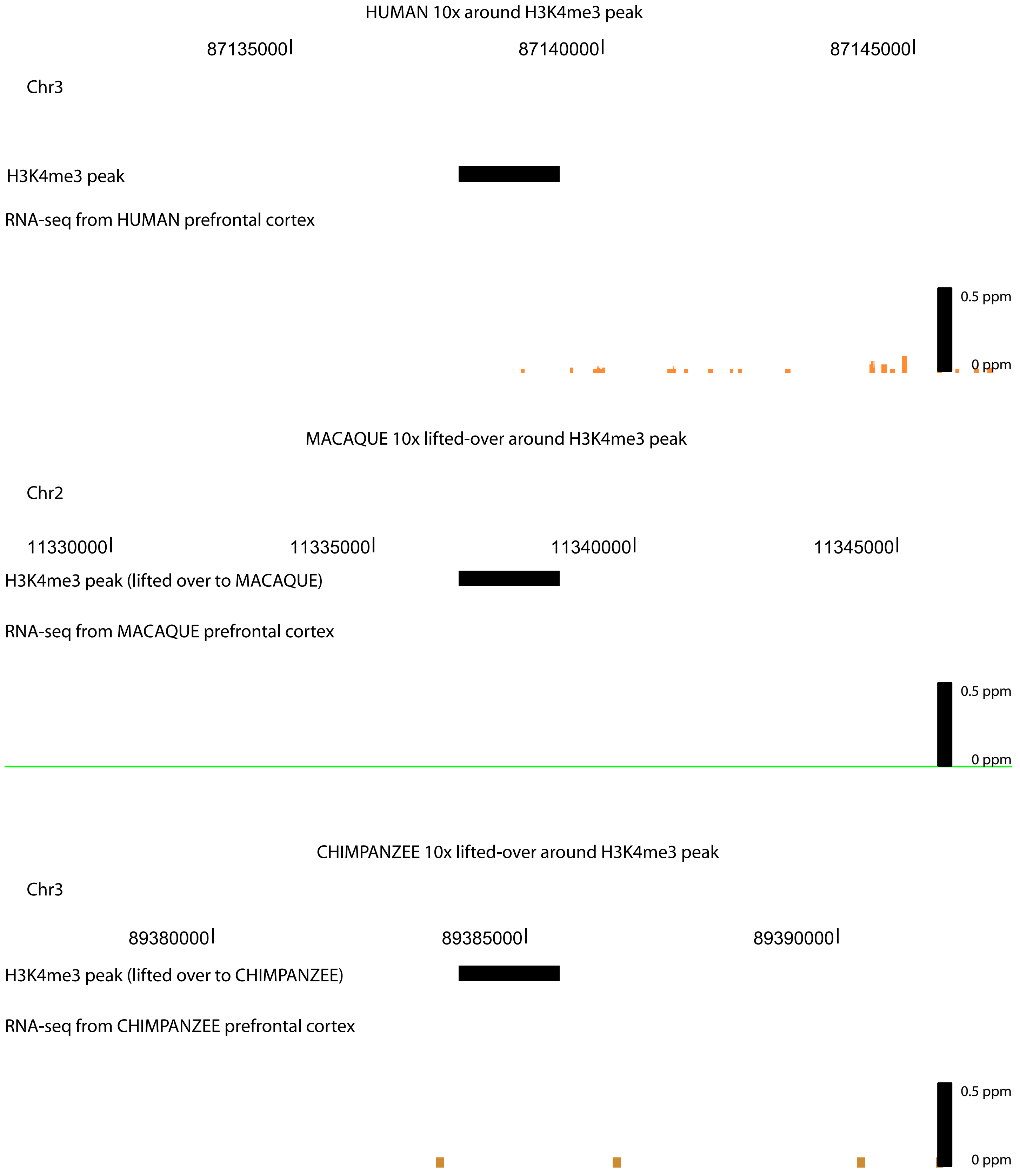
HUMAN 10x around H3K4me3 peak
87135000
87140000
87145000
Chr3
H3K4me3 peak
RNA-seq from HUMAN prefrontal cortex
0.5 ppm
0 ppm
MACAQUE 10x lifted-over around H3K4me3 peak
Chr2
11330000
11335000
11340000
11345000
H3K4me3 peak (lifted over to MACAQUE)
RNA-seq from MACAQUE prefrontal cortex
0.5 ppm
0 ppm
CHIMPANZEE 10x lifted-over around H3K4me3 peak
Chr3
89380000
89385000
89390000
H3K4me3 peak (lifted over to CHIMPANZEE)
RNA-seq from CHIMPANZEE prefrontal cortex
0.5 ppm
0 ppm

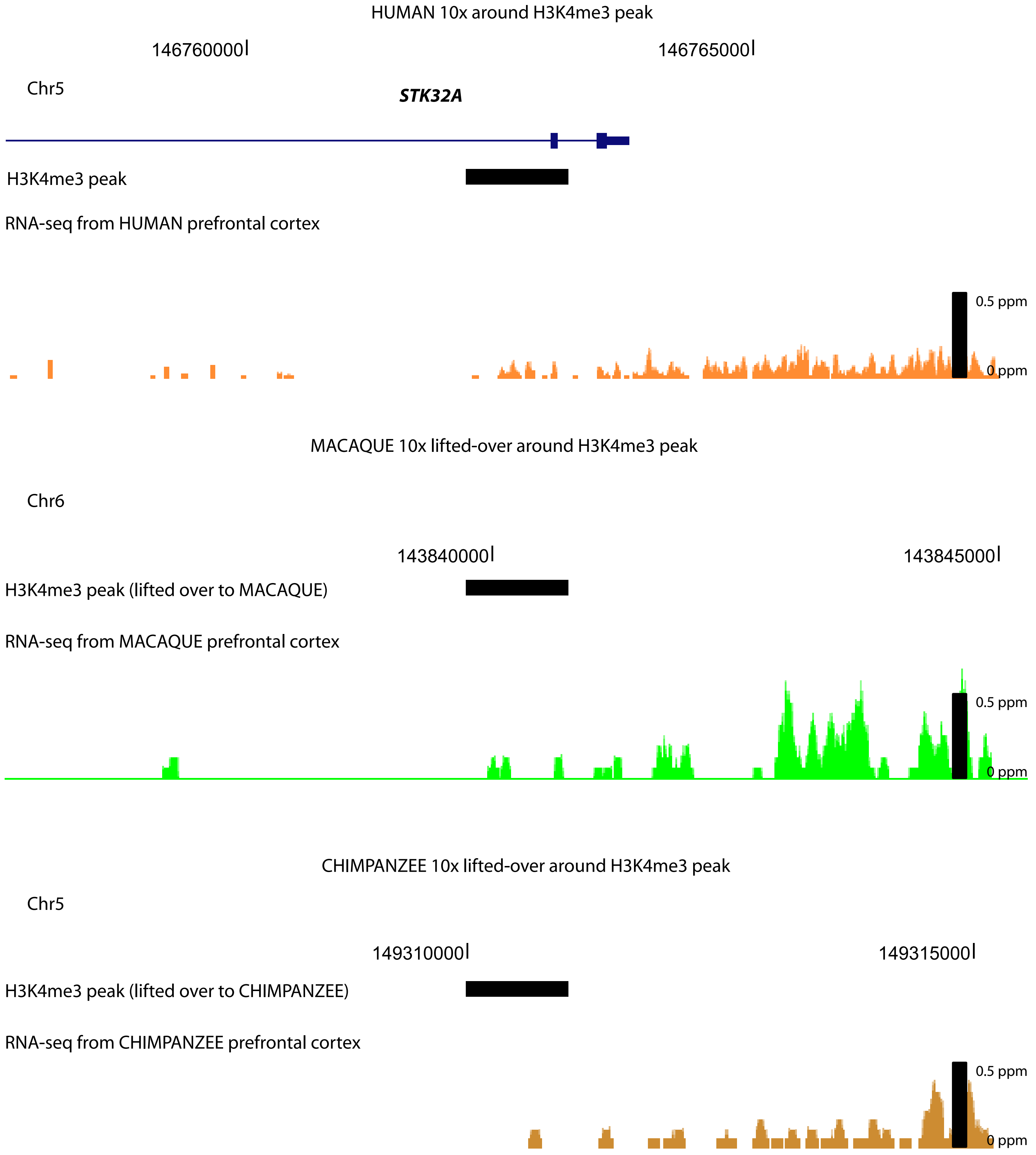
HUMAN 10x around H3K4me3 peak
146760000
146765000
Chr5
STK32A
H3K4me3 peak
RNA-seq from HUMAN prefrontal cortex
0.5 ppm
0 ppm
MACAQUE 10x lifted-over around H3K4me3 peak
Chr6
143840000
143845000
H3K4me3 peak (lifted over to MACAQUE)
RNA-seq from MACAQUE prefrontal cortex
0.5 ppm
0 ppm
CHIMPANZEE 10x lifted-over around H3K4me3 peak
Chr5
149310000
149315000
H3K4me3 peak (lifted over to CHIMPANZEE)
RNA-seq from CHIMPANZEE prefrontal cortex
0.5 ppm
0 ppm

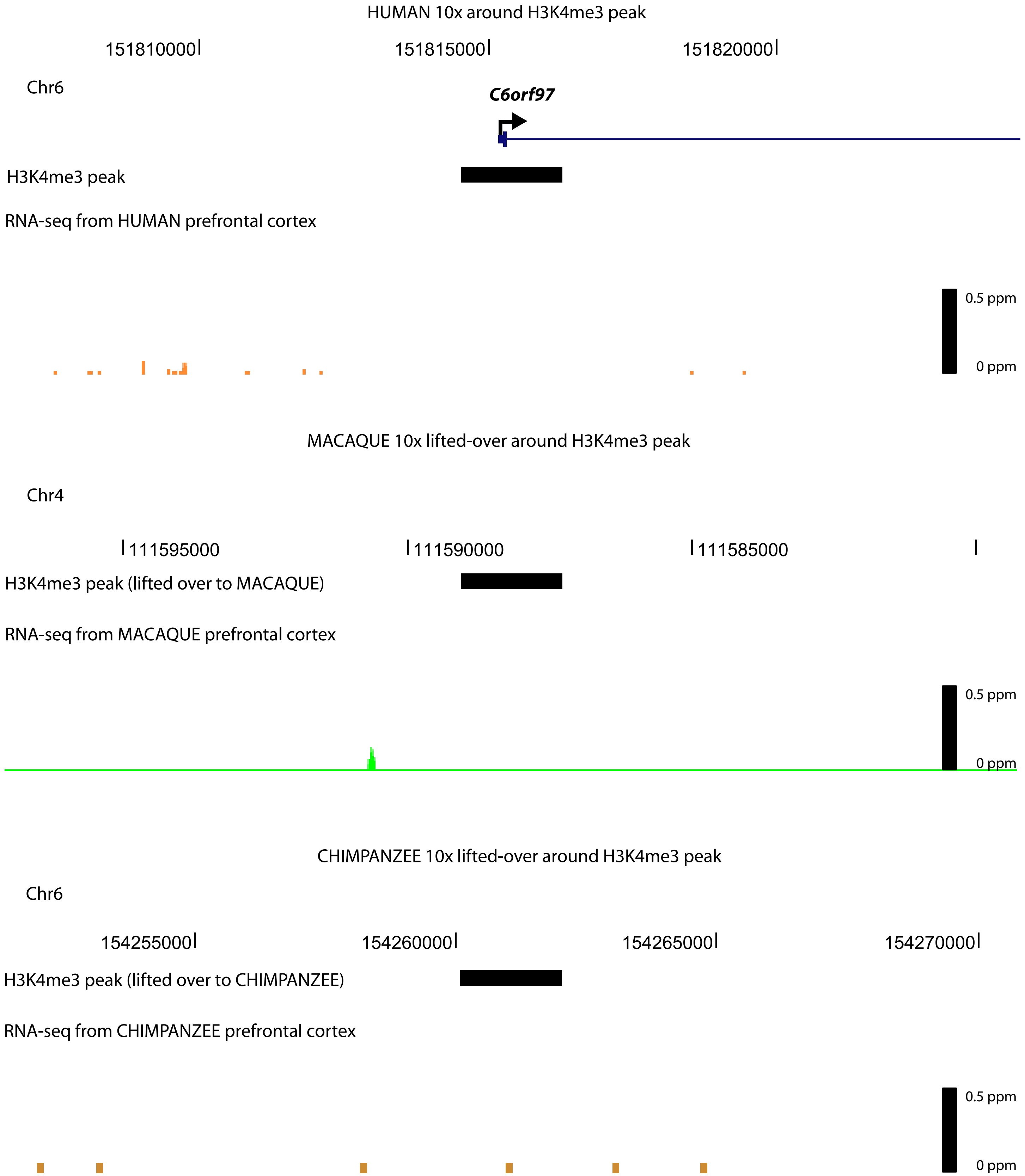
HUMAN 10x around H3K4me3 peak
151810000
151815000
151820000
Chr6
C6orf97
H3K4me3 peak
RNA-seq from HUMAN prefrontal cortex
0.5 ppm
0 ppm
MACAQUE 10x lifted-over around H3K4me3 peak
Chr4
111595000
111590000
111585000
H3K4me3 peak (lifted over to MACAQUE)
RNA-seq from MACAQUE prefrontal cortex
0.5 ppm
0 ppm
CHIMPANZEE 10x lifted-over around H3K4me3 peak
Chr6
154255000
154260000
154265000
154270000
H3K4me3 peak (lifted over to CHIMPANZEE)
RNA-seq from CHIMPANZEE prefrontal cortex
0.5 ppm
0 ppm

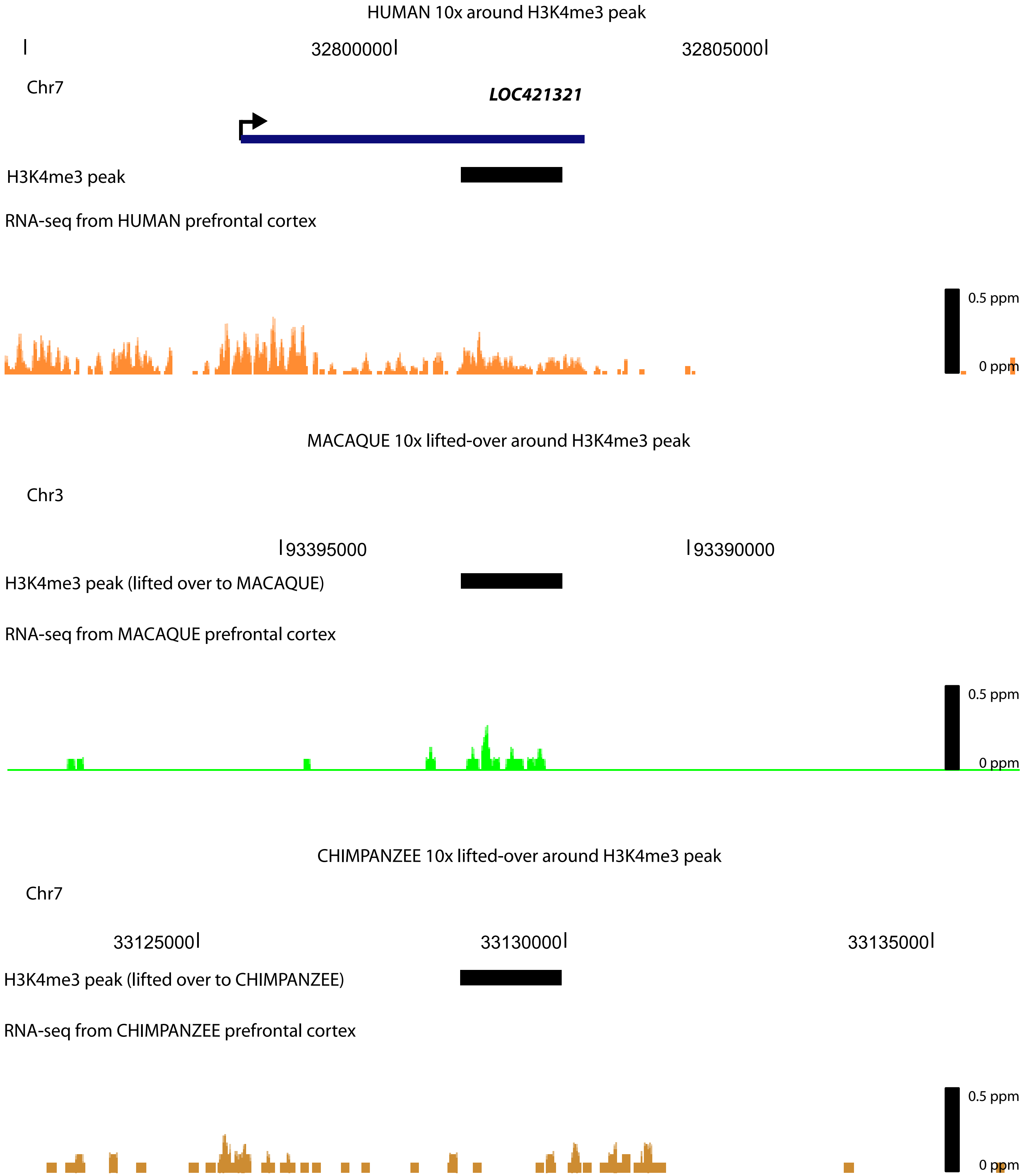
HUMAN 10x around H3K4me3 peak
32800000
32805000
Chr7
LOC421321
H3K4me3 peak
RNA-seq from HUMAN prefrontal cortex
0.5 ppm
0 ppm
MACAQUE 10x lifted-over around H3K4me3 peak
Chr3
93395000
93390000
H3K4me3 peak (lifted over to MACAQUE)
RNA-seq from MACAQUE prefrontal cortex
0.5 ppm
0 ppm
CHIMPANZEE 10x lifted-over around H3K4me3 peak
Chr7
33125000
33130000
33135000
H3K4me3 peak (lifted over to CHIMPANZEE)
RNA-seq from CHIMPANZEE prefrontal cortex
0.5 ppm
0 ppm

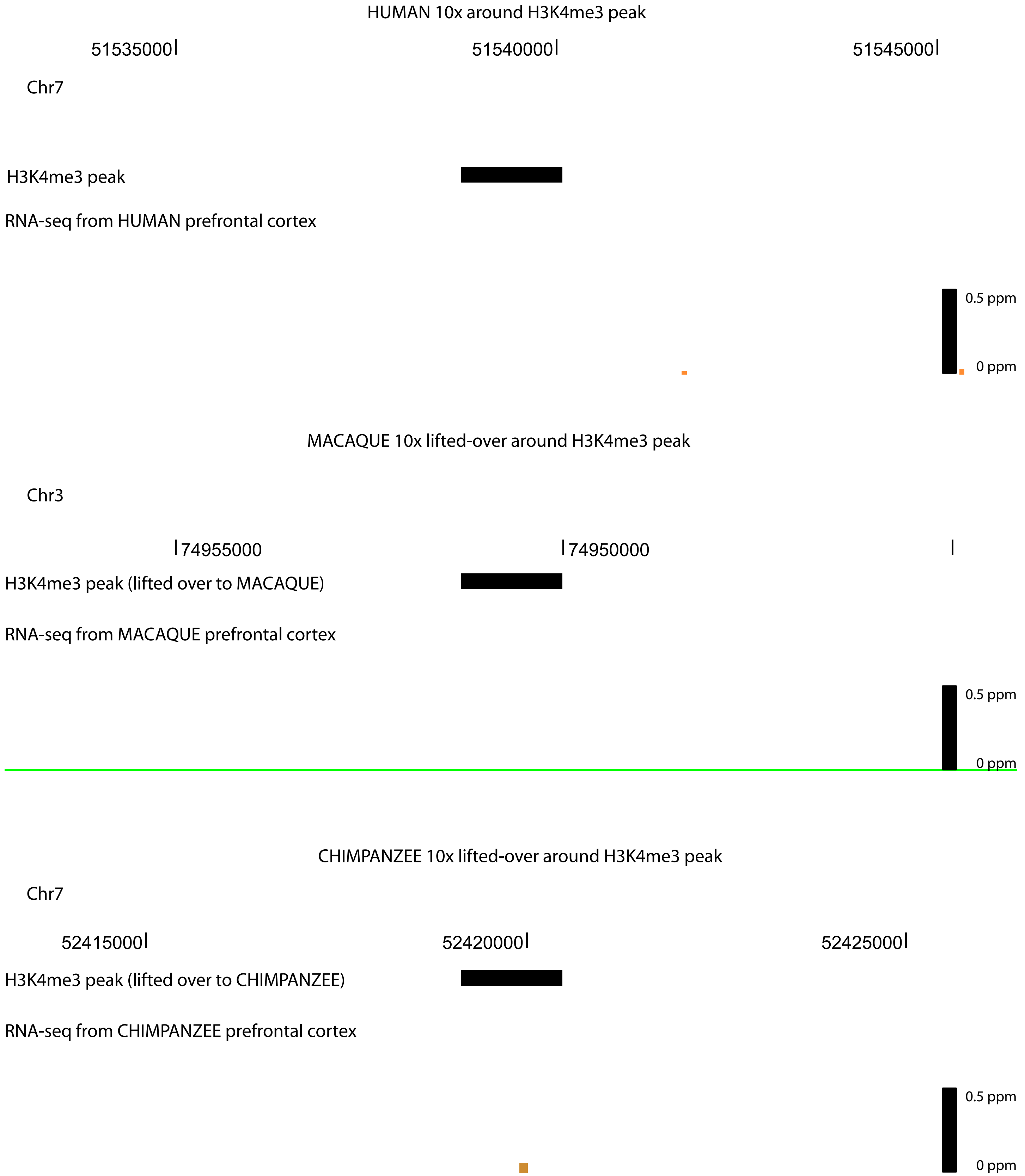
HUMAN 10x around H3K4me3 peak
51535000
51540000
51545000
Chr7
H3K4me3 peak
RNA-seq from HUMAN prefrontal cortex
0.5 ppm
0 ppm
MACAQUE 10x lifted-over around H3K4me3 peak
Chr3
74955000
74950000
H3K4me3 peak (lifted over to MACAQUE)
RNA-seq from MACAQUE prefrontal cortex
0.5 ppm
0 ppm
CHIMPANZEE 10x lifted-over around H3K4me3 peak
Chr7
52415000
52420000
52425000
H3K4me3 peak (lifted over to CHIMPANZEE)
RNA-seq from CHIMPANZEE prefrontal cortex
0.5 ppm
0 ppm

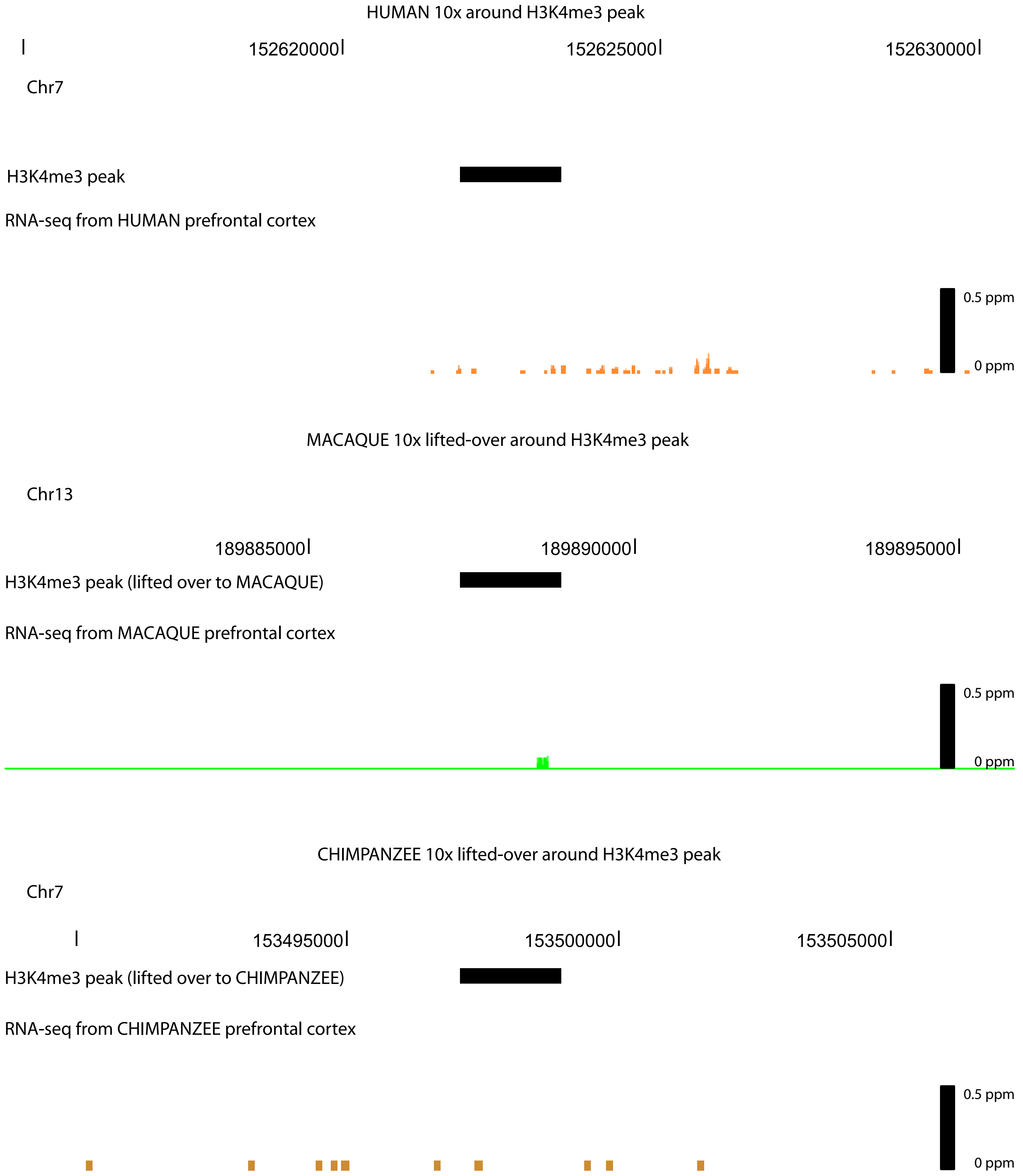

HUMAN 10x around H3K4me3 peak
152620000
152625000
152630000
Chr7
H3K4me3 peak
RNA-seq from HUMAN prefrontal cortex
0.5 ppm
0 ppm
MACAQUE 10x lifted-over around H3K4me3 peak
Chr13
189885000
189890000
189895000
H3K4me3 peak (lifted over to MACAQUE)
RNA-seq from MACAQUE prefrontal cortex
0.5 ppm
0 ppm
CHIMPANZEE 10x lifted-over around H3K4me3 peak
Chr7
153495000
153500000
153505000
H3K4me3 peak (lifted over to CHIMPANZEE)
RNA-seq from CHIMPANZEE prefrontal cortex
0.5 ppm
0 ppm

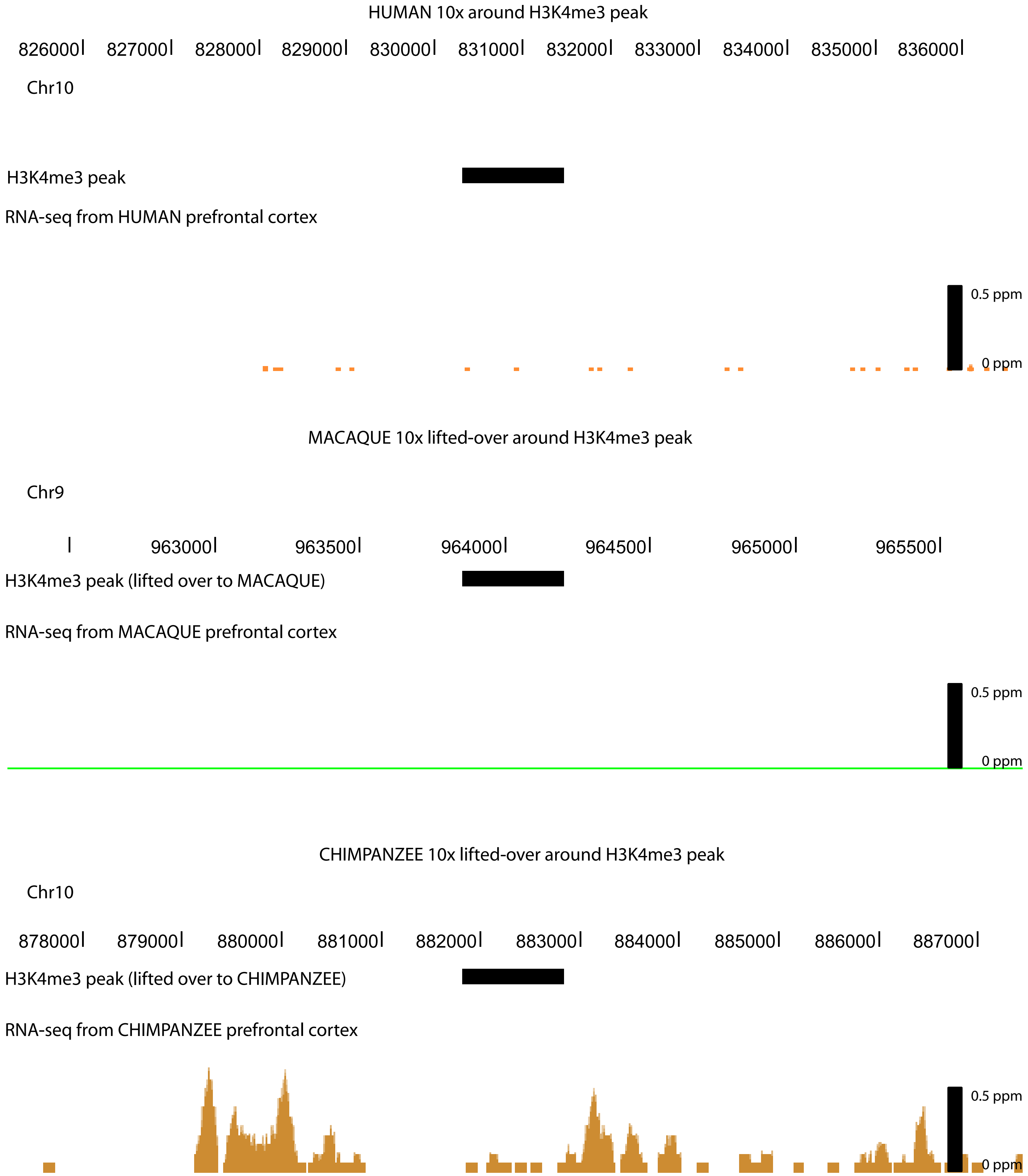
HUMAN 10x around H3K4me3 peak
826000 827000 828000 829000 830000 831000 832000 833000 834000 835000 836000
Chr10
H3K4me3 peak
RNA-seq from HUMAN prefrontal cortex
0.5 ppm
0 ppm
MACAQUE 10x lifted-over around H3K4me3 peak
Chr9
963000 963500 964000 964500 965000 965500
H3K4me3 peak (lifted over to MACAQUE)
RNA-seq from MACAQUE prefrontal cortex
0.5 ppm
0 ppm
CHIMPANZEE 10x lifted-over around H3K4me3 peak
Chr10
878000 879000 880000 881000 882000 883000 884000 885000 886000 887000
H3K4me3 peak (lifted over to CHIMPANZEE)
RNA-seq from CHIMPANZEE prefrontal cortex
0.5 ppm
0 ppm

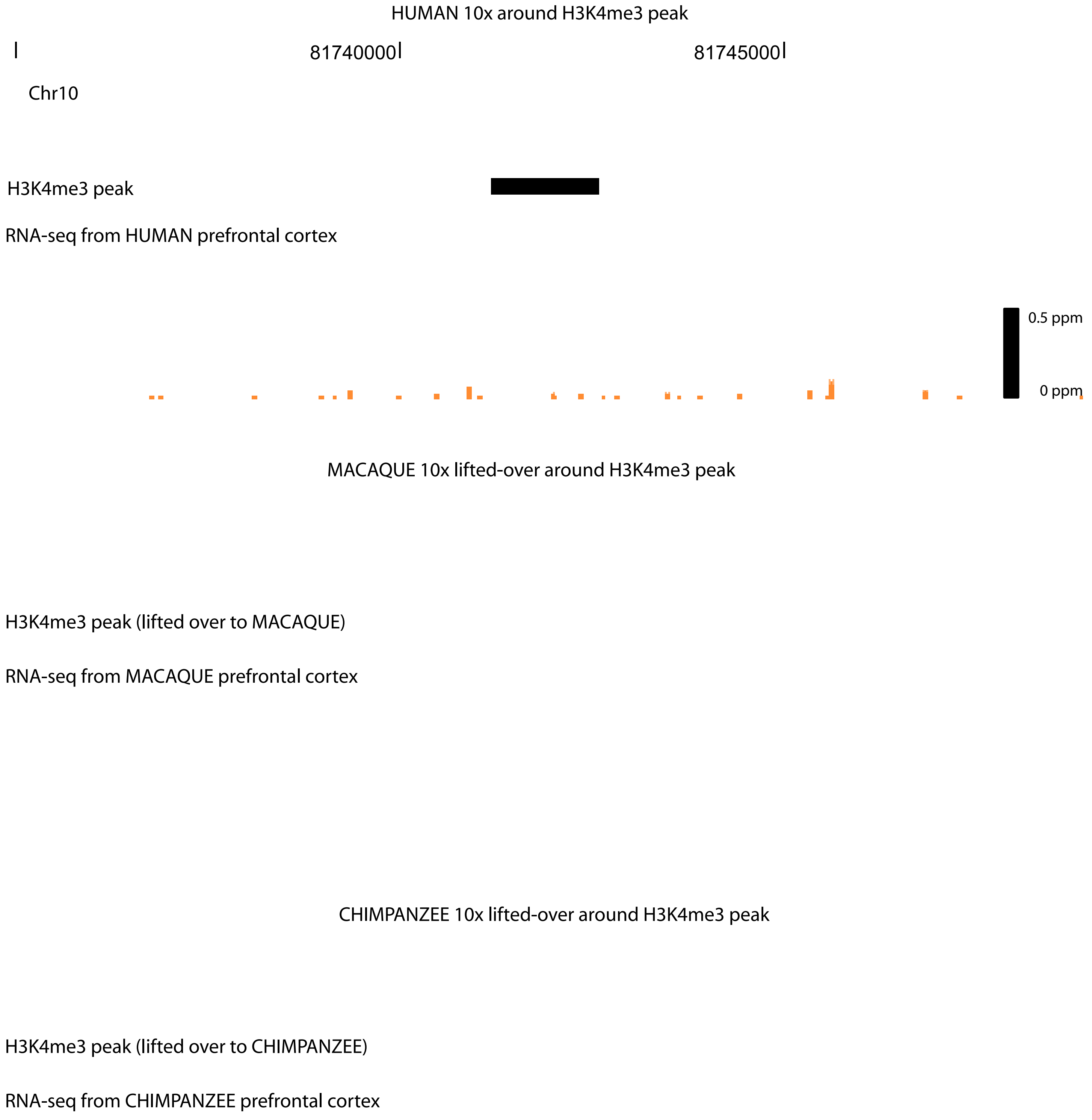
HUMAN 10x around H3K4me3 peak
81740000
81745000
Chr10
H3K4me3 peak
RNA-seq from HUMAN prefrontal cortex
0.5 ppm
0 ppm
MACAQUE 10x lifted-over around H3K4me3 peak
H3K4me3 peak (lifted over to MACAQUE)
RNA-seq from MACAQUE prefrontal cortex
CHIMPANZEE 10x lifted-over around H3K4me3 peak
H3K4me3 peak (lifted over to CHIMPANZEE)
RNA-seq from CHIMPANZEE prefrontal cortex

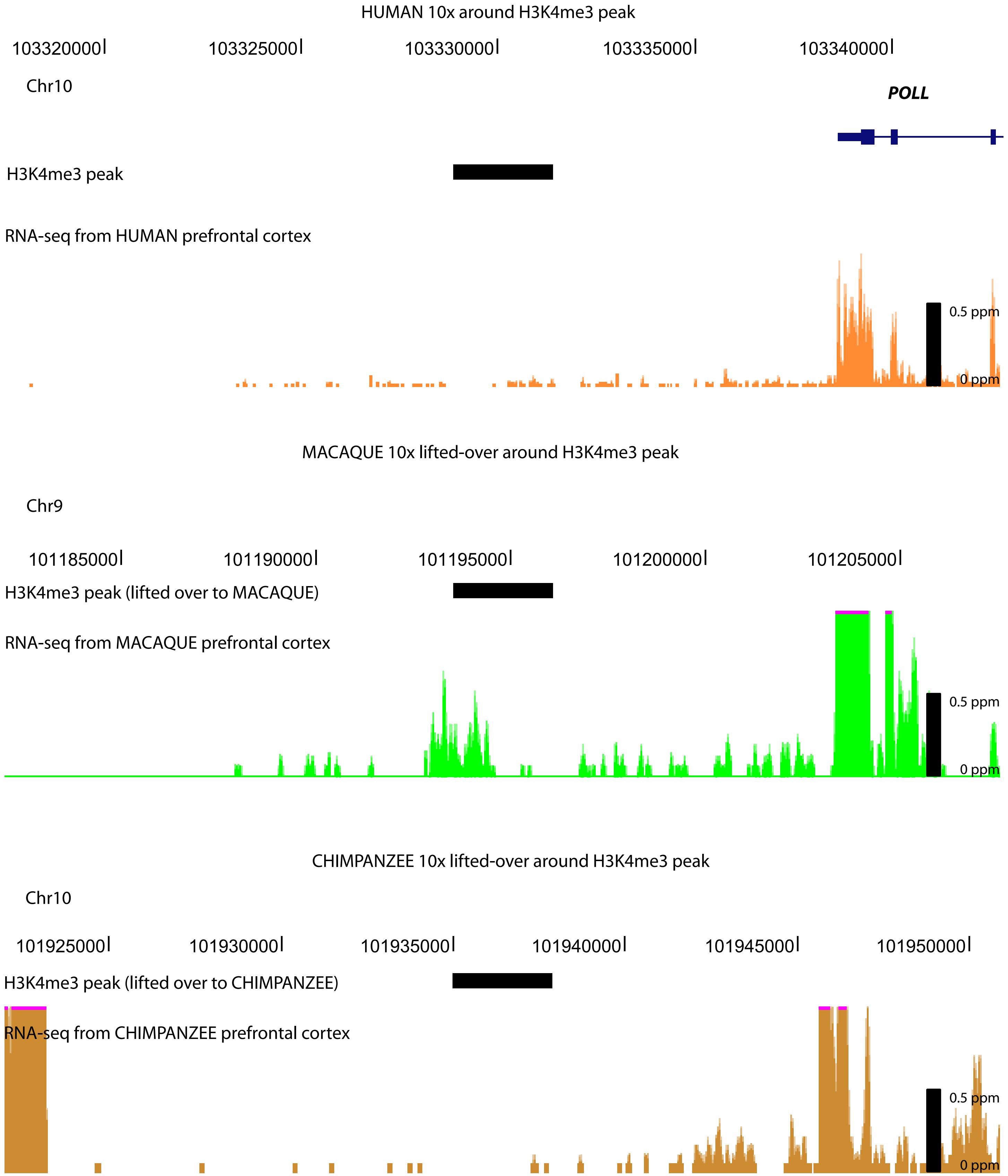
HUMAN 10x around H3K4me3 peak
103320000
103325000
103330000
103335000
103340000
Chr10
POLL
H3K4me3 peak
RNA-seq from HUMAN prefrontal cortex
0.5 ppm
0 ppm
MACAQUE 10x lifted-over around H3K4me3 peak
Chr9
101185000
101190000
101195000
101200000
101205000
H3K4me3 peak (lifted over to MACAQUE)
RNA-seq from MACAQUE prefrontal cortex
0.5 ppm
0 ppm
CHIMPANZEE 10x lifted-over around H3K4me3 peak
Chr10
101925000
101930000
101935000
101940000
101945000
101950000
H3K4me3 peak (lifted over to CHIMPANZEE)
RNA-seq from CHIMPANZEE prefrontal cortex
0.5 ppm
0 ppm

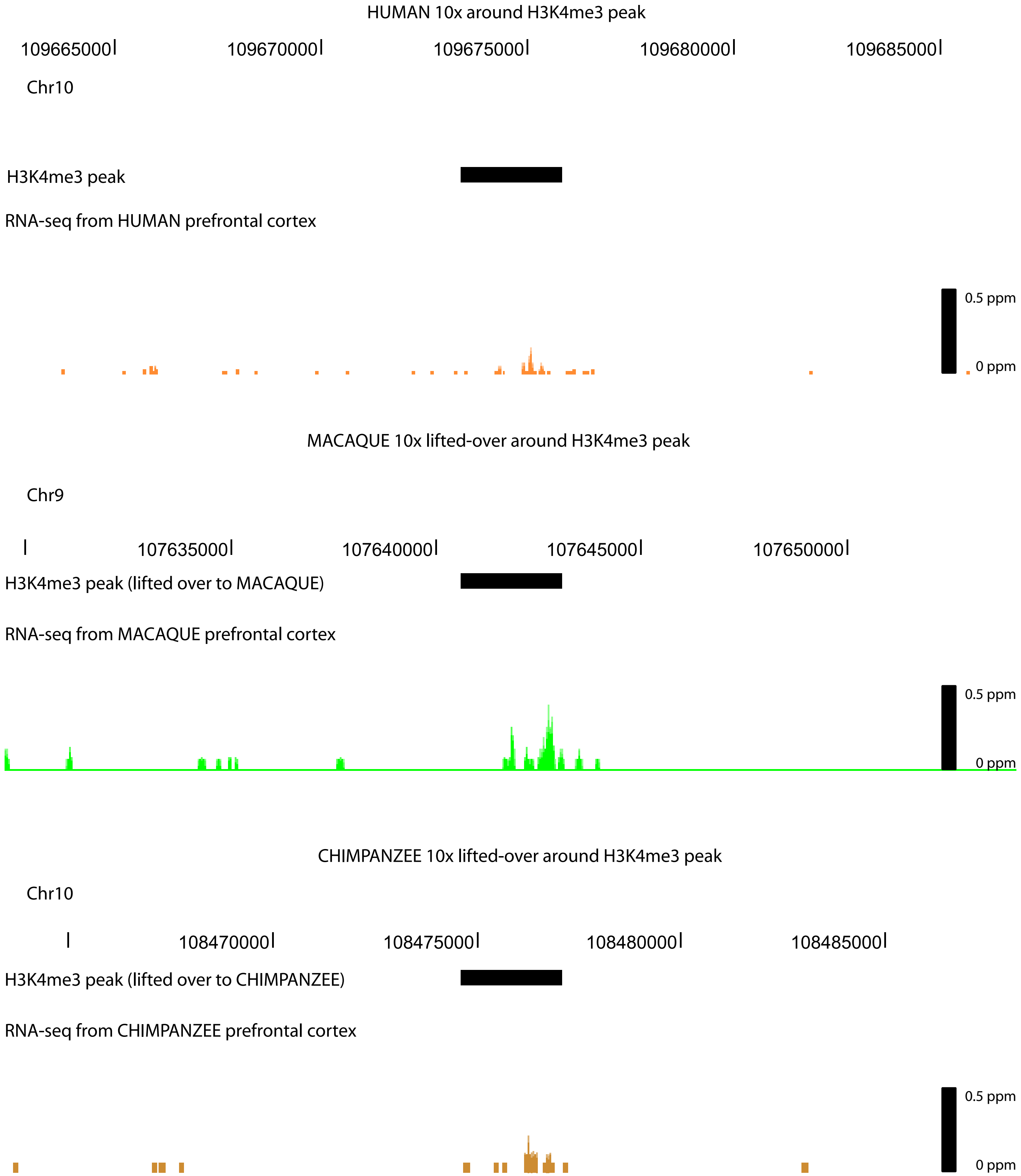

HUMAN 10x around H3K4me3 peak
109665000
109670000
109675000
109680000
109685000
Chr10
H3K4me3 peak
RNA-seq from HUMAN prefrontal cortex
0.5 ppm
0 ppm
MACAQUE 10x lifted-over around H3K4me3 peak
Chr9
107635000
107640000
107645000
107650000
H3K4me3 peak (lifted over to MACAQUE)
RNA-seq from MACAQUE prefrontal cortex
0.5 ppm
0 ppm
CHIMPANZEE 10x lifted-over around H3K4me3 peak
Chr10
108470000
108475000
108480000
108485000
H3K4me3 peak (lifted over to CHIMPANZEE)
RNA-seq from CHIMPANZEE prefrontal cortex
0.5 ppm
0 ppm

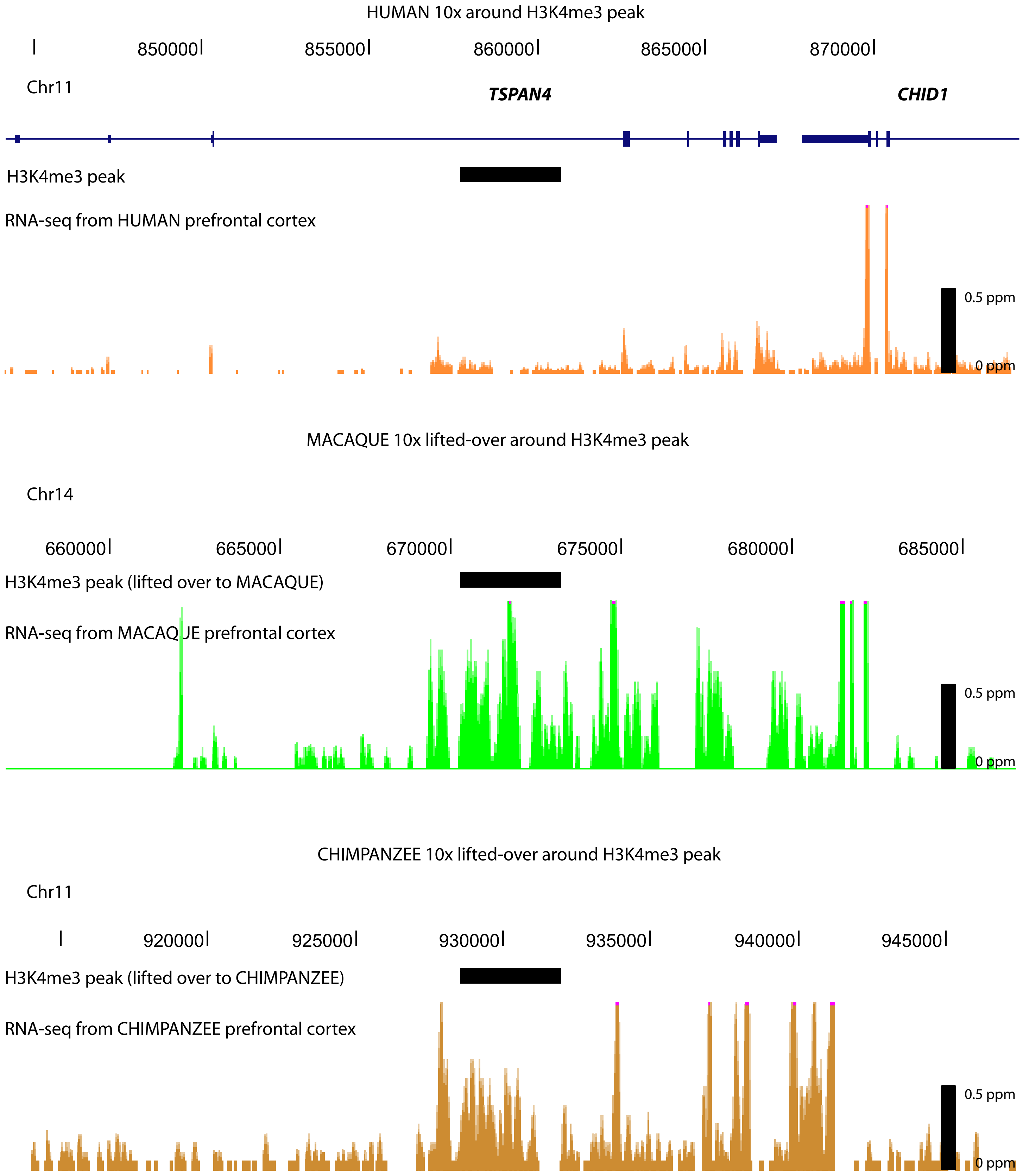

HUMAN 10x around H3K4me3 peak
850000
855000
860000
865000
870000
Chr11
TSPAN4
CHID1
H3K4me3 peak
RNA-seq from HUMAN prefrontal cortex
0.5 ppm
0 ppm
MACAQUE 10x lifted-over around H3K4me3 peak
Chr14
660000
665000
670000
675000
680000
685000
H3K4me3 peak (lifted over to MACAQUE)
RNA-seq from MACAQUE prefrontal cortex
0.5 ppm
0 ppm
CHIMPANZEE 10x lifted-over around H3K4me3 peak
Chr11
920000
925000
930000
935000
940000
945000
H3K4me3 peak (lifted over to CHIMPANZEE)
RNA-seq from CHIMPANZEE prefrontal cortex
0.5 ppm
0 ppm

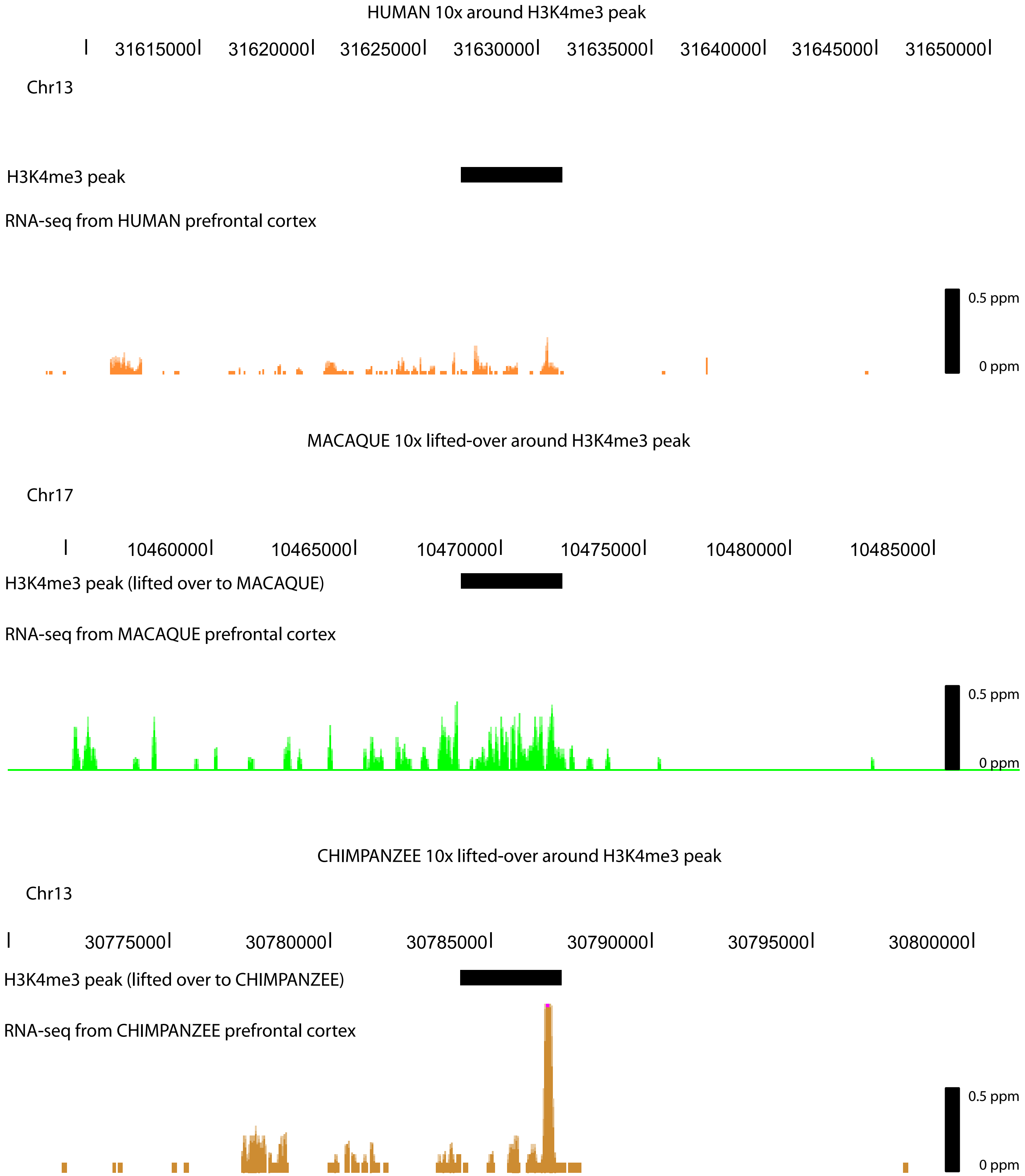
HUMAN 10x around H3K4me3 peak
31615000 31620000 31625000 31630000 31635000 31640000 31645000 31650000
Chr13
H3K4me3 peak
RNA-seq from HUMAN prefrontal cortex
0.5 ppm
0 ppm
MACAQUE 10x lifted-over around H3K4me3 peak
Chr17
10460000 10465000 10470000 10475000 10480000 10485000
H3K4me3 peak (lifted over to MACAQUE)
RNA-seq from MACAQUE prefrontal cortex
0.5 ppm
0 ppm
CHIMPANZEE 10x lifted-over around H3K4me3 peak
Chr13
30775000 30780000 30785000 30790000 30795000 30800000
H3K4me3 peak (lifted over to CHIMPANZEE)
RNA-seq from CHIMPANZEE prefrontal cortex
0.5 ppm
0 ppm

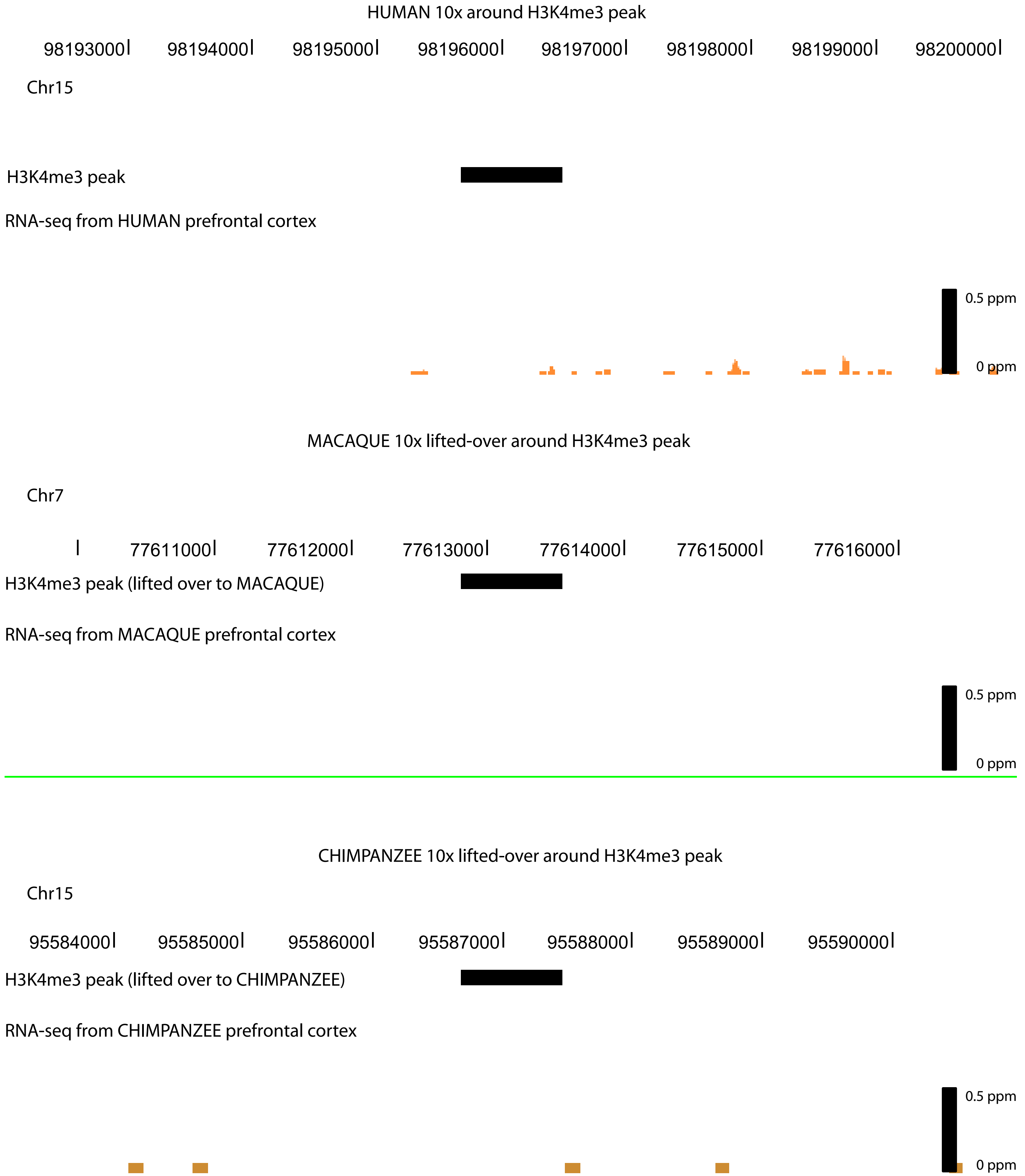
HUMAN 10x around H3K4me3 peak
98193000
98194000
98195000
98196000
98197000
98198000
98199000
98200000
Chr15
H3K4me3 peak
RNA-seq from HUMAN prefrontal cortex
0.5 ppm
0 ppm
MACAQUE 10x lifted-over around H3K4me3 peak
Chr7
77611000
77612000
77613000
77614000
77615000
77616000
H3K4me3 peak (lifted over to MACAQUE)
RNA-seq from MACAQUE prefrontal cortex
0.5 ppm
0 ppm
CHIMPANZEE 10x lifted-over around H3K4me3 peak
Chr15
95584000
95585000
95586000
95587000
95588000
95589000
95590000
H3K4me3 peak (lifted over to CHIMPANZEE)
RNA-seq from CHIMPANZEE prefrontal cortex
0.5 ppm
0 ppm

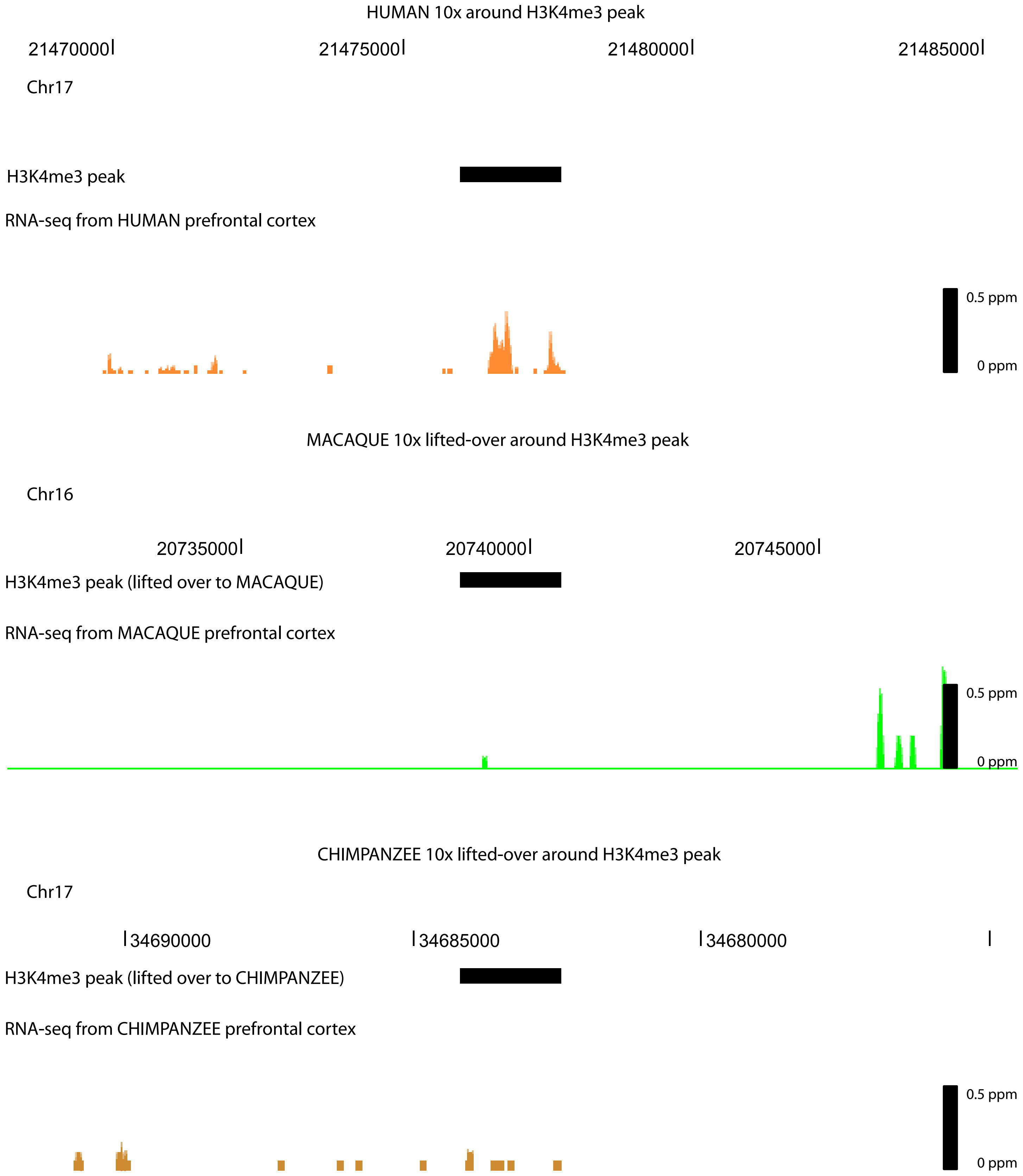
HUMAN 10x around H3K4me3 peak
21470000
21475000
21480000
21485000
Chr17
H3K4me3 peak
RNA-seq from HUMAN prefrontal cortex
0.5 ppm
0 ppm
MACAQUE 10x lifted-over around H3K4me3 peak
Chr16
20735000
20740000
20745000
H3K4me3 peak (lifted over to MACAQUE)
RNA-seq from MACAQUE prefrontal cortex
0.5 ppm
0 ppm
CHIMPANZEE 10x lifted-over around H3K4me3 peak
Chr17
34690000
34685000
34680000
H3K4me3 peak (lifted over to CHIMPANZEE)
RNA-seq from CHIMPANZEE prefrontal cortex
0.5 ppm
0 ppm

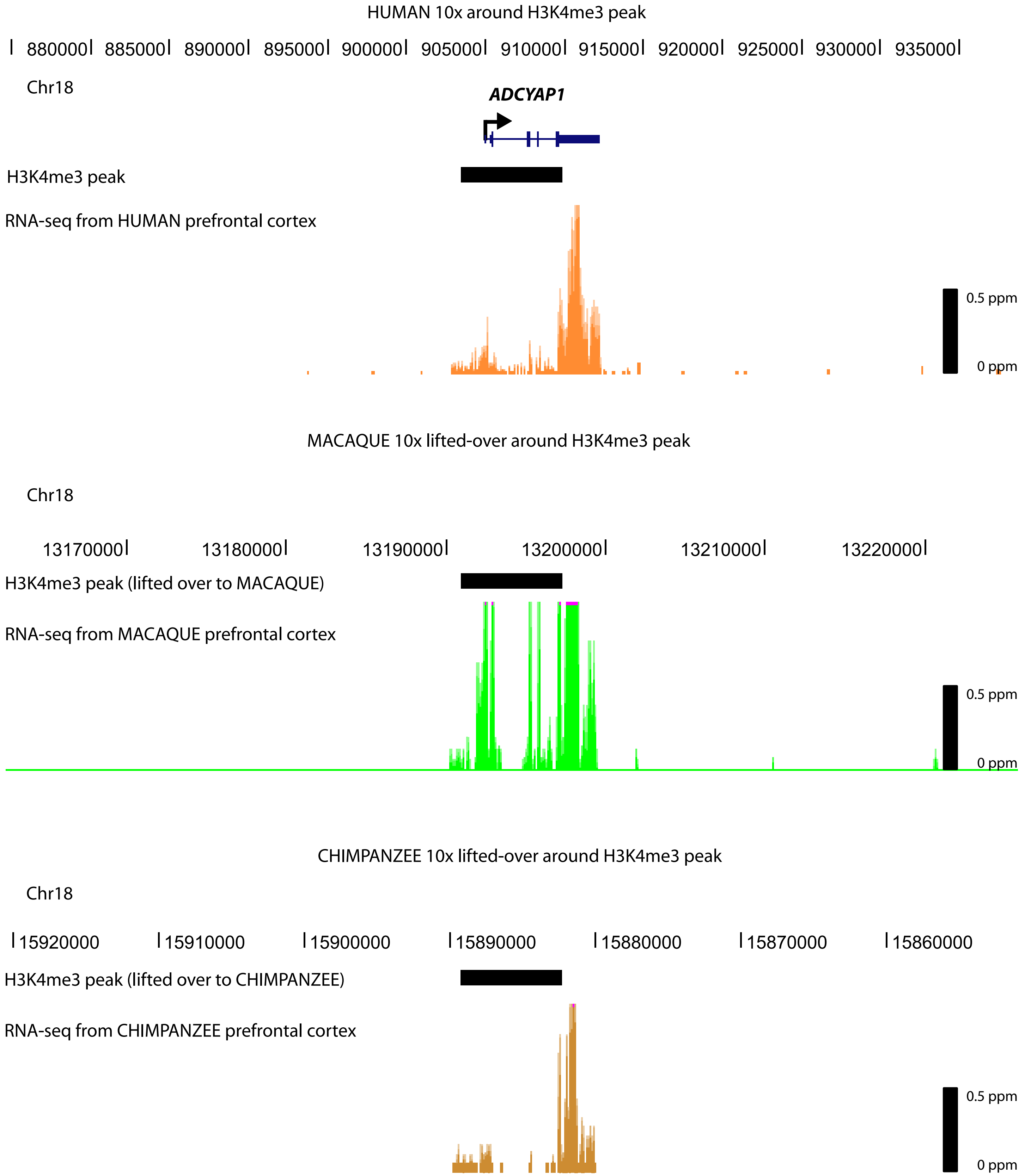
HUMAN 10x around H3K4me3 peak
880000 885000 890000 895000 900000 905000 910000 915000 920000 925000 930000 935000
Chr18
ADCYAP1
H3K4me3 peak
RNA-seq from HUMAN prefrontal cortex
0.5 ppm
0 ppm
MACAQUE 10x lifted-over around H3K4me3 peak
Chr18
13170000 13180000 13190000 13200000 13210000 13220000
H3K4me3 peak (lifted over to MACAQUE)
RNA-seq from MACAQUE prefrontal cortex
0.5 ppm
0 ppm
CHIMPANZEE 10x lifted-over around H3K4me3 peak
Chr18
15920000 15910000 15900000 15890000 15880000 15870000 15860000
H3K4me3 peak (lifted over to CHIMPANZEE)
RNA-seq from CHIMPANZEE prefrontal cortex
0.5 ppm
0 ppm

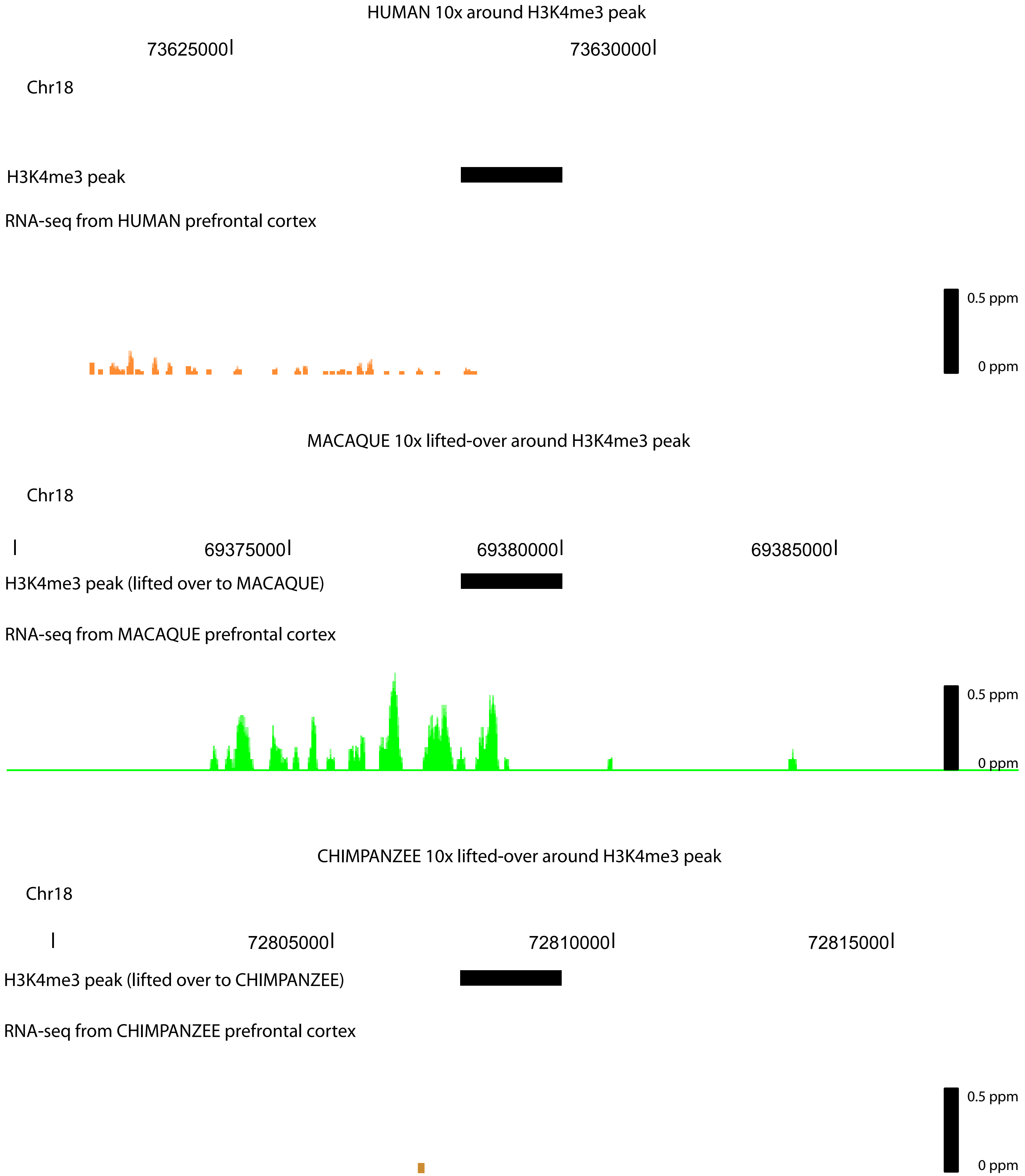
HUMAN 10x around H3K4me3 peak
73625000
73630000
Chr18
H3K4me3 peak
RNA-seq from HUMAN prefrontal cortex
0.5 ppm
0 ppm
MACAQUE 10x lifted-over around H3K4me3 peak
Chr18
69375000
69380000
69385000
H3K4me3 peak (lifted over to MACAQUE)
RNA-seq from MACAQUE prefrontal cortex
0.5 ppm
0 ppm
CHIMPANZEE 10x lifted-over around H3K4me3 peak
Chr18
72805000
72810000
72815000
H3K4me3 peak (lifted over to CHIMPANZEE)
RNA-seq from CHIMPANZEE prefrontal cortex
0.5 ppm
0 ppm

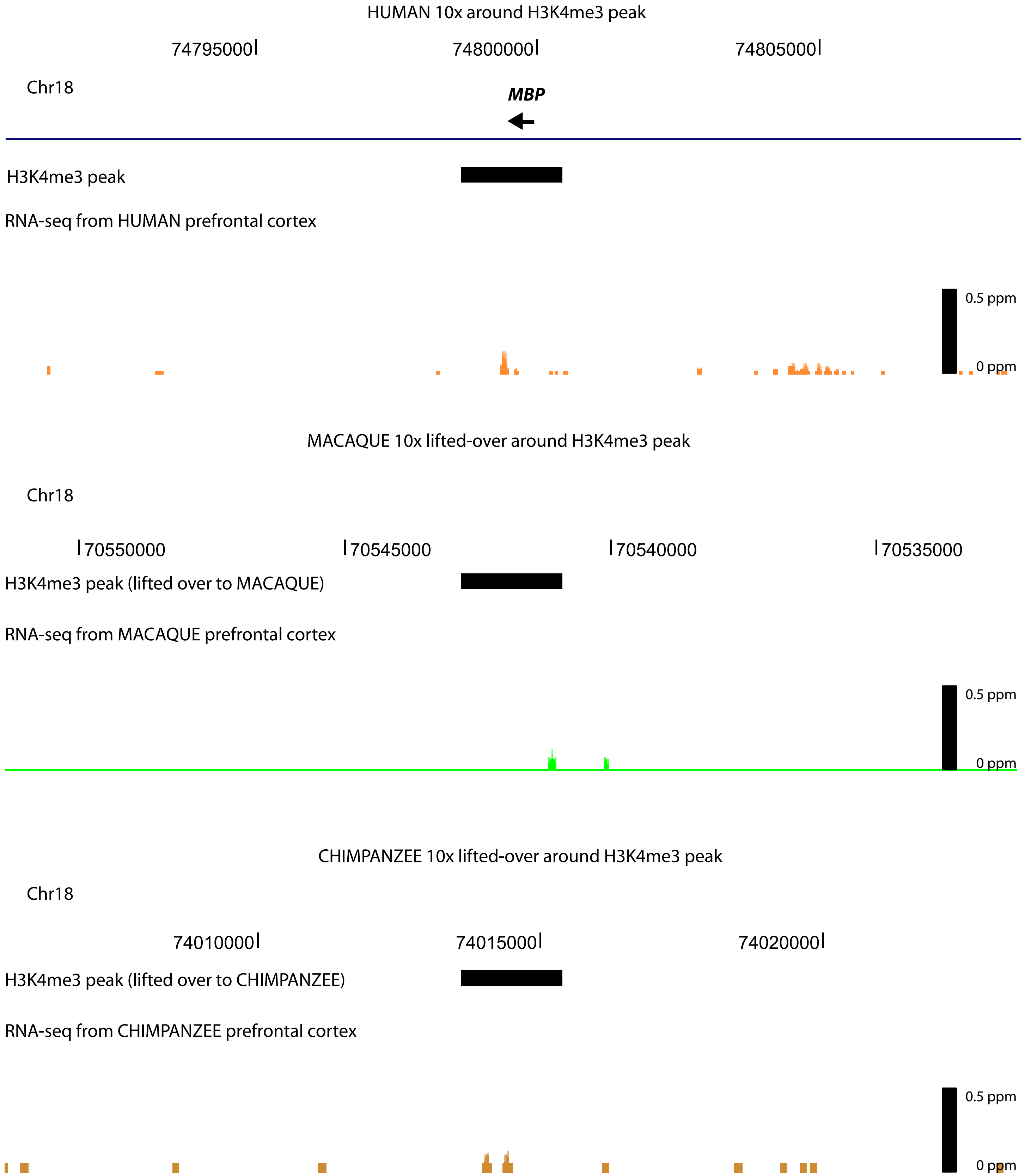

HUMAN 10x around H3K4me3 peak
74795000
74800000
74805000
Chr18
MBP
H3K4me3 peak
RNA-seq from HUMAN prefrontal cortex
0.5 ppm
0 ppm
MACAQUE 10x lifted-over around H3K4me3 peak
Chr18
70550000
70545000
70540000
70535000
H3K4me3 peak (lifted over to MACAQUE)
RNA-seq from MACAQUE prefrontal cortex
0.5 ppm
0 ppm
CHIMPANZEE 10x lifted-over around H3K4me3 peak
Chr18
74010000
74015000
74020000
H3K4me3 peak (lifted over to CHIMPANZEE)
RNA-seq from CHIMPANZEE prefrontal cortex
0.5 ppm
0 ppm

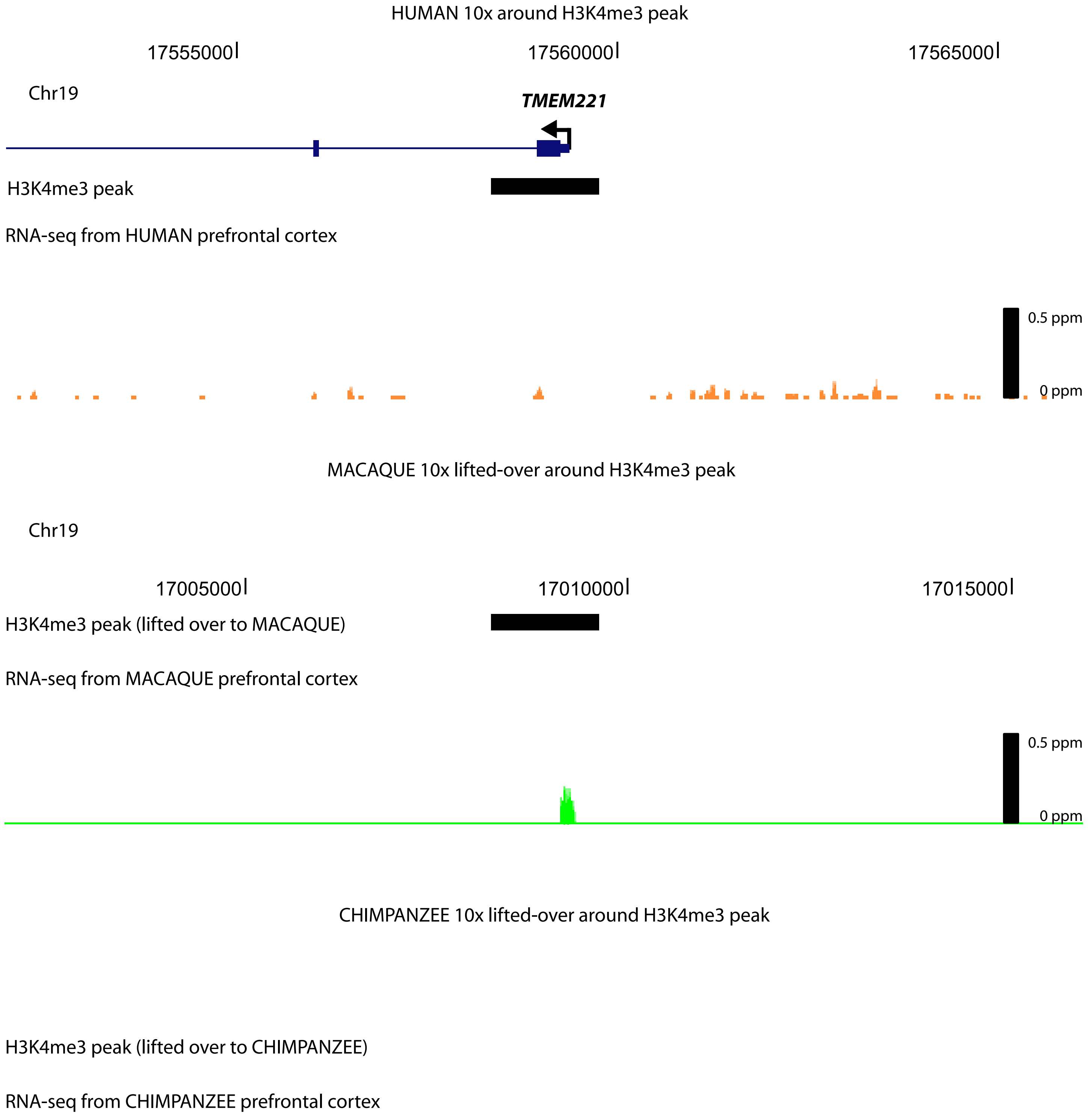

HUMAN 10x around H3K4me3 peak
17555000
17560000
17565000
Chr19
TMEM221
H3K4me3 peak
RNA-seq from HUMAN prefrontal cortex
0.5 ppm
0 ppm
MACAQUE 10x lifted-over around H3K4me3 peak
Chr19
17005000
17010000
17015000
H3K4me3 peak (lifted over to MACAQUE)
RNA-seq from MACAQUE prefrontal cortex
0.5 ppm
0 ppm
CHIMPANZEE 10x lifted-over around H3K4me3 peak
H3K4me3 peak (lifted over to CHIMPANZEE)
RNA-seq from CHIMPANZEE prefrontal cortex

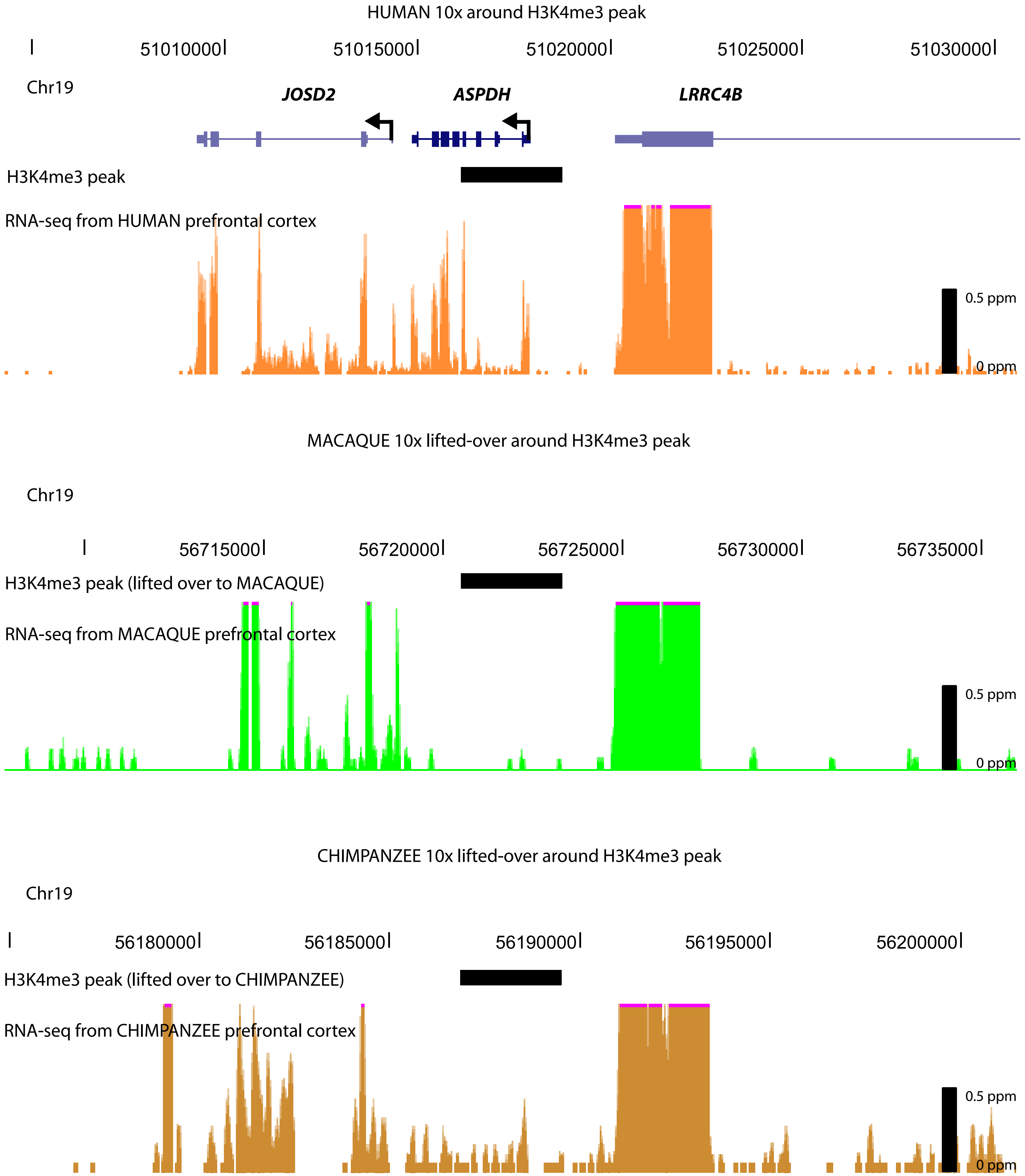
HUMAN 10x around H3K4me3 peak
51010000
51015000
51020000
51025000
51030000
Chr19
JOSD2
ASPDH
LRRC4B
H3K4me3 peak
RNA-seq from HUMAN prefrontal cortex
0.5 ppm
0 ppm
MACAQUE 10x lifted-over around H3K4me3 peak
Chr19
56715000
56720000
56725000
56730000
56735000
H3K4me3 peak (lifted over to MACAQUE)
RNA-seq from MACAQUE prefrontal cortex
0.5 ppm
0 ppm
CHIMPANZEE 10x lifted-over around H3K4me3 peak
Chr19
56180000
56185000
56190000
56195000
56200000
H3K4me3 peak (lifted over to CHIMPANZEE)
RNA-seq from CHIMPANZEE prefrontal cortex
0.5 ppm
0 ppm

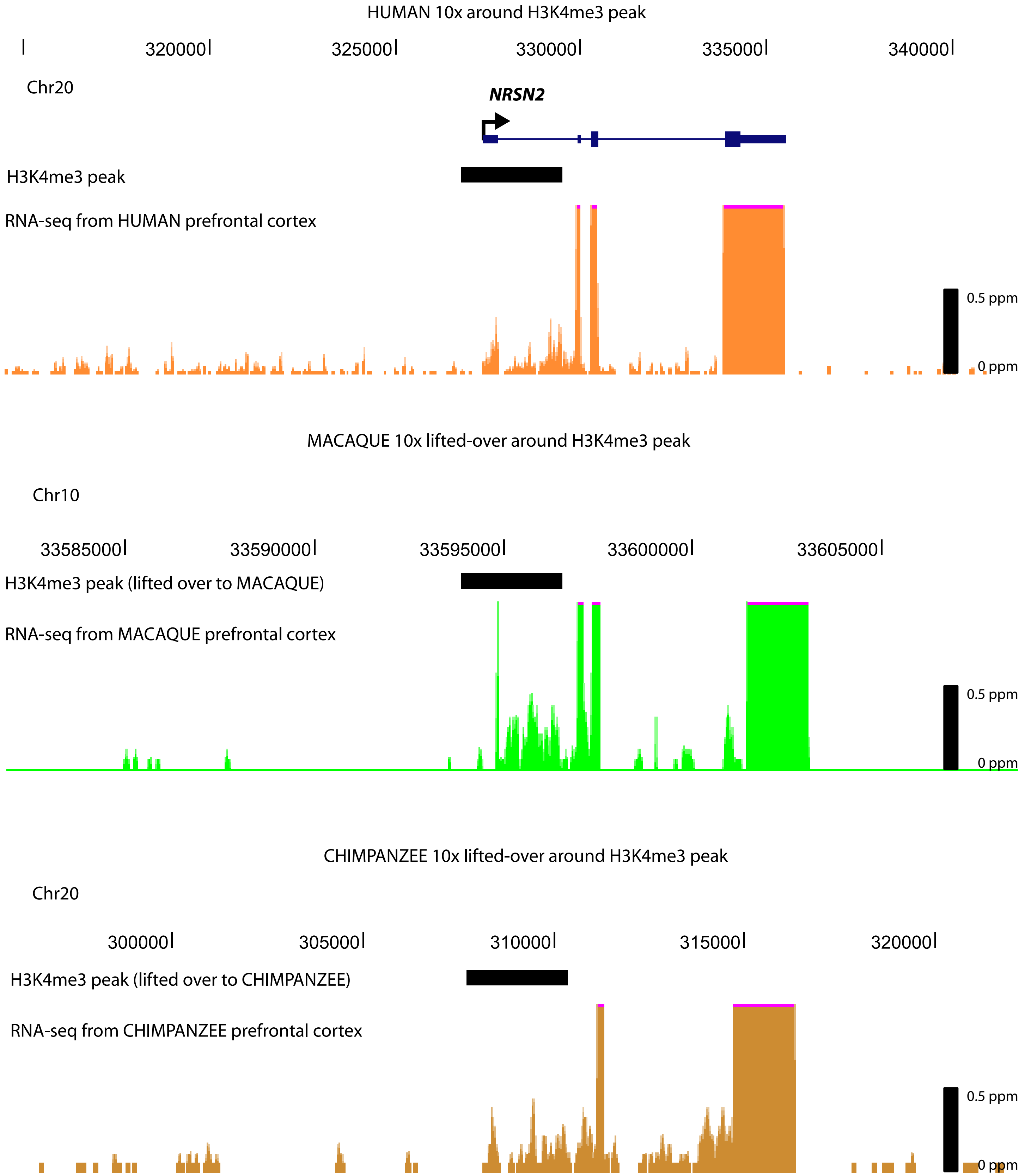
HUMAN 10x around H3K4me3 peak
320000
325000
330000
335000
340000
Chr20
NRSN2
H3K4me3 peak
RNA-seq from HUMAN prefrontal cortex
0.5 ppm
0 ppm
MACAQUE 10x lifted-over around H3K4me3 peak
Chr10
33585000
33590000
33595000
33600000
33605000
H3K4me3 peak (lifted over to MACAQUE)
RNA-seq from MACAQUE prefrontal cortex
0.5 ppm
0 ppm
CHIMPANZEE 10x lifted-over around H3K4me3 peak
Chr20
300000
305000
310000
315000
320000
H3K4me3 peak (lifted over to CHIMPANZEE)
RNA-seq from CHIMPANZEE prefrontal cortex
0.5 ppm
0 ppm

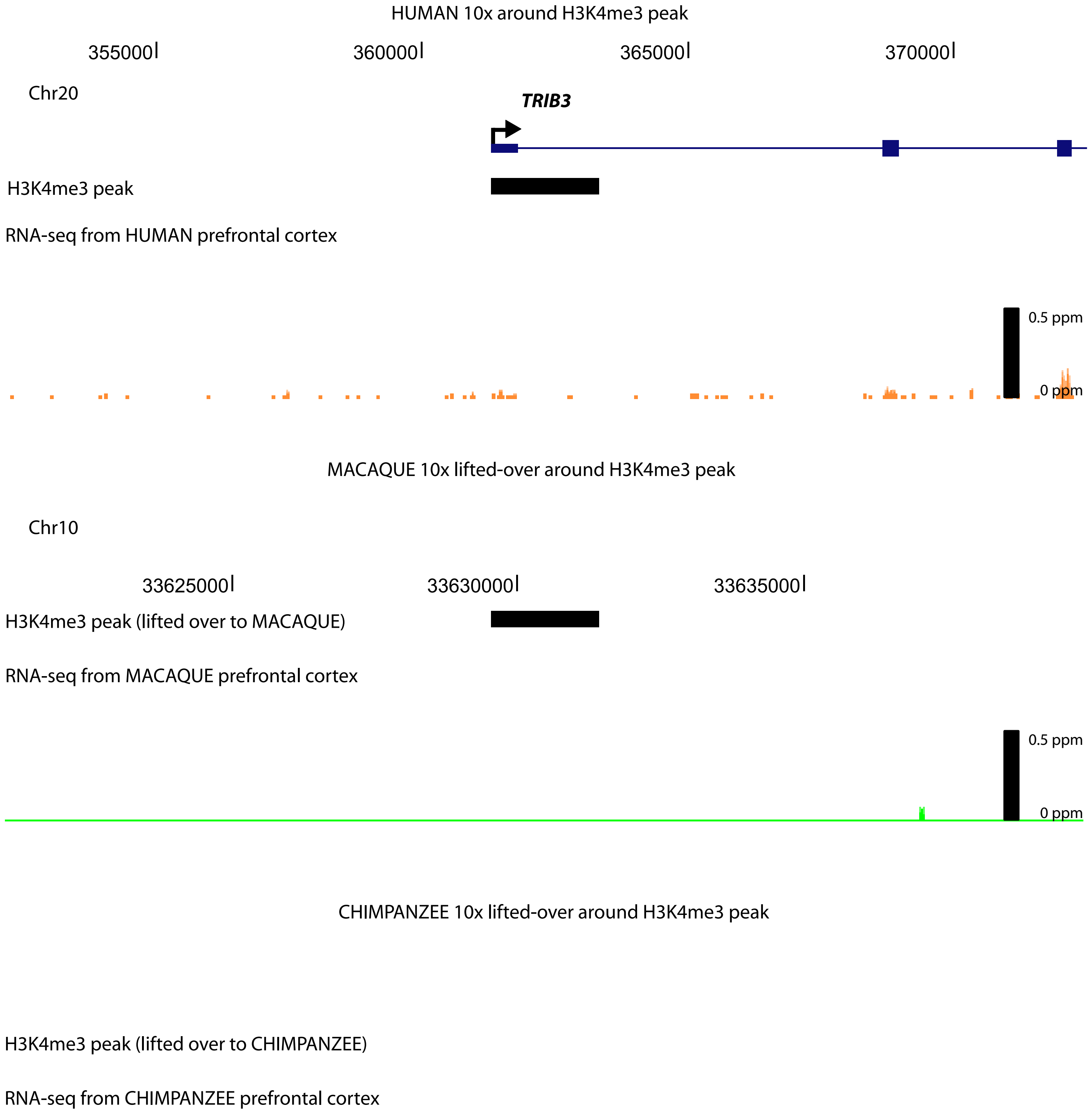

HUMAN 10x around H3K4me3 peak
355000
360000
365000
370000
Chr20
TRIB3
H3K4me3 peak
RNA-seq from HUMAN prefrontal cortex
0.5 ppm
0 ppm
MACAQUE 10x lifted-over around H3K4me3 peak
Chr10
33625000
33630000
33635000
H3K4me3 peak (lifted over to MACAQUE)
RNA-seq from MACAQUE prefrontal cortex
0.5 ppm
0 ppm
CHIMPANZEE 10x lifted-over around H3K4me3 peak
H3K4me3 peak (lifted over to CHIMPANZEE)
RNA-seq from CHIMPANZEE prefrontal cortex

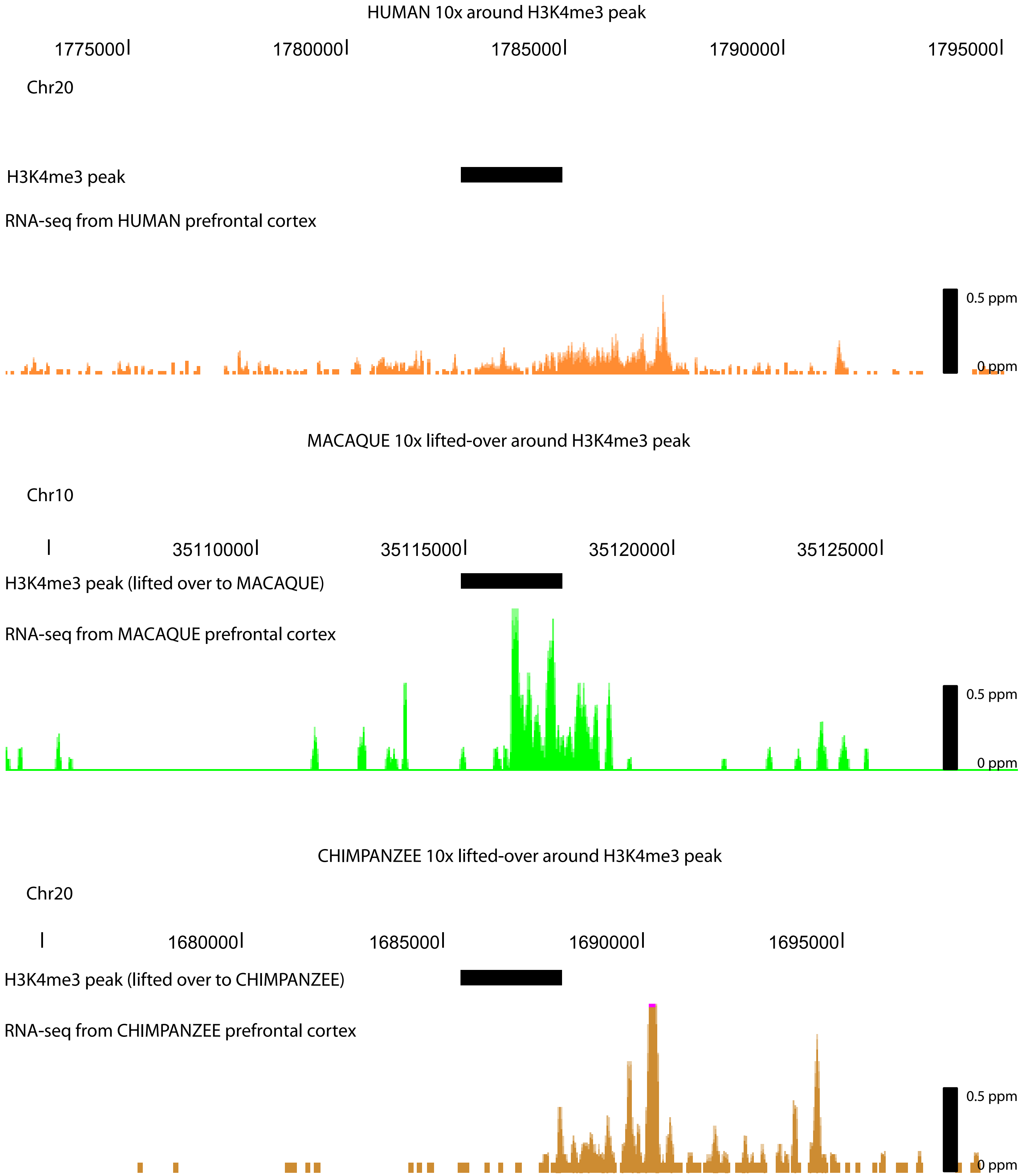
HUMAN 10x around H3K4me3 peak
1775000
1780000
1785000
1790000
1795000
Chr20
H3K4me3 peak
RNA-seq from HUMAN prefrontal cortex
0.5 ppm
0 ppm
MACAQUE 10x lifted-over around H3K4me3 peak
Chr10
35110000
35115000
35120000
35125000
H3K4me3 peak (lifted over to MACAQUE)
RNA-seq from MACAQUE prefrontal cortex
0.5 ppm
0 ppm
CHIMPANZEE 10x lifted-over around H3K4me3 peak
Chr20
1680000
1685000
1690000
1695000
H3K4me3 peak (lifted over to CHIMPANZEE)
RNA-seq from CHIMPANZEE prefrontal cortex
0.5 ppm
0 ppm

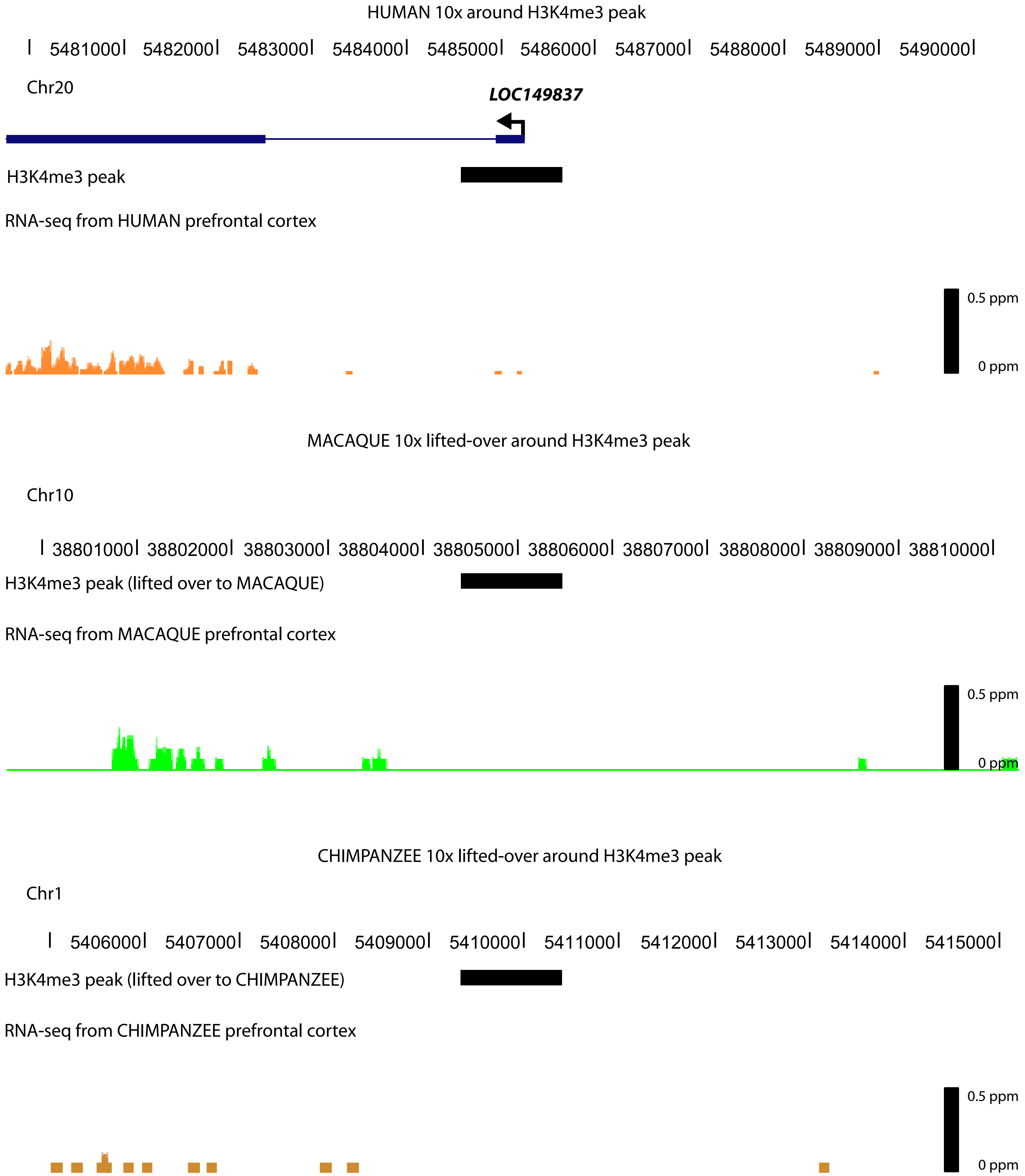
HUMAN 10x around H3K4me3 peak
5481000 5482000 5483000 5484000 5485000 5486000 5487000 5488000 5489000 5490000
Chr20
LOC149837
H3K4me3 peak
RNA-seq from HUMAN prefrontal cortex
0.5 ppm
0 ppm
MACAQUE 10x lifted-over around H3K4me3 peak
Chr10
38801000 38802000 38803000 38804000 38805000 38806000 38807000 38808000 38809000 38810000
H3K4me3 peak (lifted over to MACAQUE)
RNA-seq from MACAQUE prefrontal cortex
0.5 ppm
0 ppm
CHIMPANZEE 10x lifted-over around H3K4me3 peak
Chr1
5406000 5407000 5408000 5409000 5410000 5411000 5412000 5413000 5414000 5415000
H3K4me3 peak (lifted over to CHIMPANZEE)
RNA-seq from CHIMPANZEE prefrontal cortex
0.5 ppm
0 ppm

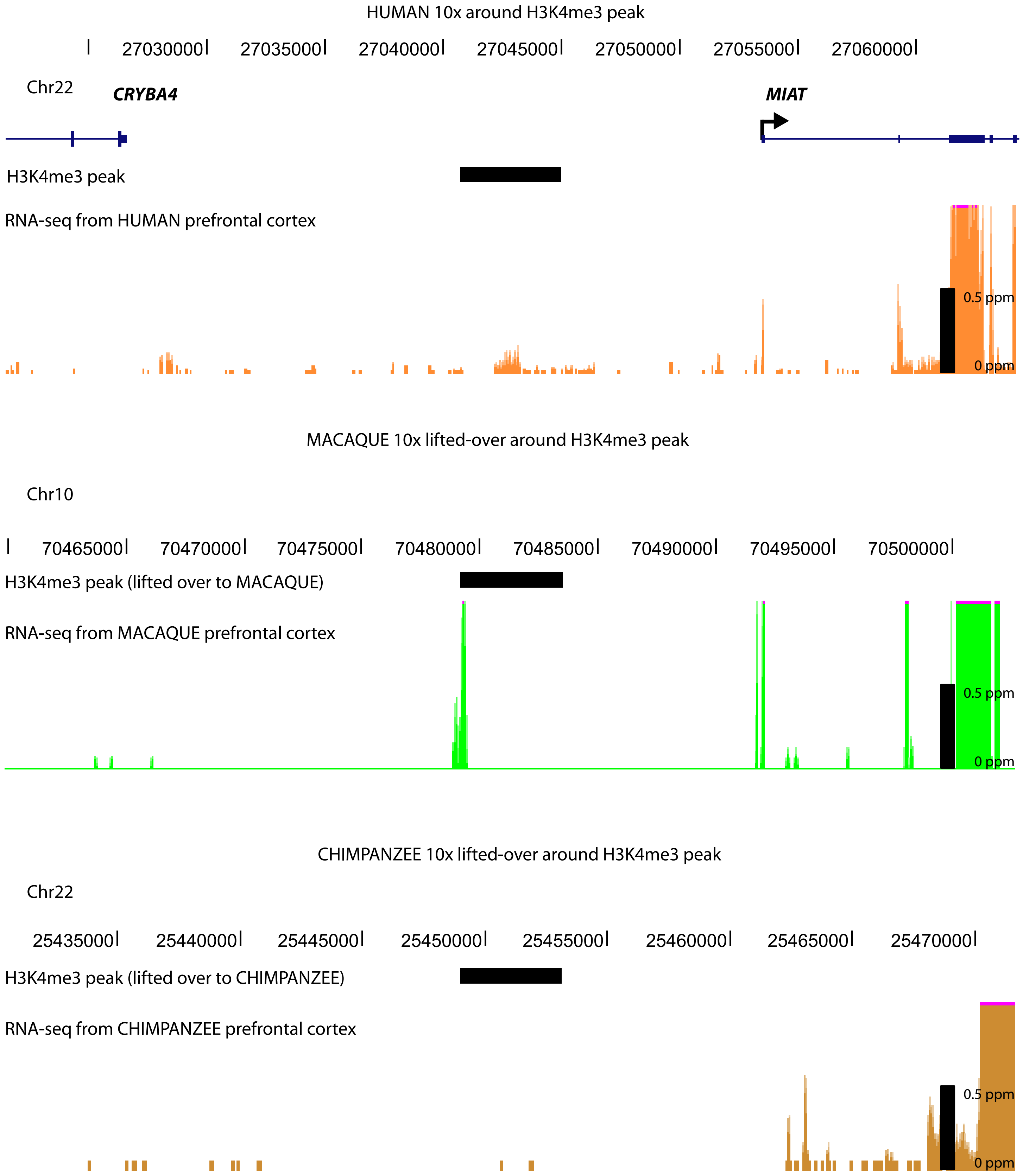
HUMAN 10x around H3K4me3 peak
27030000
27035000
27040000
27045000
27050000
27055000
27060000
Chr22
CRYBA4
MIAT
H3K4me3 peak
RNA-seq from HUMAN prefrontal cortex
0.5 ppm
0 ppm
MACAQUE 10x lifted-over around H3K4me3 peak
Chr10
70465000
70470000
70475000
70480000
70485000
70490000
70495000
70500000
H3K4me3 peak (lifted over to MACAQUE)
RNA-seq from MACAQUE prefrontal cortex
0.5 ppm
0 ppm
CHIMPANZEE 10x lifted-over around H3K4me3 peak
Chr22
25435000
25440000
25445000
25450000
25455000
25460000
25465000
25470000
H3K4me3 peak (lifted over to CHIMPANZEE)
RNA-seq from CHIMPANZEE prefrontal cortex
0.5 ppm
0 ppm

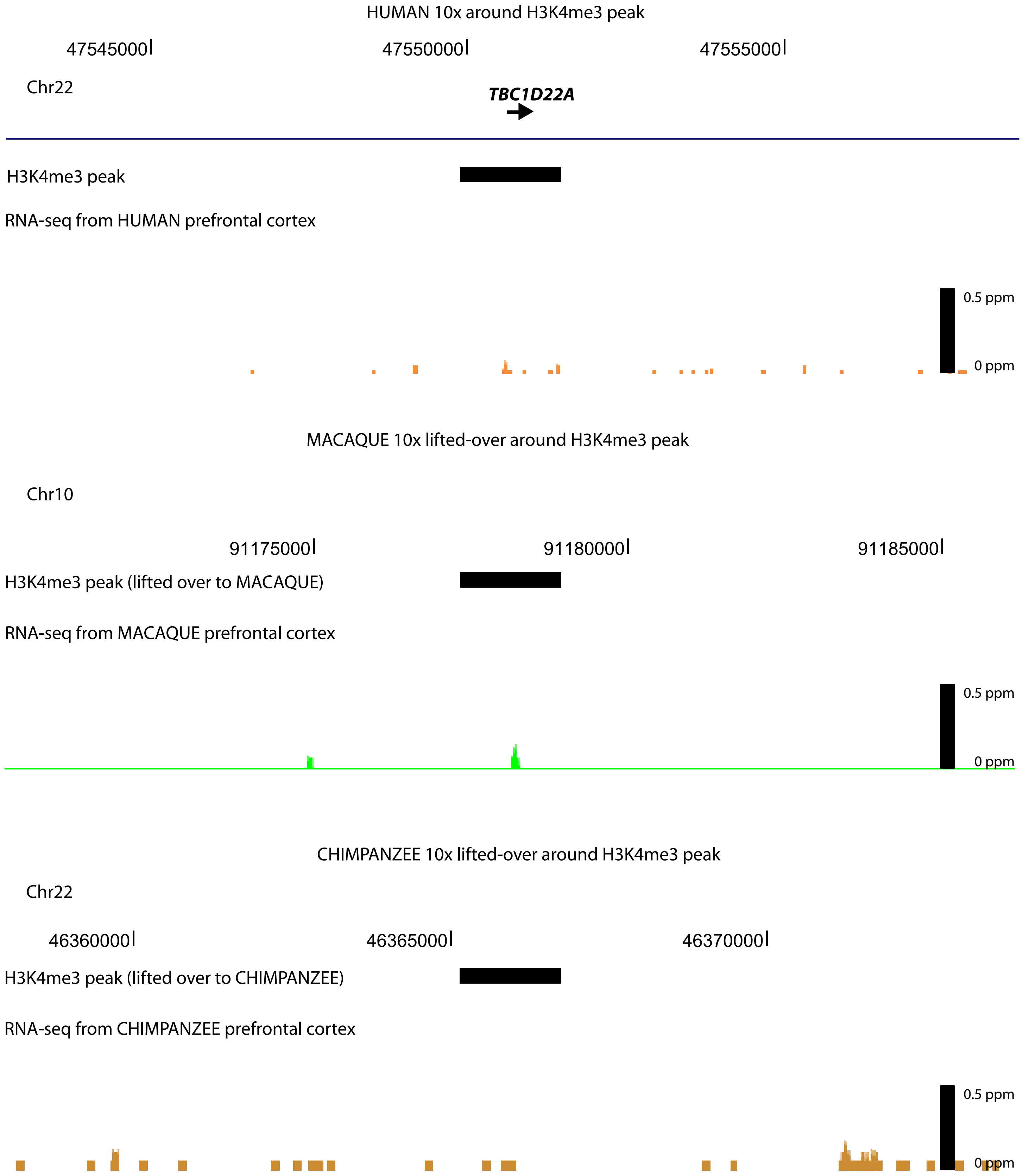
HUMAN 10x around H3K4me3 peak
47545000
47550000
47555000
Chr22
TBC1D22A
H3K4me3 peak
RNA-seq from HUMAN prefrontal cortex
0.5 ppm
0 ppm
MACAQUE 10x lifted-over around H3K4me3 peak
Chr10
91175000
91180000
91185000
H3K4me3 peak (lifted over to MACAQUE)
RNA-seq from MACAQUE prefrontal cortex
0.5 ppm
0 ppm
CHIMPANZEE 10x lifted-over around H3K4me3 peak
Chr22
46360000
46365000
46370000
H3K4me3 peak (lifted over to CHIMPANZEE)
RNA-seq from CHIMPANZEE prefrontal cortex
0.5 ppm
0 ppm
