# Supplementary material for: Human-Specific Histone Methylation Signatures at Transcription Start Sites in Prefrontal Neurons
Source: PLoS Biol. 2012 Nov 20;10(11):e1001427. doi: 10.1371/journal.pbio.1001427 (PMC3502543; doi:10.1371/journal.pbio.1001427)
Supplement: Text S1 — Methods. (DOC) [file pbio.1001427.s022.doc]

**Supplemental Methods (Shulha, Crisci, Reshetov, Tushir et al.)**

**Sample preparation (ChIP-seq and RNA-seq)**

Ethics Statement: All work presented here was conducted on brain specimens collected after death. Cause of death was unrelated to the present study.. All sample acquisition and processing of postmortem brain tissue was approved by the Institutional Review Boards of the participating institutions.

ChIP-seq: Procedures for extraction and sorting of NeuN+ neuronal nuclei from the cortical gray matter, and subsequent chromatin immunoprecipitation with anti-H3K4me3 antibody and ChIP-seq library preparation were recently described . Cross-immunoreactivity of the anti-H3K4me3 antibody with other histone methylation forms, including mono- and di-H3K4 (H3K4me1/2) was controlled by dot blots and synthetic blocking peptides as described . The human ChIP-seq data sets, generated from neuronal nuclei of the pole of the frontal lobe, were published previously.

Postmortem brain tissue from the pole of the frontal lobe of 4 adult chimpanzees, ranging in age from 27-44 years, and of 3 adult macaque monkeys 11 years or older (**Table S1**) was processed in the same manner as previously described for the human specimens. Specimens were obtained from the dorsolateral portion of the prefrontal cortex, primarily from cytoarchitectonic (Brodmann) Area 10 (BA10) and regions that border on BA10, including portions of BA9 and BA46. The quality of ChIP-seq datasets was similar across all samples, with the total number of reads in the range of 2-10^6^, of which typically 70-80% were derived from uniquely mappable sequences of the reference genome (HG19 or panTro2 or rheMac2) (**Table S1**).

RNA-seq: Three human specimens with no evidence for neurological disease or neurodegeneration were obtained from the Harvard Brain Tissue Resource Center in Belmont, MA (age 69-70, postmortem interval 15-26 hrs, all male). Rostral prefrontal cortex was processed for RNA-seq using Illumina’s *mRNA-seq sample preparation kit*. Briefly, total RNAs were isolated using Trizol isolation kit and ploy-A containing RNAs were purified using poly-T oligo-attached magnetic beads. The mRNA was then fragmented into small pieces using divalent cations under elevated temperature and the cleaved RNA fragments were copied into first strand cDNA using reverse transcriptase and random primers. RNA integrity number for each sample was determined using the Agilent 2100 bioanalyzer, RIN was above 4.0 or all cases. Second strand cDNA was synthesized using DNA polymerase I and RNAseH and followed by poly “A” cloning and PCR amplification to create the final cDNA library. RNA-Seq data were generated on an Illumina Genome Analyzer IIx by single end sequencing with 35 nucleotide (nt) read length.


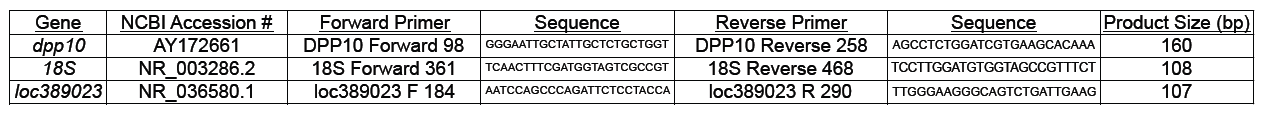
To further confirm species-specific differences, RNA from the frontal pole was isolated using the RNEasy Mini Kit (Qiagen, Valencia, CA). RNA concentrations were equilibrated to 100 ng/uL, and qRT-PCR was performed using the Quantifast SYBR Green RT-PCR kit on an AB7500 machine (Qiagen, Applied Biosystems, Carlsbad, CA) using primers shown here. Relative expression was determined using the Pfaffl method normalized to 18S and referenced to human expression. All products were sequenced for verification of specificity.

**ChIP-seq analysis**

Libraries were sequenced with the Illumina Genome Analyzer GAII, and images were first processed with GAPipeline (versions 1.0 and 1.4) and OLB (1.6). We performed single-end sequencing of 36bp reads. We used Bowtie (version 0.11.3) allowing up to one mismatch to map all sequence reads to the gender appropriate human genome HG19 and only retained the reads that mapped to one unique location in the genome in each sample for subsequent data analysis. Chimpanzee and macaque datasets were mapped to the appropriate genomes (rheMac2 and panTro2) and to human genome HG19 for comparison. **Table S1** online shows the sequencing statistics.

To calculate the table of Pearson correlations among H3K4me3 profiles in promoters (**Fig.1A, Table S2**), the region within 2 kb of a transcriptional start site (TSS) was defined as the promoter of the TSS. If a gene has multiple TSSs, each TSS was accounted for separately. We used the RefSeq gene set from the UCSC genome browser, which contained 35,519 transcripts and 22,150 genes. ChrY was excluded from this analysis. Promoters for TSSs that were less than 2 kb apart were merged to avoid double counting. The number of tags within each promoter was tallied and divided by the size of the regions and the resulting tag densities for all annotated TSSs were used to compute Pearson correlation coefficients between each pair of samples.

In order to detect regions that were enriched in a neuron-specific manner with the H3K4me3 mark in human samples but not in chimpanzee or macaque, we first filtered a set of 34,639 peaks (without chr Y) obtained by  running MACS on each of the 7 human adult samples against an input human sample (micrococcal nuclease digestion without anti-H3K4me3 antibody pull down) and taking the union of the 7 MACS outputs. The criteria for filtering are as follows: (i) the average tag density for all 11 human samples is higher than 0.01 and is more than 2 times greater than the average tag density for chimpanzee or macaque samples mapped to the human genome. (ii) the region is more than 500 bp long, and (iii) the region is detected as a peak in every human sample. We obtained 418 peaks after the filtering. To obtain human depleted peaks we used a reciprocal approach where initial peaks were detected in chimpanzee and macaque. This resulted in 63 peaks after filtering.

To evaluate significance of the 418 human-enriched and 63 human-depleted peaks, we applied Poisson statistics (the Poisson distribution has only one parameter, which is the mean called lambda). We compared human against chimp and macaque separately. For each peak, we computed the average reads in chimp and in macaque to determine the respective lambdas for the Poisson distributions. Then we computed a p-value for human vs. chimp and another p-value for human vs. macaque, using the average reads across all human samples within the peak (normalized by total reads within all annotated promoters in that sample). The Benjamini-Hochberg method was used to compute the false discovery rate (FDR) of each peak. Only the peaks with FDR<0.05 in both human-chimp and human-macaque comparisons were kept, and there were 410 human-enriched peaks and 61 human depleted peaks with both FDRs<0.05.

Procedures similar to the ones described above were applied in order to detect chimpanzee-specific regions with significant 2-fold enrichment, or depletion, of H3K4me3.

In an additional, independent analyses we probed the collection of 2,148 neuronal peaks not shared with lymphocytes under the most restrictive criteria, as described in our previous work. We retained a peak if average tag density in human samples were more than 2 times greater than in both chimpanzee and macaque samples. It resulted in 33 peaks (**Table S10**), referred to hereafter and in the main manuscript as ^neu^HP (Human-specific peak selectively enriched in neurons). To test if ^neu^HP were identified because regions were unique to the human genome, we also mapped both macaque and chimp ChIP-seq readouts to their appropriate genomes, panTro2 and rheMac2. To allow direct comparison between peaks in HG19 and panTro2/rheMac2, we calculated tag densities normalized by sequencing depth (**Table S10**)**.**

To evaluate significance of the peaks detected we applied Poisson statistics. A set of appropriate monkey samples was used to assess background distribution. After that, a human sample with the lowest coverage at the particular peak was used to obtain the p-value.

Screenshots of ChIP-seq tracks in **Figure S1** online were normalized by the number of tags that map to 5397 orthologus promoters. Orthologus promoters were defined as +/-2kb regions around HG19 RefSeq TSSs that are uniquely lifted-over both ways between any genomes (HG19/panTro2/rheMac2) with 95% identity. UCSC lift-over tool was used for the conversion between different genomes.

**Overlap with DNA hypomethylated regions (HMR) in male germ cells**

Approximately 76,000 DNA hypomethylated sequences (HMR) in human and 70,000 HMRs in chimpanzee sperm were screened for overlap with the 34,639 H3K4me3 peaks of the present study. Altogether 22,808/34,639 neuronal H3K4me3 peaks of the present study overlapped with HMRs in both human and chimpanzee sperm. A subset of 1992 H3K4me3 peak regions from PFC neurons specifically overlapped with sperm HMRs in human but not chimpanzee. Conversely, 669 human PFC neuron H3K4me3 peaks overlapped selectively with HMRs in chimpanzee but not human sperm. Next, 410 peaks out of the 25,469 peaks that overlap with sperm HMRs were picked randomly (10,000 times) and the following expected frequencies were found: human and chimpanzee sperm HMRs (H+/P+) 270/410 = 65.7%, no HMR (H-/P-), 108/410 = 26.6%, H+, P-, 24/410 = 5.7%, H-, P+, 8/410= 1.9%.

**RNA-seq analysis**

Using Tophat software, the first 40bp of each RNA-seq read was mapped into human, chimpanzee and macaque genomes (max. 1mismatch allowed for mapping into native genome and 2 mismatched when non-human was mapped to human. The original 418 hnp peaks were extended and clustered (united overlapped) inside of human genome. Alternatively, the 418 hnp were lifted-over to monkey, extended by 2kb and clustered. Data were expressed as (i) Chimp_HG19/Human_HG19 - sequencing depth normalized ratio; plus pseudocount , (ii) Macaque_HG19/Human_HG19 - sequencing depth normalized ratio; plus pseudocount ; (ii) Chimp_PT2/Human_HG19 - sequencing depth normalized ratio; plus pseudocount; plus normalization by region size; (iv) Macaque_RM2/Human_HG19 - sequencing depth normalized ratio; plus pseudocount; plus normalization by region size (**Table S18**).

Screenshots of RNA-seq track (**Figure S2**) were normalized by sequencing depth. Data for macaque RNA expression were downloaded as a wig file from GSE24538. Data for chimpanzee RNA expression were downloaded from GSE30352 (samples “ptr br M 2,3,4”). Three samples were pulled together and mapped to panTro2 genome allowing up to 2 mismatches.

**Primate alignments to calculate nucleotide substitution rates**

For nucleotide sequences used in the baseml analysis, peak sequences were obtained in humans using the coordinates listed in **Table S10**. UCSC’s liftover utility was used to obtain sequences in 3 additional primate species: chimpanzee, orangutan, and macaque. These sequences were then aligned using clustalW, with default settings.

For amino acid sequences used in codeml analyses, gene sequences were obtained using the BioMart tool available through Ensembl. Sequences were retrieved from five primate species: human, chimpanzee, orangutan, gorilla, and macaque. The Ensembl Genes 64 database was used for all species. Briefly, human sequences were obtained by setting the gene filter to use WikiGene Names, and the “Sequence” attribute set to coding sequences. The “Homologs” attribute was used to return Ensembl Gene IDs for the orthologous sequences in the remaining 4 primate species. These IDs were then queried under each species gene dataset in the same way as humans, except the gene filter was set to Ensembl Gene IDs, instead of WikiGene Names. These sequences were then aligned using PRANK with the codon option, which uses the empirical codon model of Kosiol et al..

**Nucleotide substitution rates in humans**

Baseml from PAML version 4.4 was used to determine nucleotide substitution rates for the primate nucleotide sequence alignment. Two rate classes were specified for the nucleotide sequence alignments. This was accomplished by setting the clock parameter in the baseml control file equal to 2, and then numbering the foreground branch in the tree file according to the numbering scheme explained in the PAML documentation. A likelihood ratio test with ω and p value < 0.01 was used to determine significance. With other words, two classes of substitution rates are allowed for the tree: one foreground rate for the branch of interest and one background rate for the remaining branches. In the null model, the foreground rate is set to equal 1 while the background rate is estimated by the program. In the alternative model, the foreground rate is also estimated, but can differ from the background rate for the rest of the tree. A likelihood ratio test is used to compare the fit of the two models, and a p value of less than 0.01 is considered significant.

**Amino acid substitution rates in humans**

Codeml from PAML version 4.4 was used to analyze the primate amino acid sequence alignments for differences in dN/dS, using maximum likelihood algorithms. For the sites model three model comparisons were used: M1a vs. M2a, M7 vs. M8, M8a vs. M8 (see PAML documentation for parameter settings). M1a has two subsets of sites, one where ω varies between 0 and 1 and one where ω is fixed at one; in M2a ω can be less than 1, equal to 1, or greater than 1. M7 assumes a beta-distribution for ω between 0 and 1, and M8 adds an additional class of sites to M7 with ω>1. In M8a this additional class is fixed at ω=1. Thus, M2a and M8 allow selection in each comparison, while M2, M7, and M8a fit the data to a neutral model. A maximum likelihood ratio is computed for each model, and the null and selection models are compared via a likelihood ratio test, with ω for M1a vs. M2a, and M8a vs. M8, and for M7 vs. M8. For the branch model, two rate classes were specified for the amino acid sequence alignments, with the same specifications as in the baseml branch model except that the model parameter was set to equal 2 instead of the clock. A likelihood ratio test with and a p value < 0.01 was used to determine significance. Neutral, purifying, or positive selection is ‘measured’ by dN/dS, the ratio of nonsynonymous to synonymous amino acid substitutions, with dN/dS equal to, less than, or greater than 1, respectively.

**Sweep analyses**

*SNP dataset:* SNPs were obtained for 176 Yoruban individuals from the 1000 genomes May 2010 merged SNP call release (ftp://ftp-trace.ncbi.nih.gov/1000genomes/ftp/release/20110521/). Ancestral alleles were filled in using the 6 way EPO human ancestral alignment for GRCh37 (ftp://ftp.1000genomes.ebi.ac.uk/vol1/ftp/technical/reference/). Sites were omitted if no ancestral allele was identified. If there were no ancestral alleles for all sites within a region, that region was omitted from further analysis. *Sweep*: Regions were analyzed using Kim and Stephan’s clsw program. The regions were split into windows of 100bp and a likelihood ratio (LR) is calculated for each, along with an estimate of alpha (the selection coefficient) and the most likely location of the target of selection for each window. The window containing the maximum LR is considered the ultimate location of a sweep, if one has occurred. To determine this, neutral simulations were performed using ms. Theta (the mutation rate parameter) was estimated by clsw for each region, and the human-like value for rho (the recombination parameter) was taken from Nielsen et al 2009. Any regions with LR values falling within the top 5% of the LR distribution from neutral simulations were considered significant. For these significant regions, the goodness-of-fit test (GOF) from Jensen et al. 2005 was applied to distinguish between regions that rejected neutrality due to true selection, and those that rejected neutrality because of confounding demographic factors.

**Comparative analyses of human-specific alterations in Ensembl**

Coordinates of human(neuron)-specific H3K4me3 peaks (referred to as hnp in the main manuscript) were converted to the Genome Reference Consortium(GCR)’s genome build Grch37 and Human Specific Alterations (HSA) were selected based on Ensembl EPO primate alignments. Altogether 1519 HSAs were identified (continuous indels in the region were considered as 1 HSA) for the subset of 33 ^neu^HP with both species-specific and cell-type specific (present in neurons but not blood or non-neuronal brain cells.) From these, 915 were found to be conserved in primates tested (*Pan troglodytes, Gorilla gorilla, Pongo abelii, Macaca mulatta*) and 963 were located within large-scale regions with regulatory properties (Ensembl “Regulatory Build”). We downloaded *neanderthal* and *denisova* genomes from UCSC browser in bam alignment format and further checked 1519 HSAs in comparison to them.
The *neanderthal* genome (ftp://hgdownload.cse.ucsc.edu/gbdb/hg19/neandertal/seqAlis) was in HG19 coordinates, *denisova* (ftp://hgdownload.cse.ucsc.edu/gbdb/hg18/denisova)in HG18.
The coordinates of HSAs were lifted-over with UCSC lift-over tool. The comparison to alignments was done with a set of scripts written in Perl. For denisova we found 52 (1353) HSAs that differ (similar) to human genome, for neanderthal - 20 (674) different (similar). For the remaining HSAs: 114 in case of comparison to denisova, and 674 - neanderthal we could not asses the allelic state due to incomplete coverage of archaic genomes.

**Gel shift assays**

Native gel electrophoretic mobility-shift assay with ^32^P end-labeled DNA probes (1X or 50 nM) and Hela nuclear extract (20mg) for 20 minutes at 22^o^C in binding buffer containing 10mM Tis-HCl (pH7.5), 50mM NaCl, 0.5mM DTT (1X). Probe is a 21-bp human or gorilla GATA-1 sequence duplex DNA (CCAGTAAGAA(A human/T other primate)GATTAGCCAG), non-specific probe is 5‘ ATTCGATCGGTTCGGGGCGAGC 3‘ sequence duplex DNA. To demonstrate the specificity, cold probe or non-specific duplex DNA was used at 400X/20,000nM over ^32^P end-labeled DNA probes. All native gels were run at 150 V for 150 min in cold room in the presence of 6% glycerol, dried for 2 hours at 80^o^C and exposed to X-ray film overnight at -70^o^C. For binding stringency experiment same nuclear protein and probe concentrations were used with increased concentrations of sodium chloride in binding buffer (50, 100, 150 and 225 mM).

**Chromosome conformation capture (3C)**

To map physical interactions and loop formations between non-neighboring chromatin fragments, 1000mg of frontal pole tissue from 4 adult human specimens was used (male and female, 7 hrs autolysis interval (median), ranging in age from 30 to 70 years) in conjunction with our 3C protocol as described and 24 primers positioned on the *DPP10* (2q14.1) sense strand 5’ to 3, and 8 primers for the 16p11.2 region.

Presence of physical interactions was determined by sequence-verified PCR product. Control PCRs included no input (‘water’) and also DNA from chromatin digested with Hind III but without the subsequent religation step (‘no T4 ligase’). Additional 3C-qPCR reactions were performed using the QuantiTect Probe PCR Kit (Qiagen) and custom-made FAM-TAMRA taqman probes .

Similar studies were conducted on prefrontal cortex from 3 adult macaques, using tissue from the right hemisphere (left hemisphere of the same animals was used for ChIP-seq). Another set of 18 macaque 3C primers was used to probe 3C in the macaque *DPP10* locus while primer pair 2/7 from human 16p11.2 was used to test the homologous sequences in the macaque. As an additional control, 3C assays were also performed with the H9 embryonic stem cell line. Embroid bodies were generated from colonies grown on feeder cells and grown in low-adherence flasks for 3 days until harvested or re-suspended in Neural Induction media and differentiated to a mixture of neural precursors and postmitotic cells, using a modified protocol.

**Loc389023 cloning, expression and RNA immunoprecipitation**

Loc389023 expression in human brain samples and various cell lines was check either with nuclear only RNA or cytosolic enriched RNA. Two sets of primers (listed below) were used to confirm the expression. Resulted PCR products were further confirmed by sequencing. Full length Loc389023 RNA from human brain fetal nuclei was amplified using 3’end gene specific primer and cloned in pCDNA4A under CMV promoter. Loc389023 expression was verified in HEK293, Hela and SK-N-MC neural crest derived cells. RNA immuno-precipitation (RIP) was carried out as described (Zhao *et al.,* 2008). Briefly, after transfection; nuclei were isolated from SK-N-MC cells using ultracentrifugation. Nuclei pellet was resuspended in 1 ml ice cold lysis buffer (100mM KCl, 5mM MgCl2, 10mM HEPES, 0.5% NP40 along with RNAse inhibitor, lysates were mechanically lysed by passing through 27.5guage needle few times. Nuclear lysates were further diluted in 50mM Tris-HCL, 150mM NaCl and 1mM MgCl2 and pre-cleared before incubating for 6hrs with respective antibodies (H3K4 07-736, IgG 12730 : Upstate, SUZ12 3737, EZH2 4905 : Cell signaling technologies). Input was saved for transfection efficiency analysis. Next day after pull down with protein G beads and several washes; RNA was isolated using Trizol reagent (Invitrogen) according to manufacturer’s protocol. One step quantitative RT-PCR was performed using Quantifast SYBR kit (Qiagen). Primer sequences used for Loc389023 are 1. Left 5’*TCAACACTTGGAAGAAGGGAGCTG*3’ Right 5’*GCCAGTACACCTTATTCTGACCCA*3’; 2. Left 5’*AATCCAGCCCAGATTCTCCTACCA*3’ Right 5’*TTGGGAAGGGCAGTCTGATTGAAG*3’.

**In Situ Hybridization**

In situ hybridization 15 micron thick section from immersion-fixed human PFC specimens was performed as described previously.  DIG-labeled LNA oligonucleotide probes used are as follows: LOC89023 (5DigN/TTGGCTCACTCACTTACTTGCA/3Dig_N), Beta-actin (5DigN/CTCATTGTAGAAGGTGTGGTGCCA/3Dig_N) (Exiqon, Woburn, MA).

**REFERENCES**

1. Cheung, I., et al., *Developmental regulation and individual differences of neuronal H3K4me3 epigenomes in the prefrontal cortex.* Proc Natl Acad Sci U S A, 2010. **107**(19): p. 8824-9.

2. Connor, C., et al., *A simple method for improving the specificity of anti-methyl histone antibodies.* Epigenetics, 2010. **5**(5): p. 392-5.

3. Jiang, Y., et al., *Isolation of neuronal chromatin from brain tissue.* BMC Neurosci, 2008. **9**: p. 42.

4. Matevossian, A. and S. Akbarian, *Neuronal nuclei isolation from human postmortem brain tissue.* J Vis Exp, 2008(20).

5. Shulha, H.P., et al., *Epigenetic signatures of autism: trimethylated H3K4 landscapes in prefrontal neurons.* Arch Gen Psychiatry, 2012. **69**(3): p. 314-24.

6. Molaro, A., et al., *Sperm methylation profiles reveal features of epigenetic inheritance and evolution in primates.* Cell, 2011. **146**(6): p. 1029-41.

7. Liu, Y., et al., *Ab initio identification of transcription start sites in the Rhesus macaque genome by histone modification and RNA-Seq.* Nucleic Acids Res, 2011. **39**(4): p. 1408-18.

8. Brawand, D., et al., *The evolution of gene expression levels in mammalian organs.* Nature, 2011. **478**(7369): p. 343-8.

9. Yang, Z., *Likelihood ratio tests for detecting positive selection and application to primate lysozyme evolution.* Mol Biol Evol, 1998. **15**(5): p. 568-73.

10. Thompson, J.D., T.J. Gibson, and D.G. Higgins, *Multiple sequence alignment using ClustalW and ClustalX.* Curr Protoc Bioinformatics, 2002. **Chapter 2**: p. Unit 2 3.

11. Yang, Z., et al., *Codon-substitution models for heterogeneous selection pressure at amino acid sites.* Genetics, 2000. **155**(1): p. 431-49.

12. Loytynoja, A. and N. Goldman, *An algorithm for progressive multiple alignment of sequences with insertions.* Proc Natl Acad Sci U S A, 2005. **102**(30): p. 10557-62.

13. Kosiol, C., I. Holmes, and N. Goldman, *An empirical codon model for protein sequence evolution.* Mol Biol Evol, 2007. **24**(7): p. 1464-79.

14. Yang, Z., *PAML 4: phylogenetic analysis by maximum likelihood.* Mol Biol Evol, 2007. **24**(8): p. 1586-91.

15. Wong, W.S., et al., *Accuracy and power of statistical methods for detecting adaptive evolution in protein coding sequences and for identifying positively selected sites.* Genetics, 2004. **168**(2): p. 1041-51.

16. Swanson, W.J., R. Nielsen, and Q. Yang, *Pervasive adaptive evolution in mammalian fertilization proteins.* Mol Biol Evol, 2003. **20**(1): p. 18-20.

17. Kim, Y. and W. Stephan, *Detecting a local signature of genetic hitchhiking along a recombining chromosome.* Genetics, 2002. **160**(2): p. 765-77.

18. Hudson, R.R., *Generating samples under a Wright-Fisher neutral model of genetic variation.* Bioinformatics, 2002. **18**(2): p. 337-8.

19. Jensen, J.D., et al., *Distinguishing between selective sweeps and demography using DNA polymorphism data.* Genetics, 2005. **170**(3): p. 1401-10.

20. Jiang, Y., et al., *Setdb1 histone methyltransferase regulates mood-related behaviors and expression of the NMDA receptor subunit NR2B.* J Neurosci, 2010. **30**(21): p. 7152-67.

21. Li, X.J. and S.C. Zhang, *In vitro differentiation of neural precursors from human embryonic stem cells.* Methods Mol Biol, 2006. **331**: p. 169-77.

22. Mellios, N., et al., *A set of differentially expressed miRNAs, including miR-30a-5p, act as post-transcriptional inhibitors of BDNF in prefrontal cortex.* Hum Mol Genet, 2008. **17**(19): p. 3030-42.
